# Comparative and phylogenetic analysis of a novel family of *Enterobacteriaceae*-associated genomic islands that share a conserved excision/integration module

**DOI:** 10.1038/s41598-018-28537-0

**Published:** 2018-07-06

**Authors:** Alejandro Piña-Iturbe, Diego Ulloa-Allendes, Catalina Pardo-Roa, Irenice Coronado-Arrázola, Francisco J. Salazar-Echegarai, Bianca Sclavi, Pablo A. González, Susan M. Bueno

**Affiliations:** 1 Millennium Institute on Immunology and Immunotherapy, Departamento de Genética Molecular y Microbiología, Facultad de Ciencias Biológicas, Pontificia Universidad Católica de Chile, Santiago, Chile; 2 Laboratoire de Biologie et Pharmacologie Appliquée, Centre National de la Recherche Scientifique UMR 8113, École Normale Supérieure Paris-Saclay, Cachan, France

## Abstract

Genomic Islands (GIs) are DNA regions acquired through horizontal gene transfer that encode advantageous traits for bacteria. Many GIs harbor genes that encode the molecular machinery required for their excision from the bacterial chromosome. Notably, the excision/integration dynamics of GIs may modulate the virulence of some pathogens. Here, we report a novel family of GIs found in plant and animal *Enterobacteriaceae* pathogens that share genes with those found in ROD21, a pathogenicity island whose excision is involved in the virulence of *Salmonella enterica* serovar Enteritidis. In these GIs we identified a conserved set of genes that includes an excision/integration module, suggesting that they are excisable. Indeed, we found that GIs within carbapenem-resistant *Klebsiella pneumoniae* ST258 KP35 and enteropathogenic *Escherichia coli* O127:H6 E2348/69 are excised from the bacterial genome. In addition to putative virulence factors, these GIs encode conjugative transfer-related proteins and short and full-length homologues of the global transcriptional regulator H-NS. Phylogenetic analyses suggest that the identified GIs likely originated in phytopathogenic bacteria. Taken together, our findings indicate that these GIs are excisable and may play a role in bacterial interactions with their hosts.

## Introduction

Genomic Islands (GIs) are horizontally transferred DNA segments integrated into bacterial chromosomes^1^. They are characterized by a G + C content, codon usage bias and dinucleotide frequencies, among other sequence signatures, which usually differs from those of the genome^2^. Many of them are found integrated at the 3′-end of tRNA and tmRNA genes^3^, although different families of GIs can show preference for other genes as integration sites^4–7^. GIs range in size from approximately 10 to 500 kbp^8,9^ and encode sets of genes that encompass a wide range of functions for the host bacterium, such as niche colonization, catabolism of diverse substrates, symbiotic relationships, resistance to antimicrobial agents or enhanced virulence^10^. Many GIs contain an excision/integration module that includes an integrase gene, usually belonging to the tyrosine recombinase family^1,11^ and a Recombination Directionality Factor (RDF), which is a small protein of approximately 60–180 amino acids^12^. Together, these proteins can promote recombination reactions between Direct Repeated Sequences (DRS), also known as Left and Right attachment sites (*attL* and *attR*) at both ends of GIs. Recombination leads to GI excision from the chromosome and the consequent formation of a circular episomal element that carries one copy of the DRS (*attP*), while another DRS (*attB*) remains in the host DNA^5,13–15^. After excision the *attB* and *attP* sites can act as substrates for integrase-mediated recombination, resulting in the re-integration of the GI into the bacterial chromosome^16^. In addition to this, excised islands can also be transferred to other hosts by exploiting co-resident prophages for high-frequency transduction inside their capsids^17^, or transferred by conjugation^18,19^. There is evidence that some GIs are replicative in their circular form^20–22^ and that others lack this feature^23^.

Importantly, GIs are susceptible to the loss and gain of genes during their dissemination from one bacterium to another. However, genes encoding the key functions of excision/integration, mobilisation and their regulation remain as a conserved core, as reported for different families of GIs such as the Mobilisable Genomic Islands^5^ and the SXT/R391 family of integrative and conjugative GIs^4^ present in different Gram-negative bacterial families^4,24^, or the conjugative and mobilisable elements recently found in *s*treptococci^6,7^ and the Phage-Inducible Chromosomal Islands (PICIs) of *Staphylococcus aureus* and other Gram-positive strains^17^.

The Region of Difference 21 (ROD21) is an excisable pathogenicity island that has been shown to be important for the virulence of the food-borne pathogen *Salmonella enterica* subsp. *enterica* serovar Enteritidis (*Salmonella* ser. Enteritidis)^15,25,26^, one of the most prevalent serotypes of *Salmonella* in humans and other hosts, such as poultry^27–29^. This genomic island was identified by Thomson *et al*. (2006), in a study that searched for genomic regions that were present in the genome of *Salmonella* ser. Enteritidis, but absent in the genome of *Salmonella* ser. Typhimurium^25^. Genes contained within ROD21 encode potential virulence factors, such as the TlpA protein^15^, which has a toll/interleukin-1 receptor (TIR) domain^26^. A previous study has shown that this protein is involved in bacterial survival within macrophages by disrupting intracellular signaling events that coordinate NF-κB activation and IL-1β secretion^26^. Furthermore, recent studies from our group have shown that changes in the excision rate of ROD21 affect the virulence of *Salmonella* ser. Enteritidis, since mutant strains with reduced rate of excision take more time to cause 100% of mortality in mice, as compared to the wild type strain, likely as a result of changes in the expression of the genes located within the island. In addition, expression of some genes within ROD21, including *tlpA*, showed at least a 3-fold increase when the strain has a reduced or blocked ability to excise the island^30^.

Due to the features described above for ROD21, we decided to perform a computational analysis to identify specific GIs related to this excisable pathogenicity island in order to determine whether these elements are present among other pathogenic members of the *Enterobacteriaceae* family and could thus play a role in their virulence. Since many GI databases use bacterial genomes stored within the RefSeq database for genomic island search, which limits the number of genomes interrogated for island identification, we used a BLASTn-based approach to search a non-redundant database that harbors a larger set of genomes. Using this approach, we found and analyzed different genomic islands within pathogenic and non pathogenic *Enterobacteriaceae* that share a conserved syntenic core with ROD21. These GIs share conserved genes encoding the excision/integration processes of the islands and the majority also share genes encoding putative proteins that are likely involved in conjugal transfer. Importantly, we experimentally corroborated that these islands are able to excise from the chromosome, as previously observed in *Salmonella* ser. Enteritidis^15^. Additionally, some of the GIs identified here were found to carry genes encoding TIR-domain containing proteins and homologues of the global regulator H-NS. Phylogenetic analysis revealed that these islands represent a novel family of GIs present among animal- and plant-pathogenic members of the *Enterobacteriaceae* family.

## Results

### Identification of putative excisable genomic islands with a ROD21-like excision/integration module among pathogenic *Enterobacteriaceae*

**Figure 1:**
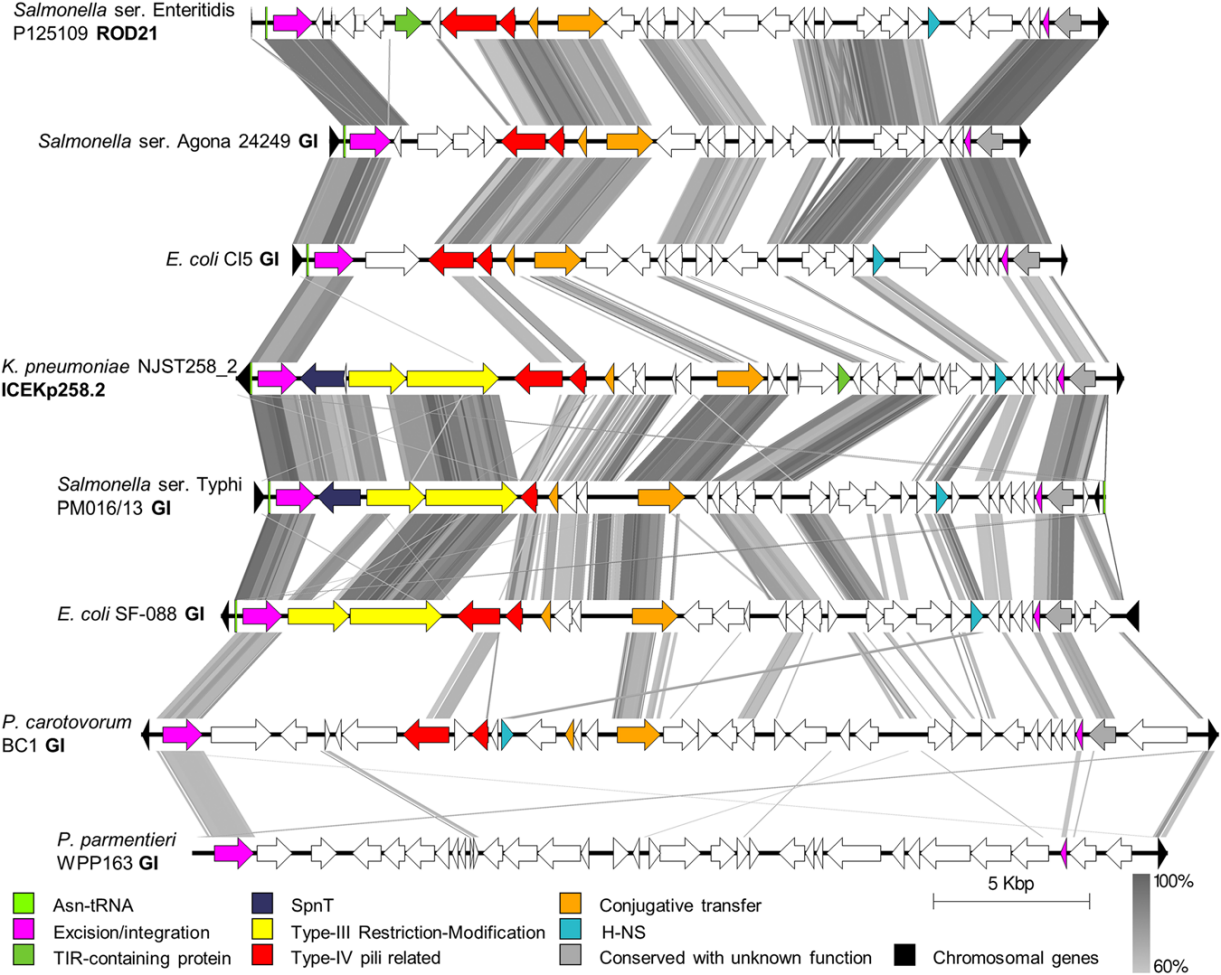
EARL genomic islands have a conserved excision/integration module. Sequence comparison (all three-frames translated for each DNA strand) of genomic islands carried out with tBLASTx. This figure shows eight representative GIs. Gray lines indicate regions with 60–100% identity. Note that the islands of *P*. *carotovorum* BC1 and *P*. *parmentieri* WPP163 are not inserted at Asn-tRNA genes (the green bars in the left side of the islands).

Since integrases and DRS are key factors for the excision of GIs, we decided to perform BLASTn searches using as query a nucleotide sequence from the excisable pathogenicity island ROD21. This 285 nucleotide-long sequence includes the *attL* DRS of ROD21 and the promoter and first 82 nucleotides of the coding sequence of its integrase gene (*SEN1970*). This BLASTn search allowed us therefore to identify putative excisable islands (See Methods). A total of 56 GIs associated with the 3′-end of Asn-tRNA genes were identified among 335 genomes (Table 1). The bacterial genomes found to have ROD21-like GIs belong to some strains of 18 bacterial species from 12 different genera, all members of the *Enterobacteriaceae* family (Table 1). In *Salmonella enterica*, we found islands of interest in 11 different serovars, corresponding to serogroups O:2(A), O:4(B), O:9(D_1_) O:38(P), O:44(V), O:3,10(E_1_) and O:54. Although the size of the GIs ranges from 19 kb (*Photorhabdus luminescens* TTO1), to 41 kb (*Pectobacterium atrosepticum* SCRI1043), the majority (73%) of the islands identified have sizes ranging between 21 and 30 kb. These islands are inserted in different Asn-tRNAs but share highly similar DRS with >85% of identitiy and sizes from 24 to 37 bp (Table S5 in Supplementary File S1). Interestingly, a small number of the ROD21-like GIs seems to be inserted in one of the attachment sites of a different GI located at the 3′-end of an Asn-tRNA gene (e.g. in *P*. *carotovorum* BC1 and *P*. *parmentieri* WPP163, Fig. 1).

**Table 1:** *Enterobacteriaceae*-associated ROD21-like genomic islands.

Bacterium	Accession N°	Island Length (bp)	Islander^a^	Literature^b^	H-NS^c^ (aa)	H-NS-t^d^ (aa)	Tcp^e^ (aa)
*Photorhabdus luminescens* subsp. *laumondii* TTO1	BX571865.1	18,997	—	—	—		—
*Salmonella* serovar Typhi ERL024120	LT906494.1	19,825	—	—	—		—
*Salmonella* serovar Typhi P-stx-12	CP003278.1	20,309	Sen353.21N	—	—		—
*Klebsiella michiganensis* M1	CP008841.1	20,363	—	—	—	78	—
*Escherichia coli* S1	CP010226.1	20,717	—	—	—		—
*Escherichia coli* M8	CP019953.1	20,970	—	—	134	80	—
*Salmonella* serovar Inverness ATCC 10720	CP019181.1	21,047	—	—	—		—
*Serratia marcescens* UMH3	CP018925.1	21,268	—	—	—	78	—
*Salmonella* serovar Agona 24249	CP006876.1	21,485	Sen74.21N	—	—		—
*Salmonella* serovar Borreze SA20041063	CP019407.1	21,485	—	—	—		—
*Escherichia coli* Ecol_517	CP018965.1	21,622	—	—	134	80	—
*Escherichia coli* MEM	CP012378.1	21,706	—	—	—		—
*Escherichia coli* GE3	CP012376.1	22,458	—	—	134	80	—
*Salmonella* serovar Typhi Ty2	AE014613.1	22,588	Sen355.23N	^63^	—		—
*Salmonella* serovar Sloterdijk ATCC 15791	CP012349.1	22,804	—	—	134	80	169
*Escherichia coli* CI5	CP011018.1	23,855	—	—	134	81	—
*Salmonella* serovar Typhi M223	LT904854.1	23,951	—	—	—		—
*Escherichia coli* D7	CP010150.1	24,691	—	—	134	80	—
*Salmonella* serovar Quebec S-1267	CP022019.1	24,817	—	—	134		—
*Cedecea neteri* FDAARGOS_392	CP023525.1	25,678	—	—	—	90	—
*Escherichia coli* 0127:H6 E2348/69	FM180568.1	25,819	Eco626.26N	IE3^64^	—	80	—
*Salmonella* serovar Typhi PM016/13	CP012091.1	26,314	—	—	134		—
*Salmonella* serovar Pullorum ATCC 9120	CP012347.1	26,359	Sen215.26N	—	134		293
*Salmonella* serovar Gallinarum 287/91	AM933173.1	26,404	Sen212.27N	ROD21^25^	134		293
*Salmonella* serovar Enteritidis P125109	AM933172.1	26,496	Sen204.27N	ROD21^25^	134		293
*Salmonella* serovar Dublin CT_02021853	CP001144.1	26,498	Sen82.26N	ROD21^65^	134		293
*Salmonella* serovar Nitra S-1687	CP019416.1	26,500	—	—	134		293
*Citrobacter freundii* 18–1	CP022273.1	27,132	—	—	—		169
*Salmonella* serovar Anatum USDA-ARS-USMARC-1175	CP007483.2	27,139	—	—	134		—
*Escherichia coli* ETEC-2265	CP023346.1	27,200	—	—	134		—
*Escherichia coli* YD786	CP013112.1	27,239	—	—	134	80	—
*Enterobacter* sp. FY-07	CP012487.1	27,410	—	—	134		—
*Klebsiella pneumoniae* 30684/NJST258_2	CP006918.1	27,495	—	ICEKp258.2^66^	134		139
*Pectobacterium wasabiae* CFBP 3304	CP015750.1	27,690	—	—	133	90	—
*Pectobacterium atrosepticum* SCRI1043	BX950851.1	28,081	Pat3.28N	HAI13^67^	133	83	—
*Escherichia coli* SF-088	CP012635.1	28,452	—	—	134		—
*Klebsiella pneumoniae* strain AR_0148	CP021950.1	28,777	—	—	134		—
*Serratia marcescens* SM39	AP013063.1	28,938	—	—	134		—
*Serratia marcescens* CAV1492	CP011642.1	29,010	—	—	134		—
*Kluyvera intermedia* CAV1151	CP011602.1	29,250	—	—	134		—
*Escherichia coli* ED1a	CU928162.2	29,355	—	—	134		—
*Klebsiella pneumoniae* 1084	CP003785.1	29,853	Kpn29.30N	—	134		—
*Yersinia intermedia* Y228	CP009801.1	30,275	—	—	—	83	—
*Pectobacterium parmentieri* WPP163	CP001790.1	30,288	Pwa2.30N	—	—		—
*Raoultella ornithinolytica* Yangling I2	CP013338.1	30,440	—	—	134		—
*Escherichia coli* ABWA45	CP022154.1	30,538	—	—	—		—
*Yersinia rohdei* YRA	CP009787.1	30,663	—	—	—	81	—
*Pectobacterium parmentieri* RNS08.42.1 A	CP015749.1	33,037	—	—	133(2)	80	—
*Pectobacterium carotovorum* subsp. *brasiliense* BC1	CP009769.1	33,571	—	—	133	86	—
*Pectobacterium parmentieri* RNS08.42.1 A	CP015749.1	33,684	—	—	—		—
*Pectobacterium parmentieri* WPP163	CP001790.1	33,698	Pwa2.34N	—	133	81	—
*Pectobacterium atrosepticum* 21 A	CP009125.1	35,819	—	—	132	87	—
*Pectobacterium wasabie* SCC3193	CP003415.1	37,046	—	—	—		—
*Pectobacterium carotovorum* SCC1	CP021894.1	37,050		—	—		—
*Klebsiella oxytoca* AR_0147	CP020358.1	37,983	—	—	—		—
*Pectobacterium atrosepticum* SCRI1043	BX950851.1	40,824	Pat3.41N	HAI7^67^	132	80	—

ROD21 islands found in different serovars of *Salmonella* or other bacterial species are listed in order of decreasing length. Letters in superscript indicate: ^(a)^names of genomic islands already identified in the Islander database or ^(b)^in the literature with their indicated references and ^(c)^the predicted length in amino acids for the full-length and ^(d)^short versions of H-NS homologues, as well as ^(e)^TIR-like domain containing proteins when present. Note: *Salmonella* ser. Pullorum is considered as a biovar of serovar Gallinarum and the Pwa2.30 N island was found only by searching in the Islander database.

Notably, the identified ROD21-like GIs are found in many plant and animal pathogenic bacteria, such as phytopathogens from genus *Pectobacterium*, entomopathogenic *Ph*. *luminescens*, extraintestinal pathogenic *E*. *coli*, antibiotic-resistant clinical isolates of *E*. *coli* and *K*. *pneumonaie* ST258, invasive *K*. *pneumoniae*, and foodborne disease- and typhoid fever-causing *Salmonella* (Table S2 in Supplementary File S1). Each of the 335 genomes harbor one ROD21-like GI, except for the phytopathogens *P*. *atrosepticum* SCRI1043, *P*. *parmentieri* RNS08.42.1 A and *P*. *parmentieri* WPP163, which have two (Table 1).

### ROD21-like genomic islands share a conserved excision/integration module

**Figure 2:**
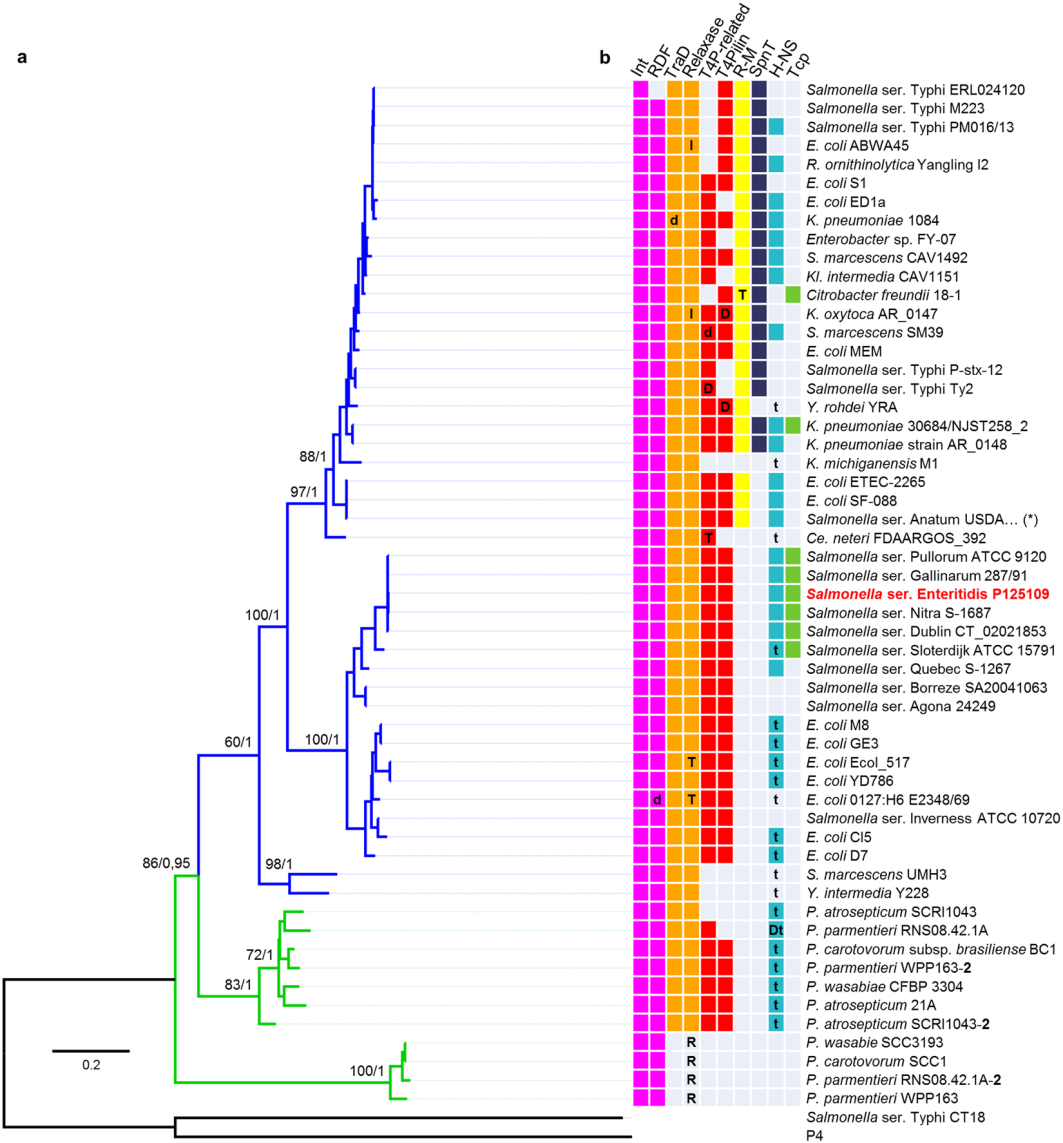
Genomic island phylogeny correlates with the distribution of conserved genes. (**a**) Maximum likelihood tree based on codon-aligned nucleotide sequences of island integrases with support values corresponding to 100 bootstrap replicates and posterior probabilities (BS/p; shown only for basal nodes). The integrase from the SPI7 island and phage P4 were used as outgroups. Blue and green branches correspond to GIs harbored by animal and plant bacterial pathogens, respectively. (**b**) Distribution of conserved genes coloured as in Fig. 1. Bold letters inside the squares indicate whether the corresponding ORF is duplicated (D), disrupted (d), truncated (T) or has an insertion (I). For H-NS, the presence of full-length (colored square) or truncated (t) homologues is indicated. For the relaxases, the presence of a relaxase different from that of the MobA/MobL family is also specified (R). The genome harboring ROD21 is in bold and red font. The number **2** after *Pectobacterium* strains WPP163, SCRI1043 and RNS08.42.1 A is intended to identify the largest of the two EARL GIs harbored by these bacteria. (*)Strain USDA-ARS-USMARC-1175.

Given that the identified ROD21-like GIs share a conserved integration site and DRS, and that the annotations revealed the presence of different conserved genes with similar distribution, we carried out comparisons of the translated open reading frames (ORFs) (six in total, three reading frames on each DNA strand) using tBLASTx. The majority of ROD21-like GIs (52 out of 56) share a conserved set of genes that could be classified in three main groups: a first group includes ORFs encoding putative type-IV pilin and a type-IV pilus-related protein; a second group includes ORFs encoding putative relaxase belonging to the MobA/MobL family and a TraD homologue, and the third group includes ORFs encoding a P4-like integrase from the tyrosine-recombinase family, together with a putative RDF (Fig. 1). Four ROD21-like GIs showed exceptions, namely those found in *P*. *wasabie* SCC3193, *P*. *carotovorum* SCC1, *P*. *parmentieri* RNS08.42.1 A and *P*. *parmentieri* WPP163, which only encode the excision/integration module (Fig. 2). In all cases, the coding sequences of the integrases and putative RDFs are located near to each attatchment site.

Interestingly, in most ROD21-like GIs the putative RDF-coding sequence is accompanied by an 843 bp gene of unknown function. An aspect that is not shared by all ROD21-like GIs, but is present in many of them, is a group of genes encoding putative H-NS and SpnT homologues, Toll/Interleukin-1 receptor (TIR)-domain containing proteins (Tcps) and type-III restriction-modification (R-M) systems (Figs 1, 2 and S5). Regarding the H-NS homologues identified, we found sequences that are predicted to encode full-length (132–134 amino acids), as well as truncated forms of the protein (78–90 amino acids) (Table 1). Notably, 14 of the 33 ROD21-like GIs encoding full-length H-NS homologues also encode a truncated form of this protein, whose coding sequence is located in the opposite direction. On the other hand, six other ROD21-like GIs only encode truncated forms of the H-NS homologues (Fig. 2).

Since the identified ROD21-like GIs are only found in *Enterobacteriaceae* and that the genomic island most studied to date within this group is ROD21, we named this group of GIs the *Enterobacteriaceae*-associated ROD21-like (EARL) genomic islands.

### Phylogenetic analyses of EARL GIs

Because integrases are involved in GI excision/integration and dissemination between bacterial species or genera, we used them as markers to study the evolutionary history of EARL GIs^1^. We performed a reconstruction of EARL GIs phylogeny based on the nucleotide sequences of their cognate integrases. The resulting maximum likelihood tree shows that the integrases form two major clades: one at the bottom of the tree, which clusters EARL GIs present only in phytopathogens, while the other clade clusters EARL GIs from both non-pathogenic and pathogenic strains infecting both animals and plants (Fig. 2a). All the phytopathogenic strains belong to the genus *Pectobacterium*. Interestingly, the subclade that groups the non-pathogenic and pathogenic animal strains branches into two clusters: one includes EARL GIs present in *Escherichia coli* and *Salmonella enterica*, while the other one harbors EARL GIs carried by a greater diversity of species, such as *Klebsiella pneumoniae*, *K*. *oxytoca*, *K*. *michiganensis*, *Raoultella ornithinolytica*, *Kluyvera intermedia*, *Serratia marcescens*, *Yersinia rhodei*, *Y*. *intermedia*, *S*. *enterica* serovars Anatum and Typhi, *Enterobacter* sp., *Citrobacter freundii*, *Cedecea neteri* and *E*. *coli* (Fig. 2). This cluster includes the carpabenem-resistant *K*. *pneumoniae* NJST258-2, which harbors a EARL GI previously denominated ICEKp258.2 (Table 1).

The presence of conserved genes within EARL GIs correlates with the integrase-based EARL GI phylogeny. While all EARL GIs encode an integrase and have RDF genes, as well as DRS (Figs 1 and 2b), the major clades are differentiated by the presence of genes encoding putative proteins related to type-IV pili, conjugative transfer and H-NS homologues (Fig. 2b). In the animal pathogen subclade, phylogeny correlates with the acquisition of genes encoding a type-III R-M system and homologues of the SpnT encoding gene.

**Figure 3:**
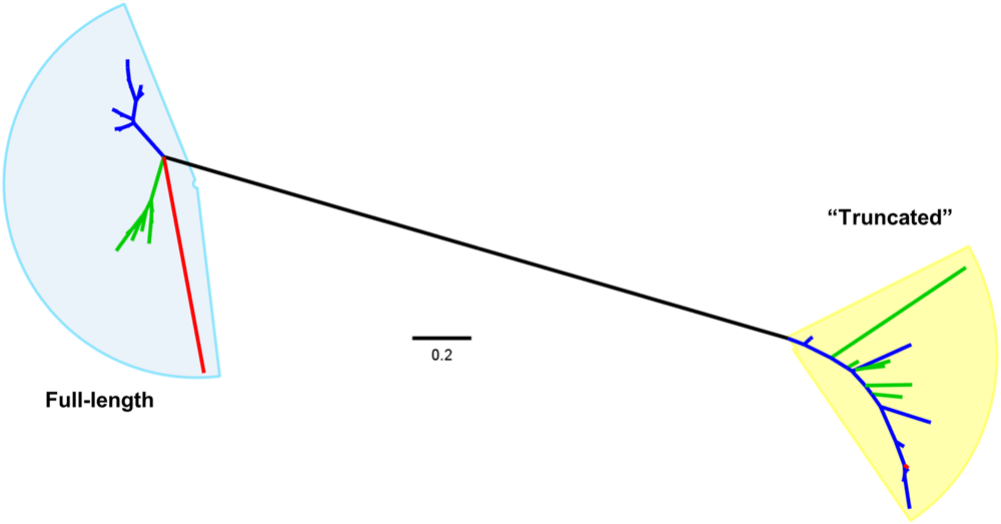
Full-length and truncated *hns* homologues belong to distantly related clades. Unrooted maximum likelihood phylogenetic tree constructed with codon-aligned *hns* homologue sequences found in EARL GIs. Blue and green branches correspond to animal- and plant-associated bacteria, respectively. The red branches represent chromosomal *hns* of *E*. *coli* K-12 strain MG1655 and truncated *hns* carried by the IE3 island of EPEC E2348/69.

Additionally, we constructed a phylogenetic tree based on the *hns* homologues carried by EARL GIs in order to gain a better understanding of the relationships between the full-length forms of these proteins and the truncated ones. The unrooted maximum likelihood tree shows that full-length and truncated forms of *hns* separate into two distantly related clades (Fig. 3). As with the integrase-based phylogeny, the full-length homologues form two sub-clades, comprised by the genes carried by plant and animal pathogenic bacteria. In contrast, the truncated homologues show a different branching pattern, in which *hns* from phytopatogens are more closely related to genes of some animal pathogenic bacteria (Fig. 3). It is noteworthy that full-length homologues are more closely related between themselves than with the chromosomal *hns* encoded by *E*. *coli* K-12 (Fig. 3).

To corroborate that the genomes included in this study were fully syntenic, we calculated the distance of the GI insertion site relative to the chromosomal replication origin (*oriC*) for each EARL GI (Table S2 in Supplementary File S1). Most of the EARL GIs are located far from *oriC*, at 66.4–98.2% (mean = 77.5%) of the maximum possible distance (considered as one half of the chromosome length) (Supplementary Fig. S1). However, the EARL GI from *Salmonella* ser. Inverness ATCC 10720 is located at 39.7% of the maximum distance, probably as a result of a chromosomal rearrangements that locate the Asn-tRNAs near *oriC* (Supplementary Fig. S2).

### Inability to excise ROD21 modulates expression of genes within this pathogenicity island

**Figure 4:**
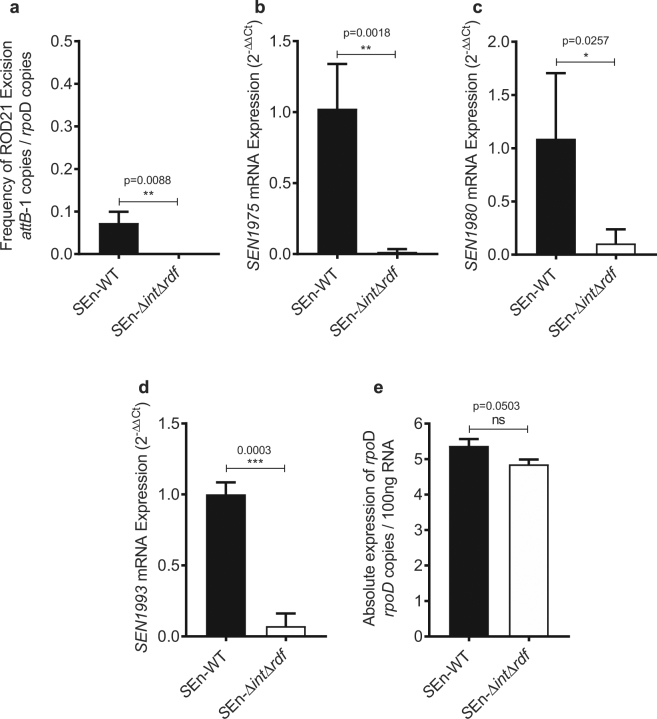
ROD21 excision affects the gene expression inside the island. (**a**) Deletion of the integrase (*SEN1970*) and putative RDF (*SEN1998*) coding sequences from *Salmonella* ser. Enteritis results in impairment of ROD21 excision and reduction in expression of (**b**) *SEN1975*, (**c**) *SEN1980* and (**d**) *SEN1993* under *in vitro* conditions (LB, pH 7.0, 37 °C, OD_600_ = 0.6). (**e**) Expression of *rpoD*, located outside the island, was not affected. Unpaired, two-tailed *t* test was used with α = 0.05. Error bars represent the standard deviation.

In order to corroborate the hypothesis that ROD21 excision modulates the expression of genes within this genomic island, a *Salmonella* ser. Enteritidis strain lacking the genes coding for the  integrase and RDF was generated. As shown in Fig. 4a, deletion of these genes prevents ROD21 excision. Both wild-type and mutant strains were grown in LB under standard laboratory conditions (37 °C, pH 7.0) and RNA was purified to evaluate, by RT-qPCR, the expression of the ROD21 genes *SEN1975*, *SEN1980*, *SEN1993*, which encode the TlpA protein, the putative relaxase and the H-NS full-length homologue, respectively. As show in Fig. 4b–d, impairment of ROD21 excision prevented the proper expression of ROD21 genes in the mutant strain. As a control, expression of the housekeeping gene *rpoD*, which is located on the *Salmonella* ser. Enteritidis genomic core, and outside ROD21, was also evaluated for both strains. We observed no changes in expression of *rpoD* (Fig. 4e), suggesting that the impairment of ROD21 excision affects specifically genes within this island. These results support the notion that ROD21 excision influences expression of genes within the GI.

### The EARL GIs from *K*. *pneumoniae* ST258 strain KP35 and enteropathogenic *E*. *coli* O127:H6 strain E2348/69 are excisable

**Figure 5:**
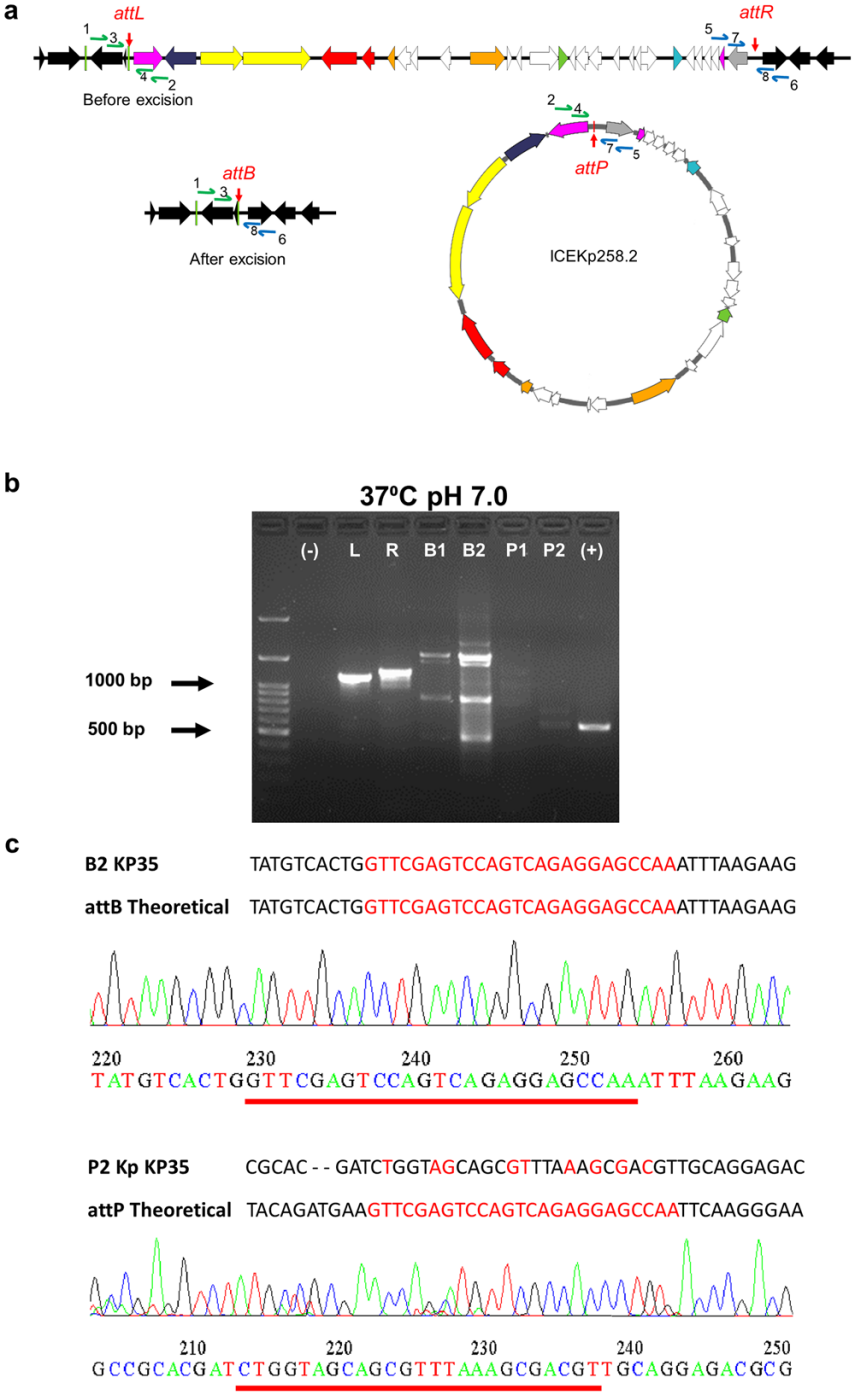
Detection of ICEKp258.2 excision in *K*. *pneumoniae* ST258 KP35. (**a**) Locations of the eight primers used for the nested PCR are indicated with blue and green arrows next to the *attL*, *attR*, *attB* and *attP* sites. Primers 1, 2, 5, 6 and 3, 4, 7, 8 were used for the first and second round in nested PCR, respectively. Amplicons containing *attB* or *attP* can be obtained only if the 27 kb island is excised from the chromosome. Gene colouring is the same as in Fig. 1. (**b**) Agarose gel showing amplification products (L: 1123 bp, R: 1220 bp, B1: 1029 bp, P1:1340 bp, B2: 519 bp, P2: 668 bp) of nested PCR reactions performed using genomic DNA obtained from *K*. *pneumoniae* KP35 grown at 37 °C, pH 7.0. (−), negative control; L: *attL*, R: *attR*, B1: *attB* from first round of PCR, B2: *attB* from second round of PCR, P1: *attP* from first round of PCR, P2: *attP* from second round of PCR; (+) positive control *rpoD* (577 bp) for the PCR reaction. UV exposure time was 400 ms. (**c**) The sequence of PCR products from the second nested PCR round for *attB* and *attP* (B2 and P2) were obtained and compared with theoretical sequences. *att*B and *attP* specific sequences are highlighted in red and chromatograms are shown.

**Figure 6:**
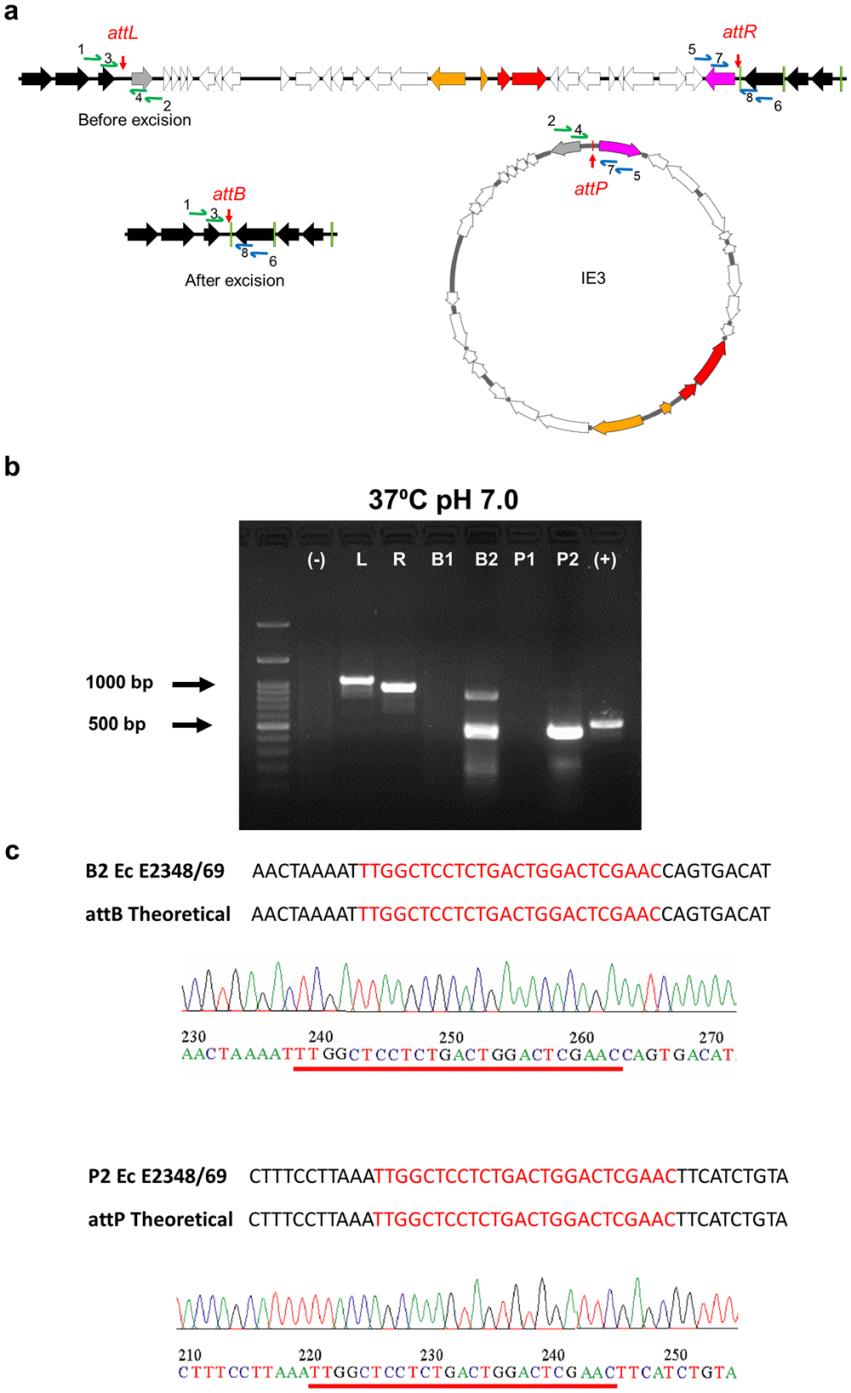
Detection of IE3 excision in EPEC E2348/69. (**a**) Locations of the eight primers used for the nested PCR are indicated with blue and green arrows next to the *attL*, *attR*, *attB* and *attP* sites. Primers 1, 2, 5, 6 and 3, 4, 7, 8 were used for the first and second round in nested PCR, respectively. Amplicons containing *attB* or *attP* can be obtained only if the 26 kb island is excised from the chromosome. Gene colouring is the same as in Fig. 1. (**b**) Agarose gel showing amplification products (L: 1057 bp, R: 937 bp, B1: 985 bp, P1: 1009 bp, P2: 502 bp, B2: 507 bp) of nested PCR reactions performed using genomic DNA obtained from EPEC E2348/69 grown at 37 °C, pH 7.0. (−), negative control; L: *attL*, R: *attR*, B1: *attB* from first round of PCR, B2: *attB* from second round of PCR, P1: *attP* from first round of PCR, P2: *attP* from second round of PCR; (+) positive control *rpoD* (577 bp) for the PCR reaction. UV exposure time was 400 ms. (**c**) The sequence of PCR products from second nested PCR round for *attB* and *attP* (B2 and P2) were obtained and compared with theoretical sequences. *attB* and *attP* specific sequences are highlighted in red and chromatograms are shown.

Since ROD21 can be excised from the bacterial chromosome and this process can be modulated by temperature and pH, we hypothesized that EARL GIs, which share conserved excision/integration modules with ROD21, would also be excisable. For this purpose, the excision of the ICEKp258.2 island from *K*. *pneumoniae* ST258 KP35 and the IE3 island from the enteropathogenic *E*. *coli* O127:H6 (EPEC) E2348/69 was assessed by PCR. After overnight growth in LB broth at 23 °C/pH 7, 37 °C/pH 5.4, and 37 °C/pH 7, genomic DNA was purified and assessed by nested PCR with primers targeting the attachment sites that would result in an amplicon after island excision (Figs 5a and 6a).

For ICEKp258.2 excision, according to the sequence analyses, the PCR products for *attB* and *attP* should be 519 bp and 668 bp long, respectively. As shown in Fig. 5b, amplicons with the expected size were obtained. These amplicons can only be found if the EARL GIs are absent, indicating that the island was excised from the bacterial chromosome. Although the *attB* and *attP* sites were detected under all tested conditions, the product that indicates GI excision was scarcely detectable at 37 °C/pH7 (Fig. 5b). For conditions 23 °C/pH 7 and 37 °C/pH 5.4 see Supplementary Fig. S3. Similar results were obtained for EPEC E2348/69 with amplicons matching the predicted size of 507 bp and 502 bp for *attB* and *attP* (Figs 6b and S4). However, for both bacteria, we found that other amplicons were observed after electrophoresis of the *attB* and *attP* PCR products, suggesting that the primers might produce nonspecific products. Therefore, to corroborate that the PCR products obtained were specifically from the *attB* and *attP* sites as expected, the amplicons were purified and sequenced. As shown in Fig. 5c, the sequence obtained for the PCR product correspond to the predicted *attB* site generated in the chromosome of *K*. *pneumoniae* after ICEKp258.2 excision. However, the sequence of the amplicons obtained for the *attP* site did not match the predicted sequence of ICEKp258.2 *attP* (Fig. 5c). Since several amplicons were obtained after the second round of PCR to detect the *attP* site, it is possible that the PCR conditions used in this assay were not optimal for the amplification of the expected *attP* sequence. On the contrary, for EPEC E2348/69, the sequences obtained for both *attB* and *attP* amplicons were identical to the predicted sites after IE3 island excision (Fig. 6c). These findings support the notion that the EARL elements are excisable islands.

## Discussion

Studies in the PPHGI-1 island of *Pseudomonas syringae* pathovar *phaseolicola* and ours in ROD21 indicate that GI excision from bacterial chromosomes can modulate the expression of genes contained within the islands and influence the virulence of the bacteria that harbor them^15,30–32^. Given the role of ROD21 excision in the virulence of *Salmonella* ser. Enteritidis, we carried out computational analyses to identify similar GIs in the bacterial genomes available at the time of this study. Here, we found that ROD21-like GIs are a novel group of genomic islands encoded by members of *Enterobacteriaceae*, including plant and animal pathogenic strains. Given their presence in this bacterial family, we have designated these GIs as the  *Enterobacteriaceae*-associated ROD21-like genomic islands (EARL GIs). Importantly, the bioinformatics approach performed in this study used the non-redundant nucleotide database of GenBank, which has allowed us to identify EARL GIs among all bacterial genomes available to date (October, 2017). Although there are bioinformatic tools currently available to identify GIs with high accuracy, their prediction by current computational methods still is a challenging task, and existing GIs may be overlooked^2,33^. For instance, the Islander tool (Islander Database of Genomic Islands at Sandia National Laboratories) only identified 12 of the 55 islands identified in this study (Table 1). The reason for these significant differences is that the majority of the bacterial genomes that harbor EARL GIs are not in the RefSeq database used by Islander for GI prediction and thus omission of an important group of bacteria occurs in the analyses. However, Islander found an island which shares the features of EARL islands, namely Pwa2.30N, which could not be identified by our BLASTn search, likely because of a low degree of similarity between its DRS and the query sequence. Nevertheless, we generally found consensus between the location and length of the 12 GIs predicted by Islander and our approach, except for GIs found in *Salmonella* serovars Enteritidis, Gallinarum and Typhi, for which Islander included a tRNA and remnant of integrase genes adjacent to the tRNA flanking the island, which are not part of these islands.

Dissemination of many GIs relies on bacterial conjugation^18,34^ and thus these elements are classified in two groups based on whether they encode the molecular machinery required for their excision and conjugation. While the integrative and conjugative elements (ICEs) encode both sets of genes^35^, the integrative and mobilisable elements (IMEs; also known as mobilisable genomic islands) utilize the transfer machinery encoded by ICEs or conjugative plasmids^36–38^.

Most EARL islands encode a TraD homologue and a putative relaxase of the MobA/MobL (MOB_Q_ superfamily)^39^. Since EARL GIs are not self-conjugative elements, because they don’t encode the Type-IV secretion system (T4SS) required for conjugal transfer, the encoded relaxases may provide the means for island mobilisation by recognizing their cognate origin of transfer (*oriT*) and recruiting the island DNA to a T4SS encoded by a coresident conjugative element^40^. Transfer of ROD21 has been observed from *Salmonella* ser. Enteritidis to mutant strains lacking the island, as well as to *Salmonella* ser. Typhimurium^18^. Experiments assessing ROD21 mobilisation from *Salmonella* ser. Typhimurium LT2 with and without its virulence plasmid pSLT, have shown that transfer of the island is only achieved from bacteria harboring pSLT to a pSLT-positive strain^18^. Therefore, similar mechanisms could be responsible for the horizontal transfer of other EARL GIs.

Importantly, GI acquisition may pose a metabolic burden for bacterial hosts since and increased amount of DNA in the bacterial genome require its replication and maintainance. Furthermore, a lack of regulatory mechanisms for controlling the expression of the newly acquired genes poses metabolic challenges for the bacterium^41^. Here we support the notion that the ability of EARL islands to excise from the chromosome might be a mechanism conserved among these GIs to modulate gene expression. We have demonstrated here that expression of genes within ROD21 is affected in a mutant strain of *Salmonella* ser. Enteritidis unable to excise this GI. However, while we found a reduction in expression of the genes encoding TlpA, the putative relaxase and the H-NS full-length homologue (Fig. 4), a previous work assessing the effects of ROD21 excision found the opposite effect^30^. Differences in the mutant strains might account for observed results. In that work, the strain with reduced excision and the one that lacked this capacity were constructed by deletion of the integrase ORF (*ΔSEN1970*), and the entire region spanning the integrase and the Asn-tRNA which has the *attL* site of the island [*Δ*(*asnT2-SEN1970*)], respectively; while our mutant strain lacks only the integrase and putative RDF (*ΔSEN1970 ΔSEN1998*). Therefore, the absence of *SEN1998* may be causing the different outcome. Previous studies on the *Vibrio* Pathogenicity Islands 1 and 2, which also have their integrase gene located downstream the attatchment site as in ROD21, provided evidence that their RDFs act as transcriptional repressors of the integrase by binding the *att* region which overlaps the integrase promoter^42^. Further research is required to elucidate the relationship between *SEN1998* and gene expression in ROD21. Nevertheless, inability to excise ROD21 has always resulted in altered gene expression inside the island in all mutant strains tested to date. This phenomenon might account for a mechanism used by pathogens to modulate the expression of newly acquired virulence genes, useful in specific stages of their infective cycle^32,43^.

Another mechanism for regulation of gene expression inside GIs is mediated by the heat-stable nucleoid structuring (H-NS) protein, which plays crucial roles due to its ability to recognize and bind to DNA sequences with low G + C content in such a way to form nucleoprotein complexes capable of limiting RNA polymerase activity, in a process designated xenogeneic silencing^41,44–47^. This may explain why most (33/54) of EARL GIs encode predicted full-length H-NS homologues, since they have low G + C content. The presence of such genes could facilitate island dissemination onto new bacterial hosts and its preservation, by providing a regulatory mechanism for EARL GI-gene expression. Further research is required to assess whether these homologues are integrated in the global transcriptional network of their hosts. For example, the Hfp protein, an H-NS homologue encoded in the *serU* island of some UPEC strains participates in the regulation of virulence factors encoded outside the island^48^. It is noteworthy that 20 of the 54 identified islands also carry genes predicted to encode truncated forms of the H-NS protein such as H-NST, encoded in the IE3 island of enteropathogenic *E*. *coli* E2348/69^49^. This 80 amino acid-long protein (compared with the 137 aa full-length H-NS) corresponds to the N-terminal dimerization domain of H-NS, which was shown to interact with H-NS as an antagonist^49^. The H-NST homologues encoded in the other EARL GIs could also be playing a role in relieving H-NS-mediated repression. Our phylogenetic analysis of short and full-length *hns* homologues found in EARL GIs, including *hnsT* of EPEC E2348/69, shows both groups clustering as separate, distantly related clades evidencing that the short ones are not simply remnants that resulted after an event of duplication or acquisition. The differing topology within each clade also suggests that acquisition of the truncated and full-length versions of *hns* homologues by EARL GIs represents two separate, independent events.

Analysis of EARL islands and multiple sequence alignments (Supplementary Fig. S5) allowed us to identify ORFs likely encoding Toll/Interleukin-1 receptor (TIR)-like domain containing proteins (Tcps) in three different islands (Table 1), in addition to the already characterized TlpA protein encoded in ROD21^26^. Bacterial Tcps are known virulence factors able to disrupt the innate immune response by interacting with mammalian TIR domain-containing proteins of the TLR signalling pathway, such as TLRs, IL-1R, MyD88 and TIRAP, thus suppressing transactivation of the NF-κB transcriptional activator, with the consequent alteration of proinflammatory cytokine secretion^50^. Interestingly, we identified an ORF encoding a putative Tcp in the ICEKp258.2 island found in carbapenem-resistant *K*. *pneumoniae* ST258 strains^51^, which may have a role in the resistance of this ST against phagocityc killing^52^. The other two putative Tcp encoded in the GIs from *Salmonella* ser. Sloterdijk ATCC 15791 and *Citrobacter freundii* 18–1 may also play a role in virulence of these pathogens.

As shown in Figs 1 and 2, open reading frames encoding functions related to horizontal transfer (i.e. excision/integration and conjugative transfer genes), as well as other ORFs of unknown function are conserved among EARL GIs, suggesting that an ancestral island gave rise the diversity of EARL GIs. Our phylogenetic analysis, based on the nucleotide sequence of the integrase open reading frames reveals that island gene content correlates with the evolution of the integrase. Based on the phylogenetic reconstruction, we hypothesize that EARL GIs originated in phytopathogenic bacteria and later, through horizontal gene transfer, disseminated to other bacteria, losing some genes and gaining others that are favorable for the recipient bacteria. Interestingly, a closer relationship between EARL GIs is not necessarily correlated with bacterial species that are closer to each other, which suggests that multiple, independent transfer events have occurred more recently than bacterial speciation.

Because most EARL GIs encode intact excision/integration modules, the genomic region should theoretically undergo excision from the chromosome. Excision of EARL GI has so far only been demonstrated for ROD21 in *Salmonella* ser. Enteritidis P125109, which responds to environmental stimuli^15^. Here, we show that the ICEKp258.2 island from *K*. *pneumoniae* ST258 KP35 and the IE3 island found in EPEC E2348/69 are excisable elements. These results support the notion that an important feature of EARL GIs is their capacity to excise from the chromosome. This property may be relevant for the capacity of EARL GIs to mobilise and for modulation of gene expression and virulence, as described for ROD21^18,30^.

With the availability of increasing numbers of sequenced genomes, a great diversity of GIs has been described to date in Gram-positive and Gram-negative bacteria. Some examples of early identified and well studied families of genomic islands are the Phage Inducible Chromosomal Islands (PICIs)^17,53^ and the Staphylococcal Chromosomal Cassette *mec* (SCC*mec*)^54^ in Gram-positive bacteria and the SXT/R391 family in Gram-negative γ-Proteobacteria^4,24^, which encode genes responsible for virulence and antimicrobial resitance. The EARL islands described in this study add to the already substantial and ever-growing number of GIs and GI-families found in bacterial genomes, and share the overall features described in those well characterized GIs: a modular organization and the conservation (among family members) of genes encoding the functions related to excision/integration, transfer and their regulation. Further research is required to assess the role of the genes encoded in this family of islands both when integrated or excised within their respective hosts. The widespread distribution of EARL GIs among pathogenic *Enterobacteriaceae* suggests that they are mobilisable elements and likely active players in bacterial pathogenesis.

## Methods

### Genomic island search and comparative analyses

A BLASTn search^55^ was performed using as query the sequence “*attL* + 82 nt” from *Salmonella enterica* ser. Enteritidis P125109 (Accession N° AM933172.1; nucleotides 2,061,160–2,061,444; 285 nt), spanning the left attachment site of ROD21 (*attL*), the integrase *SEN1970* promoter and the first 82 nucleotides of *SEN1970*. BLASTn was performed by aligning with the non-redundant sequence database. Putative excisable genomic islands were identified in the graphic view of BLASTn alignments as DNA regions flanked by two direct repeated sequences (DRS), visualized as the main BLAST hit (≤285 bp) immediately followed by a putative integrase-encoding gene, and a second hit of approximately 30 bp located in the same orientation in a noncoding region. Bacterial strains, accession numbers and island coordinates were recorded. Sequences harboring the putative islands were downloaded from GenBank. Nucleotide comparisons of each island with ROD21 and ICEKp258.1 were performed using tBLASTx^56^ with the standalone BLAST v2.6.0+ software run in EasyFig v2.2.2^57^ and then visualized and analyzed using EasyFig and Artemis Comparison Tool v13.0.0^58^.

### Phylogenetic analyses

Nucleotide sequences of integrases encoded in each island were downloaded from GenBank (Supplementary Information). Codon alignment was performed using MUSCLE in MEGA v7^59^ and gapped columns were deleted (the nucleotide sequence obtained from the *Ph*. *luminescens* TTO1 integrase harbors nonsense mutations and was therefore excluded from the alignment). The IQ-Tree web server (http://iqtree.cibiv.univie.ac.at/)^60^ was used for the selection of the best-fitting nucleotide substitution model (SYM + G), according to the corrected Akaike Information Criterion. The SYM + G model was then applied for construction of maximum likelihood (IQ-Tree web server) and Bayesian (Mr. Bayes v3.2.6)^61^ trees, using as outgroups the integrases of SPI7 harbored by *Salmonella* ser. Typhi CT18 and of phage P4 (Supplementary Information). Two outgroups were used for the ML tree and one (SPI7 integrase) for the Bayesian tree. Node support was obtained from 100 bootstraps (ML) and posterior probabilities (Bayesian). FigTree v1.4.3 was used for tree visualization and colouring. Maximum likelihood phylogeny for H-NS homologues was constructed based on the complete alignment of their nucleotide sequences, using the TN + G model. Tree construction, visualization and colouring was performed as described for the integrase sequences.

### Bacterial strain and growth conditions

Bacteria were maintained as a stock in LB broth supplemented with glycerol (20% v/v), or in the CRYOBANK Bead System, at −80 °C. When required, overnight cultures of *Klebsiella pneumoniae* ST258 strain KP35, enteropathogenic *Escherichia coli* O127:H6 strain E2348/69, *Salmonella* ser. Enteritidis phagotype 4 strain P125109 (NCTC 13349) or the *Salmonella* ser. Enteritidis *ΔSEN1970:FRT ΔSEN1998:FRT* mutant strain were prepared inoculating 3 mL of LB broth with an aliquot/bead of the stock culture and incubated at 37 °C and 120 rpm overnight.

### Assessment of ICEKp258.2 and IE3 excision

Island excision was assessed under three conditions (37 °C/pH 7.0; 23 °C/pH 7.0 and 37 °C/pH 5.4). Three tubes containing 3 mL of LB broth (yeast extract 5 g/L, tryptone 10 g/L and NaCl 10 g/L) were inoculated with 20 μL of an overnight culture of each strain and incubated for 16 h in a shaking incubator. One milliliter of each culture was used for extraction of genomic DNA using the phenol-chloroform technique^62^. Detection of GI excision and formation of the circular episomal element were assessed by nested PCR using primers listed in Table S4 (Supplementary File S1). A first round of PCR was carried out with genomic DNA at a final concentration of approximately 0.4 ng/μL, 0.5 μM of each primer pair (IDT), 0.2 mM dNTP Mix, 1.5 mM MgCl_2_ and 1U of *Taq* DNA Polymerase (Invitrogen™) in a reaction volume of 25 μL. A second round PCR was performed with Platinum™ *Pfx* DNA Polymerase (Invitrogen™) for *attB* and *attP* sequences, using 2 μL of the product of the first round PCR as template, and nested primers, following manufacturer’s instructions. PCR products were visualized under UV light after electrophoresis in Tris-acetate-EDTA buffer at 90 V using 1% agarose gels precast with SafeView. To confirm that the amplicons obtained during the nested PCR correspond to the expected *attB* and *attP* sequences, we purified the obtained fragments from agarose gel using the Wizard® SV Gel and PCR Clean-Up System kit (Promega) following manufacturer’s instructions. The purified PCR products were sequenced by Macrogen Inc. and raw data obtained was analysed using Vector® NTI v11.0.

### Assessing the effect of ROD21 excision on gene expression

Overnight cultures of wildtype *Salmonella* ser. Enteritidis PT4 strain P125109 and the *ΔSEN1970:FRT ΔSEN1998:FRT* isogenic strain (lacking the integrase and putative RDF coding sequences), were inoculated in LB broth at pH 7.0 until OD_600_ = 0.6 was reached, and samples of 1 mL were taken and centrifuged at 8,000 rpm for 6 min. Supernatant was discarded and the bacterial pellet was resuspended in TRIzol^TM^ reagent (Invitrogen^TM^) and stored at −80 °C. Two independent experiments performed in duplicate were carried out.

RNA and DNA extraction was carried out as described by the manufacturer with some modifications. Briefly, after DNA precipitation and wash, 300 µL of TE buffer and 300 µL phenol:chloroform:isoamyl alcohol (25:24:1) (Winkler) was added and vigorously shaken, the mix was then centrifuged at 14,800 rpm for 15 min at 4 °C and the aqueous phase (100 µL) was recovered. DNA was precipitated by adding 60 µL of propan-2-ol and 10 µL of 3 M sodium acetate followed by a 30 min incubation at −20 °C and centrifugation at 14,800 rpm for 15 min at 4 °C. The DNA pellet was washed with 75% ice-cold ethanol and resuspended in nuclease-free water. Reverse transcription was performed with iScript^TM^ cDNA Synthesis Kit (BioRad) according to the manufacturer’s instructions.

Quantitative real-time PCR (qPCR) was performed for the quantification of ROD21 excision, using TaqMan^TM^ probes and TaqMan^TM^ Fast Advanced Master Mix (Applied Biosystems^TM^) following the manufacturer’s instructions for a 20 µL reaction mixture. Standard curves for *attB*-1 and *rpoD* were used for quantification of ROD21 excision and *rpoD* expression using serial one-tenth dilutions of genomic DNA from *Salmonella* ser. Typhimurium strain 14028s, which harbors only one copy of *attB-1* and *rpoD*. Quantitative real-time PCRs (RT-qPCRs) were carried out using specific primers and TaqMan^TM^ MGB probes for genes *SEN1975*, *SEN1980* and *SEN1993* inside ROD21 (Table S4 in Supplementary File S1). A StepOnePlus^TM^ thermocycler was used, employing the cycling conditions established for TaqMan^TM^ Fast reagent. The expression of the target gene was normalized by the housekeeping gene *rpoD* and abundance of each target mRNA was determined by the comparative method (2^−ΔΔCt^).

### Data availability

All data generated and analysed during this study are included in this published article and its Supplementary Information files.

## Electronic supplementary material

Supplementary Information

Dataset 1
