# Supplementary material for: Comparative and phylogenetic analysis of a novel family of Enterobacteriaceae-associated genomic islands that share a conserved excision/integration module
Source: Sci Rep. 2018 Jul 6;8:10292. doi: 10.1038/s41598-018-28537-0 (PMC6035254; doi:10.1038/s41598-018-28537-0)
Supplement: Supplementary file 1 — Supplementary Information [file 41598_2018_28537_MOESM1_ESM.pdf]

Supplementary Information

Comparative and phylogenetic analysis of a novel family of *Enterobacteriaceae*-associated genomic islands that share a conserved excision/integration module

Alejandro Piña-Iturbe[1], Diego Ulloa-Allendes[1], Catalina Pardo-Roa[1], Irenice Coronado-Arrázola[1], Francisco Salazar-Echegarai[1], Bianca Sclavi[2], Pablo A. González[1], Susan M. Bueno[1]*

[1]Millennium Institute on Immunology and Immunotherapy, Departamento de Genética Molecular y Microbiología, Facultad de Ciencias Biológicas, Pontificia Universidad Católica de Chile, Santiago, Chile.

[2]Laboratoire de Biologie et Pharmacologie Appliquée, Centre National de la Recherche Scientifique UMR 8113, École Normale Supérieure Paris-Saclay, Cachan, France.

*sbueno@bio.puc.cl

Supplementary File S1. *Enterobacteriaceae*-associated ROD21-like genomic islands, nested PCR and qPCR. Location, length, and host genomes of EARL islands (**Table S1**). Location and length of genes encoding H-NS homologues and Tcps, island G+C content and distances from *oriC* (**Table S2**). Primers and probes used for quantification of ROD21 excision and gene expression (**Table S3**). Primers used for detection of island excision (**Table S4**). Sequence and location of the left and right attatchment sites of EARL islands, considering the orientation of the Asn-tRNA (**Table S5**). <u>This information is provided in a separate .xlsx file.</u>

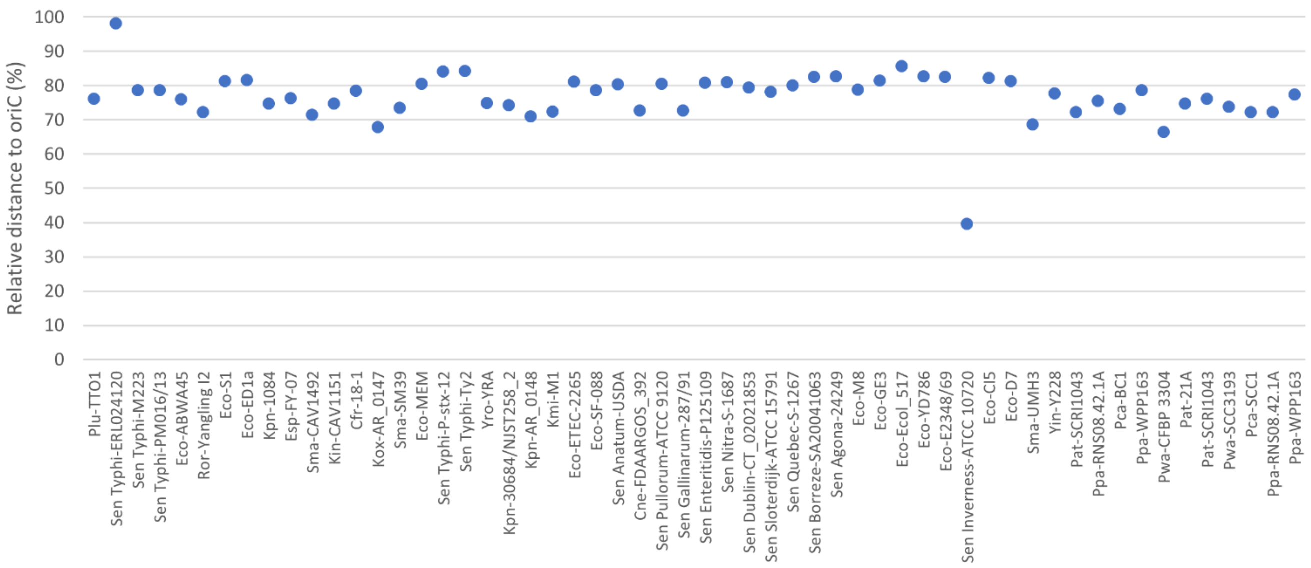

Supplementary Figure S1. EARL GIs are inserted far from the chromosomal replication origin (*oriC*). Relative distance of the insertion site of each EARL island from the chromosomal *oriC* expressed as a percentage of the maximum possible distance (one half of the chromosome length). The location and length of *oriC* were calculated using Ori-Finder (http://tubic.tju.edu.cn/Ori-Finder/)[1]. 0% represents the midpoint of *oriC* and 100% represent the farthest location in the chromosome.

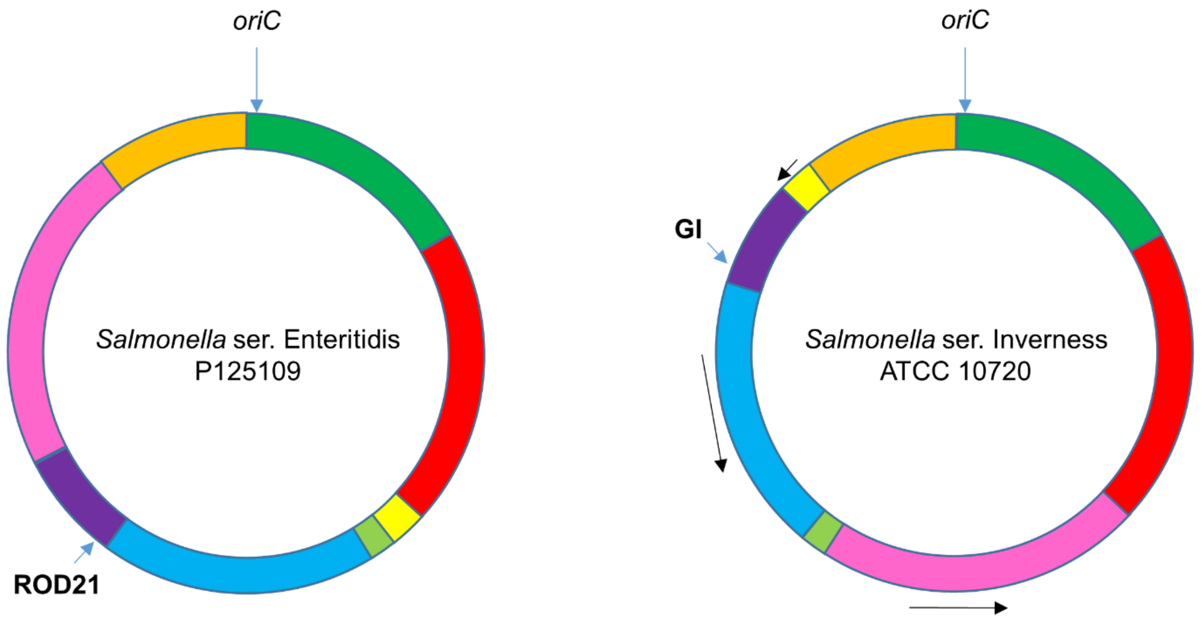

Supplementary Figure S2. The EARL island from *Salmonella* ser. Inverness ATCC 10720 is located near the oriC as the result of a chromosomal rearrangement. Circular representation of Mauve alignment of the chromosomes from *Salmonella* ser. Inverness ATCC 10720 and ser. Enteritidis P125109. The colored regions corresponds to the blocks of homology identified using Mauve v2.4.0[2] and black arrows indicate the regions found in different orientation. ROD21 and GI indicate the approximate location of the EARL GIs found in each chromosome. The origin of replication identified with Ori-Finder is also indicated.

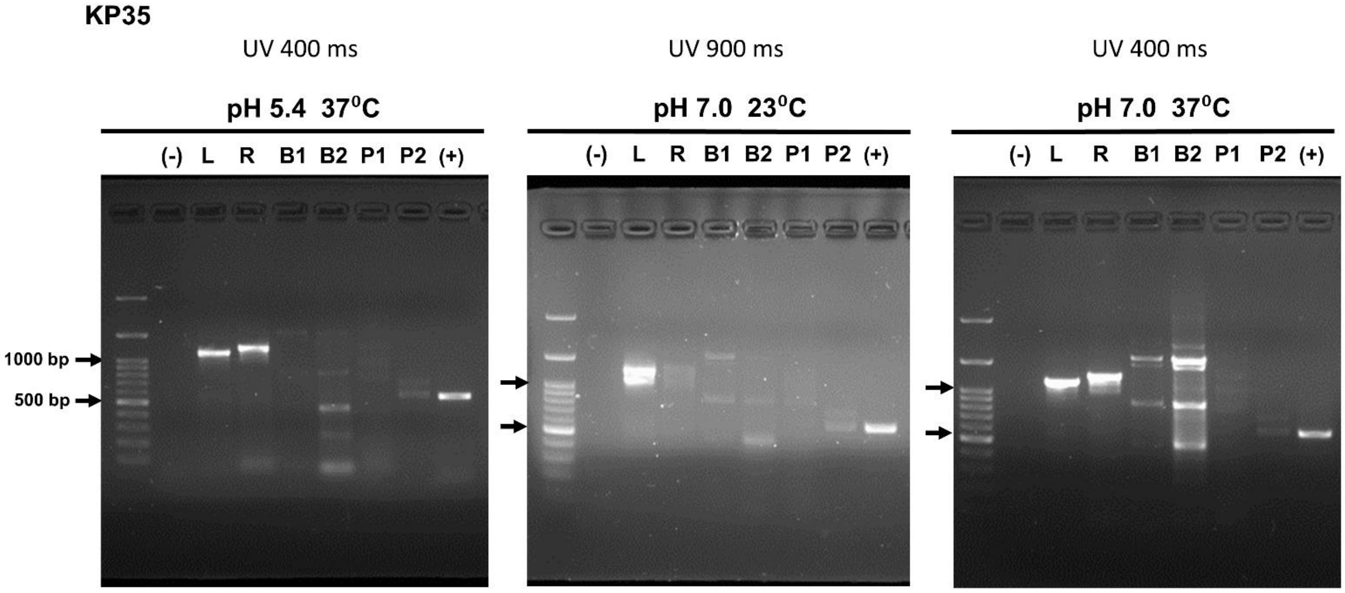

Supplementary Figure S3. Detection of ICEKp258.2 excision in different pH and temperature. Agarose gels showing amplification products (expected sizes of PCR products are: L: 1123 bp, R: 1220 bp, B1: 1029 bp, B2: 519 bp, P1:1340 bp, P2: 668 bp) of nested PCR reactions using genomic DNA obtained from *K. pneumoniae* strain KP35 grown at pH 5.4/37ºC, pH 7.0/23ºC and pH 7.0/37ºC. (-), negative control; L: *attL*, R: *attR*, B1: *attB* from first round of PCR, B2: *attB* from second round of PCR, P1: *attP* from first round of PCR, P2: *attP* from second round of PCR; (+) positive control *rpoD* (577 bp) for the PCR reaction. UV exposure time in milliseconds is indicated above each gel.

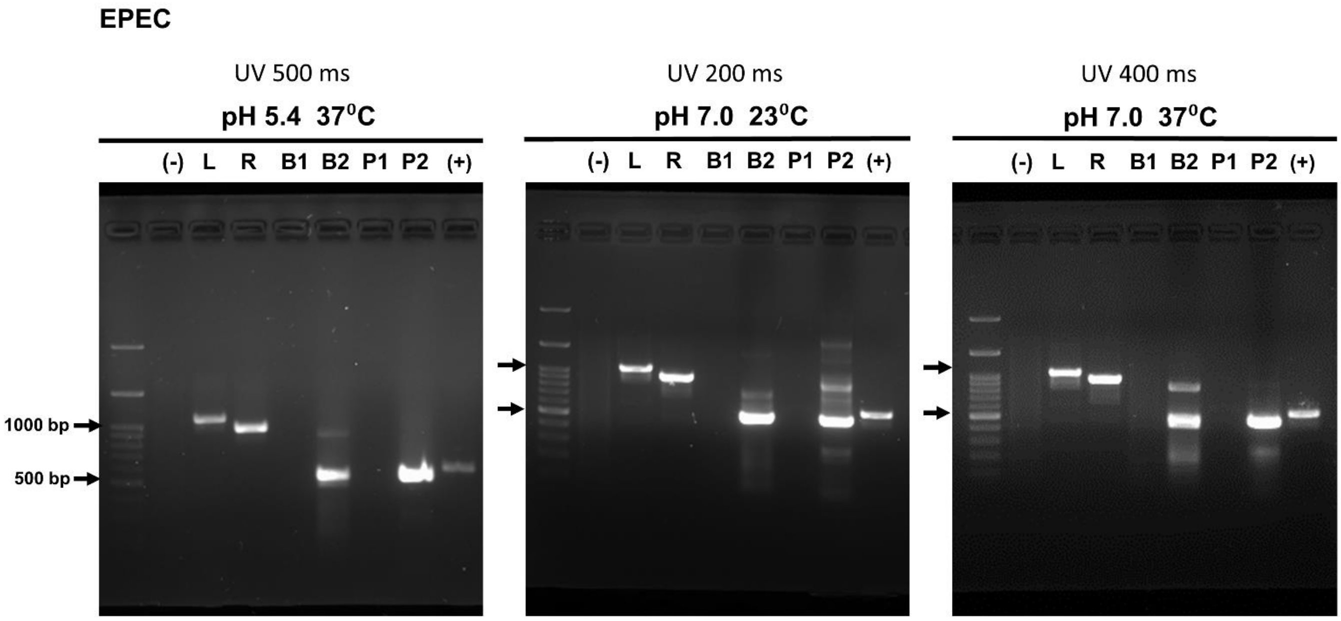

Supplementary Figure S4. Detection of IE3 excision in different pH and temperature. Agarose gel showing amplification products (L: 1057 bp, R: 937 bp, B1: 985 bp, B2: 507 bp, P1: 1009 bp, P2: 502 bp) of nested PCR reactions using genomic DNA obtained from enteropathogenic *E. coli* O127:H6 strain E2348/69 grown at pH 5.4/37ºC, pH 7.0/23ºC and pH 7.0/37ºC. (-), negative control; L: *attL*, R: *attR*, B1: *attB* from first round of PCR, B2: *attB* from second round of PCR, P1: *attP* from first round of PCR, P2: *attP* from second round of PCR; (+) positive control *rpoD* (577 bp) for the PCR reaction. UV exposure time in milliseconds is indicated above each gel.

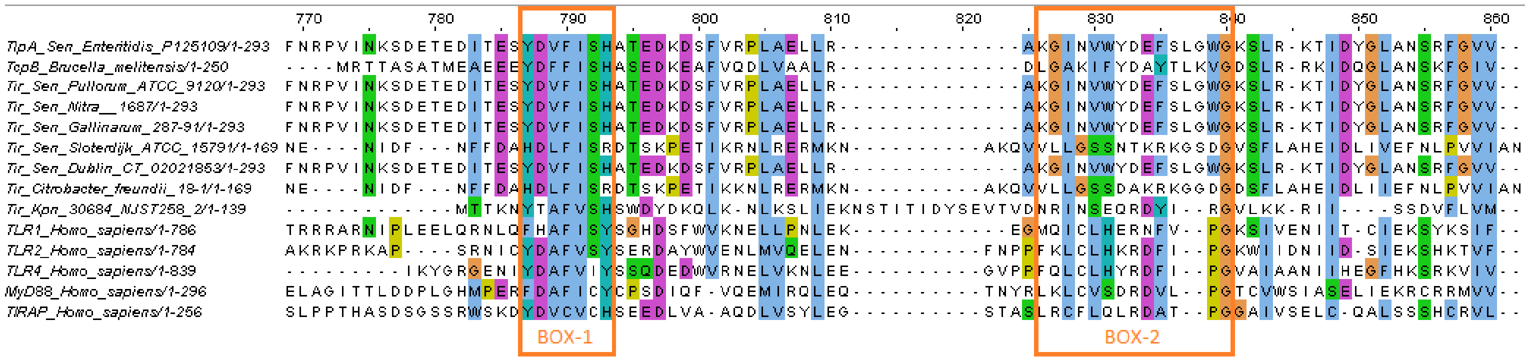

Supplementary Figure S5. Multiple sequence alignment (MSA) of the putative TIR-domain containing proteins (Tcps) encoded in EARL islands (Tir), characterized bacterial Tcps (TlpA and TcpB), and human TIR proteins of the Toll-like receptor (TLR) signaling pathway (TLR1, TLR2, TLR4, MyD88 and TIRAP. The conserved Boxes 1 and 2 of the TIR domain are indicated with orange rectangles. MSA were carried out with Clustal Omega (https://www.ebi.ac.uk/Tools/msa/clustalo/).

References

1. Gao, F. & Zhang, C.-T. Ori-Finder: A web-based system for finding *oriC*s in unannotated bacterial genomes. *BMC Bioinformatics* **9,** 79 (2008).

2. Darling, A. E., Mau, B. & Perna, N. T. progressiveMauve: Multiple Genome Alignment with Gene Gain, Loss and Rearrangement. *PLoS One* **5,** e11147 (2010).

Sequence *attL*+82nt used as query for BLASTn search of EARL islands.

```
> AM933172.1:2061160-2061444 Salmonella ser. Enteritidis P125109

GTTCGAGTCCAGTCAGAGGAGCCAAATTAAGGAAAGCAGACGTTCACTGACGTCTGCTTTCTGCATTTAT
ATCAACTGGTTATTCCCTTCTTCAGGTTCACTCTCGTTCACTAAAAACCACTCGAAGCCATACCCTTTTG
CTGGTAAAAATGCTGGTAAAGCTGGTTCGATTTGTGTTTTACCAGCACGCGGAGGGAACCGTCATGTCAC
TTACTGATACCAAAGTAAAAAATACCAGACCATCGGAAAAGGCCGTCAAGCTCACTGACGGGTTTGGCCT
CTATC
```

Integrases used for construction of island phylogeny. Genome accession number, integrase-coding sequence location and strain.

```
>BX571865.1:c96668-95405 Photorhabdus luminescens subsp. laumondii
TTO1
ATGTCACTGACTGATATTAAAGCAAAAAATGCAAAACCCCTCGAGAAGGAATACAAGTTAACTGATGGCT
TTGGTATGTTCCTTCGCGTTACCCCGAAGGGTTCCAAATACTGGCAAATGGCTTACCGCTTCGAAGGGAA
GCAAAAAATCCTCTCTATAGGTGTTTACCCTGCTGTTTCACTTGCTGATGCAAGACAACGCCGTGATGAG
GCCAGAAGGCTTCTTGCTCAGGGTATTGACCCTACAGGCAGAGGTTAAAGAGCTAAAGGCCAAGCGTGAT
AAGACACGTTGCTTCGCTATGGTGGCTAAGGCTTGGTTTGCCACAAAAACAAAATGGTCAAAAGATTATG
GTGATTCCGTATGGAAGCGCCTTGAAACCTATGTCTTCCCGGCGATTGGAGACAAAGATGTTGCCGAGCT
GGATACAGGCGATCTGCTGGTTCCTGTGAAAAAGGTTGAGGCACTTGGTTATCTTGAAGTTGCCATGCGC
ATTCAACAATACATTACAGCGATCCTGCGTCATGCCGTACAGCAAAAACTGATACTCCATAACCCCGCCT
ATGATATGGAAGGTGCTGTTCAGAAACCACAGACTGAACACCGCCCGGCACTGGAGCTGGAAGAAATCCC
TCAACTACTGAAAAAAATTGCCGAATACAGAGGTCGCAGGTTAACCATACTGGCTATTCAGCTCAATCTG
ATGATTTTCATTCGTTCCAGTGAACTTCGTTTCGCCCGTTGGTCTGAAATTGATTTCAAAAGTAAGTTGT
GGGTGATCCCCGAACAGCGGGAAGCGATTGAAAACGTCAAACATTCCACTCGTGGGGCCAAAATGAAGCG
TAAGCATTTCGTTCCCTTGTGTAAGCAAGCCATGAGGATACTGAAAGAGATCCGACAACTGACTTATAAA
GAAGGCCATGATGATGGATTAATCTTTACTGGCTGTTATGACTCGTTTAAGCCCATGAGCGAAAACACCA
TCAACAAAGCCCTTCGCAATATGGGCTATGACACGAAGCAGGACATCTGTGGACACGGTTTTCGCACGCT
GGCCTGTAGTGCCTTAATTGAGTCAGGTTTGTGGTCAGAAGACGCTGTGGAGCTTCAAATGAGCCATAAG
GAAAGCAACAGCGTCCGTGCTGCTTATACCCACAAGGCAAAACACCTCGAACAGCGCCGCCTGATGCTCC
AATGGTGGGCTGATTTCCTTGATGCAAACCATAACGGTATGGTCAGGCCATTTGAGTTTACTCAAAGAAG
ATAG

>LT906494.1:c296327-295053 Salmonella enterica subsp. enterica serovar
Typhi strain ERL024120
ATGTCACTGACTGATATTAAAGCAAAAAATGCAAAACCCCTTGAGAAGGAATACAAGCTTACTGATGGCT
TTGGTATGTTCCTTCGTGTTACCCCTAAAGGTTCGAAATACTGGCAAATGGCTTACCGCTTCGAAGGGAA
ACAAAAACTCTTCTCTATTGGTGTTTACCCTGCAGTTTCTCTTTCTGACGCAAGACAACGCCGTGACGAG
GCCAGAAGGCTTCTGGCTCAGGGTATTGACCCTAATGCAAAGAAACAGGCAGAGGTTAAAGAGCTTAAAG
CCAAACGTGATAATACACGCACCTTCAGAACAGTAGCCAAAGCGTGGTTCTCCACGAAAACAAAATGGTC
TGATGATTATGGTGATGCCGTATGGAAGCGCCTTGAAACTTATGCCTTCCCGGTAATCGGTGACAAAGAT
GTTGCCGAACTCGATACGGGTGATCTGCTGGTTCCGGTGAAAAAAGTTGAGGCTCTTGGTTATCTTGAAG
TTGCCATGCGCATTCAACAATACATTACGGCAATCCTGCGTCATGCTGTCCAGCAAAAGCTGATACGCCA
TAACCCAGCCTATGATATGGAAGGTGCAGTTCAGAAACCACAAACTGAACACCGCCCTGCACTTGAGCTG
GAAGAAATACCCCAGCTACTGAACAAAATTGCCGAATACAAAGGCCGCAGGTTAACCATACTGGCAATAC
AGCTCAATCTGATGATTTTCATTCGTTCCAGTGAGCTTCGTTTCGCTCGCTGGTCTGAAATTGATTTCAA
AAGTAAGTTATGGGTGATACCCGAACAGCGTGAAGCAATTGAAAACGTCAAACATTCGACTCGTGGTGCC
AAAATGAAGCGTAAGCACTTCGTTCCCCTCTGTAAGCAAGCTATAAGGATACTAAAAGAGATCCGACAAC
TGACTTATGAAGAAGGCCATGATGATGGATTAATCTTCACTGGCTGTTATGACTCGTTTAAACCCATGAG
TGAAAACACCATCAACAAAGCCCTGCGTAATATGGGATATAACACGAAGCAGGACATCTGTGGACACGGT
TTCCGCACTCTGGCCTGTAGTGCCTTAATTGAGTCCGGGCTATGGTCAGAAGACGCTGTGGAGCTTCAAA
TGAGCCATAAGGAAAGTAACAGCGTCCGTGCTGCTTATACCCACAAGGCAAAACATCTTGAGCAACGTCG
CCTGATGCTCCAATGGTGGGCTGACTACCTTGATGCAAGCAGAAACGGTATGGTAAGGCCGTTTGAGTTT
GCTACAAATAAATAA

>CP003278.1:c976446-975172 Salmonella enterica subsp. enterica serovar
Typhi str. P-stx-12
ATGTCACTGACTGATATTAAAGCAAAAAATGCAAAACCCCTTGAGAAGGAATACAAGCTTACTGATGGCT
TTGGTATGTTCCTTCGTGTTACCCCTAAAGGTTCGAAATACTGGCAAATGGCTTACCGCTTCGAAGGGAA
ACAAAAACTCTTCTCTATTGGTGTTTACCCTGCTGTTTCTCTTTCTGACGCAAGACAGCGCCGTGATGAG
GCCAGAAGGCTTCTTGCTCAGGGTATTGACCCTAATGCAAAGAAACAGGCAGAGGTTAAAGAGCTTAAAG
CCAAACGTGATAATACACGCTCCTTCAGAACAGTAGCCAAAGCGTGGTTCTCCACGAAAACAAAATGGTC
TGATGATTATGGTGATGCCGTATGGAAGCGCCTTGAAACTTATGTCTTCCCGGTAATCGGTGACAAAGAT
GTTGCCGAACTGGATACGGGTGATCTGCTGGTTCCGGTGAAAAAAGTTGAGGCTCTTGGTTATCTTGAAG
```

TTGCCATGCGCATTCAACAATACATTACGGCAATCCTGCGTCATGCCGTCCAGCAAAAGCTGATACGCCA
TAACCCAGCCTATGATATGGAAGGTGCAGTTCAGAAACCACAAACTGAACACCGCCCTGCACTTGAGCTG
GAAGAAATACCCCAGTTACTGAACAAAATTGCCGAATACAAAGGCCGCAGGTTAACCATACTGGCAATAC
AGCTCAATCTGATGATTTTCATTCGTTCCAGTGAGCTGCGTTTCGCTCGCTGGTCAGAAATTGATTTCAA
AAGTAAGTTATGGGTGATACCCGAACAGCGTGAAGCGATTGAAAACGTCAAACATTCAACTCGTGGGGCT
AAAATGAAGCGTAAGCACTTAGTTCCCCTTTGTAAGCAGGCCATGAAGATACTCAAAGAGATCCGACAAC
TGACTTATGAAGAAGGTCATGATGACGGATTAATCTTTACTGGTTGTTATGACTCGTTTAAACCCATGAG
CGAAAACACCATCAACAAAGCGCTTCGCAATATGGGCTATGACACAAAGCAGGACATCTGTGGACACGGT
TTCCGCACTCTGGCCTGTAGTGCCTTAATTGAGTCCGGGCTATGGTCAGAAGACGCTGTAGAGCTTCAAA
TGAGCCATAAGGAAAGCAATAGCGTCCGTGCTGCTTATACCCACAAGGCAAAACACCTTGACCAGCGCCG
CCTGATGCTCCAGTGGTGGGCTGATTTCCTTGATGCTAACAGCAACGATATGGTCAGGCCGTTTGAGTTT
GATTCAAATAAATAA

>CP008841.1:4827330-4828610 Klebsiella michiganensis strain M1
ATGTCACTGACTGATATTAAAGCAAAAAATGCAAAACCCCTTGAGAAGGAATACAAGCTAACTGATGGCT
TTGGTATGTTCCTTCGTGTTACCCCTAAAGGTTCGAAATACTGGCAAATGGCCTACCGTTTCGAAGGGAA
GCAAAAACTCTTCTCTATAGGTGTCTACCCTGCGGTTTCTCTTTCTGACGCAAGACAGCGACGCGATGAG
GCCAAAAGGCTTCTTGCTCAGGGCATTGACCCTAATGCAAAGAAACAGGCCGAGGTTAAGGAGCTTAAAG
CTAAACGTGATAATACACGCTCCTTCAGAACAGTAGCCAAAGCGTGGTTCGCCACGAAAACGAAATGGTC
TGATGATTATGGTGATTCCGTATGGAAACGCCTTGAAACTTATGTCTTCCCGGTAATCGGTGACAAAGAT
GTTGCCGAGCTTGATACGGGTGATCTGCTGGTTCCAGTGAAAAAAGTTGAGGCTCTTGGTTATCTTGAAG
TTGCCATGCGCATTCAACAATACATTACGGCTATCCTACGTCATGCCGTACAGCAAAAACTGATACGCCA
TAACCCAGCCTATGATATGGAAGGTGCTGTTCAGAAACCACAGACTGAACACCGCCCTGCACTGGAGCTG
GAAGAAATACCGCAACTACTGAAAAAAATTACCGAATACAAAGGCCGCAGGTTAACCATACTGGCAATAC
AGCTCAATCTGATGATTTTCATTCGTTCCAGTGAGCTGCGTTTCGCTCGCTGGTCTGAAATTGATTTCAA
AAGTAAGTTATGGGTGATACCTGAACAGCGGGAAGCGATTGAAAACGTCAAACATTCCACTCGTGGGGCC
AAAATGAAGCGTAAGCACTTCGTTCCCCTTTGTAAGCAAGCCATGAGGATACTGAAAGAGATCCAACAAC
TGACTTATGAAGAAGGTCATGATGATGGATTAATCTTTACTGGCTGTTATGACTCGTTTAAACCCATGAG
TGAAAACACCATCAACAAAGCCCTGCGTAATATGGGATATAACACGAAGCAGGACATTTGTGGTCATGGC
TTCCGCACACTGGCCTGTAGTGCCTTAATTGAGTCTGGTTTGTGGTCAGAAGACGCTGTAGAGCTTCAGA
TGAGTCACAAGGAAAGCAATAGCGTCCGTGCGGCTTATACCCATAAAGCCAAACACCTTGATCAGCGTCG
TCTGATGTTGCAGTGGTGGGCTGACTTCCTTGATGCCAATCGAAACGATATGGTCAGGCCGTTTGAGTTT
GCTCAAAAAGAAGTCCTATAA

>CP010226.1:1383693-1384967 Escherichia coli strain S1
ATGTCACTGACTGATATTAAAGCAAAAAATGCAAAACCCCTTGAGAAGGAATACAAGCTTACTGATGGCT
TTGGTATGTTCCTTCGTGTTACCCCTAAAGGTTCGAAATACTGGCAAATGGCTTACCGCTTCGAAGGGAA
ACAAAAACTCTTCTCTATTGGTGTTTACCCTGCAGTTTCTCTTTCTGACGCAAGACAACGCCGTGACGAG
GCCAGAAGGCTTCTGGCTCAGGGTATTGACCCTAATGCAAAGAAACAGGCAGAGGTTAAAGAGCTTAAAG
CCAAACGTGATAATACACGCTCCTTCAGAACAGTAGCCAAAGCGTGGTTCTCCACGAAAACAAAATGGTC
TGATGATTATGGTGATGCCGTATGGAAGCGCCTTGAAACTTATGCCTTCCCGGTAATCGGTGACAAAGAT
GTTGCCGAACTGGATACGGGTGATCTGCTGGTTCCGGTGAAAAAAGTTGAGGCTCTTGGTTATCTTGAAG
TTGCCATGCGCATTCAACAATACATTACGGCAATCCTGCGTCATGCCGTCCAGCAAAAGCTGATACGCCA
TAACCCAGCCTATGATATGGAAGGTGCAGTTCAGAAACCACAAACTGAACACCGCCCTGCACTTGAGCTG
GAAGAAATACCCCAGCTACTGAACAAAATTGCCGAATACAAAGGCCGCAGGTTAACCATACTGGCAATAC
AGCTCAATCTGATGATTTTCATTCGTTCCAGTGAGCTTCGTTTCGCTCGCTGGTCTGAAATTGATTTCAA
AAGTAAGTTATGGGTGATACCCGAACAGCGTGAAGCAATTGAAAACGTCAAACATTCGACTCGTGGTGCC
AAAATGAAGCGTAAGCACTTCGTTCCCCTCTGTAAGCAAGCTATAAGGATACTAAAAGAGATCCGACAAC
TGACTTATGAAGAAGGCCATGATGATGGATTAATCTTCACTGGCTGTTATGACTCGTTTAAACCCATGAG
TGAAAACACCATCAACAAAGCCCTGCGTAATATGGGATATAACACGAAGCAGGACATCTGTGGACACGGT
TTCCGCACTCTGGCCTGTAGTGCCTTAATTGAGTCCGGGCTATGGTCAGAAGACGCTGTGGAGCTTCAAA
TGAGCCATAAGGAAAGTAACAGCGTCCGTGCTGCTTATACCCACAAGGCAAAACATCTTGAGCAACGTCG
CCTGATGCTCCAATGGTGGGCTGACTACCTTGATGCAAGCAGAAACGGTATGGTAAGGCCGTTTGAGTTT
GCTACAAATAAATAA

>CP019953.1:2346134-2347402 Escherichia coli M8
ATGTCACTTACTGATACTAAAGTAAAAAATGCCAGACCAGCGGAAAAAGCCGTCAAGCTCACTGATGGAT
TTGGTCTCTACCTTCTTGTGCATCCCAATGGTTCAAAATACTGGCAGTTAGGCTATCGCTTCGAGGGTAA

ACAGAAAGTGTTTTCCATTGGTGTTTACCCTGCGGTTTCTCTTGCTGATGCCAGACAACGACGTGATGAA
GCAAAAAAGCTGCTTGCTCAGGGAATCGATCCTAACGCTAAAAAACAGGCTGATGAAAAAGCTCTGCAGG
AAAAGCGGGATAAAACTCGTTCGTTCCGTGTCGTCGCCAGAAGCTGGTTTGCCACCAAAACAAAATGGTC
AGAAGATTACGCCGATACGGTATGGAAGCGCCTTGAGACCTATGTATTCCCGGATATTGGCGACAGCAAC
GTTTCAGATCTGGATACGGGTGATCTGCTTGTTCCGGTTAAAAAAGCAGAAACGCTTGGCTATCTTGAAA
TTGCCATGCGGATCAAGCAATACATCACTGCCATCCTGCGTCACGCCGTCCAGCAAAAACTGATGCGCCA
TAATCCTGCTTACGATATGGAAGGTGCTGTGCAGAAACCAGAGACTGAGCACCGTCCTGCACTGGAGCTG
GAAGAGATCCCCCTGCTACTTGAACGTATTGATGCCTACAAAGGCCGTGGACTTACCACACTGGCGATTA
AACTCAATCTGTTGATTTTCATTCGTTCCAGTGAACTCCGCTATGCACGATGGTCAGAAATCGACTTCAA
CAGTAAGTTATGGGTGATACCAGAAAAGCGTGAAGCGATTGAGCGCGTCAAATATTCCACGCGTGGTGCA
AAAATGAAACGCCAGCACTTTGTTCCCCTTTGCAGGCAGGCTATTAAGATACTGAAAGAGATCCGCCAGC
TTACCTATGAAGAAGGTAATGAGGCAGGGTTAATTTTCACTGGCTGTTATGACTCATTCAAACCCATGAG
TGAAAACACCATCAATAAAGCGCTGCGTAAGATGGGCTATGATACGAAACAAGACATCTGCGGTCATGGT
TTCCGCACACTGGCATGTAGTGCTCTGATTGAATCAGGGCTATGGTCTGAAGATGCGGTAGAGCTTCAAA
TGAGCCATAAGGAAAGCAACAGCGTTCGTGCTGCCTATACCCATAAGGCCAAACATCTTGAGCAGCGAAG
GTTAATGCTACAGTGGTGGGCAGATTTTCTTGATGCTAACCGGGATGGGATGGTCAGGCCGTTTGAGTTT
ACTCAATAA

>CP019181.1:c926034-924766 Salmonella enterica subsp. enterica serovar Inverness str. ATCC 10720
ATGTCACTTACTGATACTAAAGTAAAAAATGCCAGACCAGCGGAAAAAGCCGTCAAGCTCACTGATGGAT
TTGGTCTCTACCTTCTTGTGCATCCCAATGGTTCAAAATACTGGCAGTTAGGCTATCGCTTCGATGGTAA
ACAGAAAGTGTTTTCCATTGGTGTTTACCCTGCGGTTTCTCTTGCTGATGCCAGACAACGACGTGATGAA
GCAAAAAAGCTGCTTGCGCAGGGAATCGATCCTAACGCTAAAAAACAGGCTGATGAAAAAGCTCTACAGG
AAAAGCGGGATAAAACCCGTTCGTTCCGTGTCGTCGCCAGAAGCTGGTTTGCCACCAAAACAAAATGGTC
AGAAGATTACGCCGATACGGTATGGAAGCGCCTTGAGACCTATGTATTCCCGGACATTGGCGACAGCAAC
GTTTCAGATCTGGATACAGGTGACTTGCTTGTTCCGGTTAAAAAAGCAGAAACGCTTGGCTATCTTGAAA
TTGCCATGCGGATCAAGCAATACATCACTGCCATCCTGCGTCACGCCGTCCAGCAAAAACTTATGCGCCA
TAATCCTGCTTACGATATGGAAGGTGCTGTGCAGAAACCAGAGACTGAGCACCGTCCAGCACTGGAGCTT
GAAGAAATCCCCCTGCTACTTGAACGTATTGATGCCTACAAAGGCCGTGGACTTACCACACTGGCAATTA
AACTCAATCTGTTGATTTTCATTCGTTCCAGTGAACTCCGCTATGCACGATGGTCAGAAATCGACTTCAA
CAGTAAGTTATGGGTGATACCAGAAAAACGTGAAGCGATTGAGCGCGTCAAATATTCCACGCGTGGCGCA
AAAATGAAACGCCAGCACTTTGTTCCCCTCTGCAGGCAGGCTATTAAGATACTGAAAGAGATCCGTCAGC
TTACCTATGAAGAAGGTAATGATGCGGGATTAGTTTTTACCGGCTGTTATGACTCATTCAAACCCATGAG
TGAAAACACTATTAACAAGGCACTGCGTAAGATGGGCTATGACACCACGCAGGACATCTGCGGTCATGGT
TTCCGCACGCTGGCATGTAGTGCTCTGATTGAATCAGGGCTATGGACTGAAGATGCGGTAGAACTCCAGA
TGAGCCATAAGGAAAGCAACAGCGTTCGTGCTGCCTATACCCATAAGGCCAAGCATCTTGAGCAGCGAAG
GTTGATGCTACAGTGGTGGGCAGATTTTCTTGATGCAAACCGGGATGGGATGGTCAGGCCGTTTGAGTTT
ACTCAATAA

>CP018925.1:2796319-2797593 Serratia marcescens strain UMH3
ATGCCACTTACTGATACTAAAGTAAAAAACGCCAAACCCCTTGATAAGGAATACAAGCTGACCGATGGCT
TTGGTATGTTCCTTCGCGTTACCCCTAAAGGTTCCAGATACTGGCAAATGGCTTACCGCTTCGACGGGAA
GCAAAAAATCTTCTCCATAGGTGTCTACCCTGCTGTTTCACTTGCTGATGCAAGACAACGCCGTGATGAG
GCCAGAAGGCTTCTTGCTCAGGGTATTGATCCTAATGCAAAGAAACAGGCCGAAGTTAAAGAGCTAAAAG
CAAAGCGTGATAACACCCGAACATTCAAAGCAGTGACCAAGGCGTGGTTTTCTACCAAAAAGAAATGGTC
TGAAGATTACCAGAACACTGTCTTGACCCGTCTTGAAACCTACATCTTCCCGGATATTGGCAACAGAGAC
GTTACCGGGCTTACCACTGGCGATCTTTTAGTCCCCCTCAAAAAAGTGGAAGCCCTTGGTTATCTGGAAG
TTGCCACACGGGTTAAGCAATATGTCACCTCTATCCTGCGCTATGCCGTCCAACAGCAGCTTATCCGCTA
TAACCCGGCTTACGATCTGGAAGGCTCCATACAGAAACCTGAAACTGAACACCGTCCTGCTCTGGAGCTT
GAAGAGATTCCCTTGCTACTTGAACGTATTGATACCTACAAAGGCCGCAGACTCACCACACTGGCGATTC
AACTTAACCTGCAGGTATTTGTTCGTTCCAGTGAGCTACGCTTCGCCAGATGGTCAGAGATCGATTTCAA
GAGCAAGCTGTGGGTTATCCCCGAACAGCGGGAAGTCATCGAAGGGGTGAAATATTCAGGCCGTGGCACC
AAGATGAAGCGTAAACATTTCATCCCTCTCTGCCGTCAGGCAATCATGTTGCTGGAAGAAATTAAACAGC
TCACCTATGAAGATGGCAATGATGATGGTTTTATCTTCACGGGTAGCTATGACAGCTTTAAACCGATGAG
TGAAAACACCATCAACAAGGCGCTACGTAAGATGGGCTACGATACCCGACAGGACATTTGTGGTCATGGC
TTCCGGACACTTGCCTGTAGTGCCTTAATTGAGTCCGGTTTGTGGTCAGAAGACGCTGTAGAGCTTCAAA
TGAGTCACAAGGAAAGCAATAGTGTCCGCGCCGCTTATACCCACAAAGCCAAACACCTTGACCAGCGCCG

CCTGATGCTCCAATGGTGGGCTGATTATCTGGATGCGAACAGCAACGGTATGGTAAGGCCGTTTGAGTTT
GCTCATAAATTGTAG

>CP019407.1:2009888-2011162 Salmonella enterica subsp. enterica serovar Borreze str. SA20041063
ATGTCACTTACTGATACTAAAGTAAAAAATGCCAGACCAGCGGAAAAAGCCGTCAAGCTCACTGATGGAT
TTGGTCTCTACCTTCTTGTGCATCCCAATGGTTCAAAATACTGGCAGTTAGGCTATCGCTTCGATGGTAA
ACAGAAAGTGTTTTCCATTGGTGTTTACCCTGCAGTTTCTCTTGCTGATGCCAGACAACGACGTGATGAA
GCAAAAAAGCTGCTTGCTCAGGGAATCGATCCTAACGCTAAAAAACAGGCTGATGAAAAAGCTCTGCAGG
AAAAGCGGGATAAAACCCGTTCGTTCCGGGTCGTTGCCAGAAGCTGGTTTGCCACCAAAACAAAATGGTC
TGAAGATTACGCCGATACAGTATGGAAGCGCCTTGAGACCTATGTATTCCCGGATATTGGCGACAGCAAC
GTTTCAGATCTGGATACGGGTGACCTGCTTGTGCCAGTCAAAAAAGCGGAAACACTTGGCTACCTTGAAA
TTGCCATGCGGATTAAGCAATACATCACCGCGATCCTGCGTCATGCTGTCCAGCAAAAGCTTATGCGTCA
TAATCCCGCCTATGATATGGAAGGCGCTGTTCAGAAGCCTGAGACTGAACACCGCCCTGCACTGGAGCTG
GAAGAGATCCCGCTACTGCTTGAACGTATTGATGCCTACAAGGGGCGTGGACTGACTACACTGGCGATTA
AACTCAATCTGCTGATCTTCATTCGTTCCAGCGAACTTCGTTTCGCCCGGTGGTCGGAAATCGACTTCAA
AAGTAAGTTATGGGTGATCCCCGAACAGCGGGAAGCGATTGAGAATGTCAAACATTCCACTCGTGGCGCA
AAAATGAAACGCCAACACTTCGTTCCCCTCTGCCAGCAGGCTATTACGATACTGAAAGAGATCCGTCAGC
TTACCTATGAAGAAGGTAATGATGCGGGATTAATTTTTACGGGCTGTTATGACTCATTCAAGCCGATGAG
TGAAAACACCATCAACAAGGCGCTGCGTAAGATGGGTTATGACACCACACAAGACATCTGTGGACACGGT
TTTCGCACTTTGGCGTGTAGCGCCTTAATTGAGTCTGGTTTATGGTCAGAAGACGCCGTGGAGCTTCAAA
TGAGCCACAAGGAGAGCAACAGCGTTCGTGCTGCCTATACCCACAAGGCCAAGCATCTTGACCAACGCCG
CCTGATGCTTCAGTGGTGGGCTGATTTTCTTGATGCGAATCGGGATGAGATGGTCAGGCCCTTTGAGTTT
GCTCAGAAACAATAA

>CP006876.1:1996007-1997335 Salmonella enterica subsp. enterica serovar Agona str. 24249
ATGCTGGTAAAGCTGGTTCGATTTGCGTTTTACCAGCACGCGGAGGGAACCGTCATGTCACTTACTGATA
CTAAAGTAAAAAATGCCAGACCAGCGGAAAAAGCCGTCAAGCTCACTGATGGATTTGGTCTCTACCTTCT
TGTGCATCCCAATGGTTCAAAATACTGGCAGTTAGGCTATCGCTTCGATGGTAAACAGAAAGTGTTTTCC
ATTGGTGTTTACCCTGCAGTTTCTCTTGCTGATGCCAGACAACGACGTGATGAAGCAAAAAAGCTGCTTG
CTCAGGGAATCGATCCTAACGCTAAAAAACAGGCTGATGAAAAAGCTCTGCAGGAAAAGCGGGATAAAAC
CCGTTCGTTCCGGGTCGTTGCCAGAAGCTGGTTTGCCACCAAAACAAAATGGTCTGAAGATTACGCCGAT
ACAGTATGGAAGCGCCTTGAGACCTATGTATTCCCGGATATTGGCGACAGCAACGTTTCAGATCTGGATA
CGGGTGACCTGCTTGTGCCAGTCAAAAAAGCGGAAACACTTGGCTACCTTGAAATTGCCATGCGGATTAA
GCAATACATCACCGCGATCCTGCGTCATGCTGTCCAGCAAAAGCTTATGCGTCATAATCCCGCCTATGAT
ATGGAAGGCGCTGTTCAGAAGCCTGAGACTGAACACCGCCCTGCACTGGAGCTGGAAGAGATCCCGCTAC
TGCTTGAACGTATTGATGCCTACAAGGGGCGTGGACTGACTACACTGGCGATTAAACTCAATCTGCTGAT
CTTCATTCGTTCCAGCGAACTTCGTTTCGCCCGGTGGTCGGAAATCGACTTCAAAAGTAAGTTATGGGTG
ATCCCCGAACAGCGGGAAGCGATTGAGAATGTCAAACATTCCACTCGTGGCGCAAAAATGAAACGCCAAC
ACTTCGTTCCCCTCTGCCAGCAGGCTATTACGATACTGAAAGAGATCCGTCAGCTTACCTATGAAGAAGG
TAATGATGCGGGATTAATTTTTACGGGCTGTTATGACTCATTCAAGCCGATGAGTGAAAACACCATCAAC
AAGGCGCTGCGTAAGATGGGTTATGACACCACACAAGACATCTGTGGACACGGTTTTCGCACTTTGGCGT
GTAGCGCCTTAATTGAGTCTGGTTTATGGTCAGAAGACGCCGTGGAGCTTCAAATGAGCCACAAGGAGAG
CAACAGCGTTCGTGCTGCCTATACCCACAAGGCCAAGCATCTTGACCAACGCCGCCTGATGCTTCAGTGG
TGGGCTGATTTTCTTGATGCGAATCGGGATGAGATGGTCAGGCCCTTTGAGTTTGCTCAGAAACAATAA
>CP018965.1:c3361590-3360316 Escherichia coli strain Ecol_517
ATGTCACTTACTGATACTAAAGTAAAAAATGCCAGACCAGCGGAAAAAGCCGTCAAGCTCACTGATGGAT
TTGGTCTCTACCTTCTTGTGCATCCCAATGGTTCAAAATACTGGCAGTTAGGCTATCGCTTCGTTGGTAA
ACAGAAAGTGTTTTCCATTGGTGTTTACCCTGCAGTTTCTCTTGCTGATGCCAGACAACGACGTGATGAA
GCAAAAAAGCTGCTTGCTCAGGGAATCGATCCTAACGCTAAAAAACAGGCTGATGAAAAAGCTCTGCAGG
AAAAGCGGGATAAAACCCGTTCGTTCCGTGTCGTCGCCAGAAGCTGGTTTGCCACCAAAACAAAATGGTC
AGAAGATTACGCCGATACGGTATGGAAGCGCCTTGAGACCTATGTATTCCCGGACATTGGTGACAGCAAC
GTTTCAGATCTGGATACGGGTGATCTGCTTGTTCCTGTTAAAAAAGCAGAAACGCTCGGCTATCTTGAAA
TTGCCATGCGGATCAAGCAATACATCACCGCCATCCTGCGTCACGCCGTCCAGCAAAAACTTATGCGCCA
TAATCCTGCTTATGATATGGAAGGTGCAGTGCAGAAACCAGAGACTGAGCACCGTCCCGCACTGGAGCTG
GAAGAGATCCCCCTGCTACTTGAACGTATTGATGCCTACAAAGGCCGTAGACTTACCACACTGGCGATTA
AACTCAATCTGTTGATTTTCATTCGTTCCAATGAACTCCGCTATGCACGATGGTCAGAAATCGACTTCAA

CAGTAAGTTATGGGTGATACCAGAAAAGCGTGAAGCGATTGAGCGCGTCAAATATTCCACGCGTGGAGCA
AAAATGAAACGCCAGCACTTTGTTCCCCTATGCAGGCAAGCTCTTAAGATACTGAAAGAGATCCGTCAGC
TTACCTATGAAGAAGGTAACGAAGCCGGATTAATTTTAACTGGCTGTTATGACTCATTCAAACCCATGAG
TGAAAACACCATCAATAAAGCGCTGCGTAAGATGGGCTATGATACGAAGCAAGACATCTGTGGACACGGC
TTTCGCACACTGGCCTGTAGTGCCTTAATTGAGTCAGGTTTGTGGTCTGAAGATGCGGTAGAACTCCAGA
TGAGCCACAAGGAAAGCAACAGCGTTCGTGCTGCTTATACCCACAAGGCCAAGCATCTTGAGCAGCGAAG
GTTGATGCTACAATGGTGGGCAGATTTTCTTGATGCTAACCGGGATGGGATGGTCAGGCCGTTTGAGTTT
GCTCAGAAACAATAA

>CP012378.1:c1908906-1907632 Escherichia coli strain MEM
ATGTCACTGACTGATATTAAAGCAAAAAATGCAAAACCCCTTGAGAAGGAATACAAGCTGACTGATGGCT
TTGGTATGTTCCTTCGTGTTACCCCTAAAGGTTCGAAATACTGGCAAATGGCCTACCGTTTCGAAGGGAA
GCAAAAACTTTTCTCTATCGGTGTTTACCCTGCTGTTTCTCTTTCTGACGCAAGACAACGCCGTGACGAG
GCCAGAAGGCTTCTGGCTCAGGGTATTGACCCTAATGCAAAGAAACAGGCAGAGGTTAAAGAGCTTAAAG
CCAAACGTGATAATACACGCTCCTTCAGAACAGTAGCCAAAGCGTGGTTCGCCACGAAAACAAAATGGTC
TGATGATTATGGTGATGCCGTATGGAAGCGCCTTGAAACTTATGTCTTCCCGGTAATCGGTGACAAAGAT
GTTGCCGAACTGGATACAGGTGATCTGCTGGTTCCGGTGAAAAAGGTGGAGGCTCTTGGTTATCTTGAAG
TCGCCATGCGCATTCAACAATACATTACGGCAATCCTGCGTCATGCCGTCCAGCAAAAGCTGATACGTCA
TAACCCAGCCTATGATATGGAAGGGGCAGTTCAGAAACCACAAACTGAACACCGCCCTGCACTTGAGCTG
GAAGAAATACCCCAGCTACTGAACAAAATTGCCGAATACAAAGGCCGCAGGTTAACCATACTGGCAATAC
AGCTCAATCTGATGATTTTCATTCGTTCCAGTGAGCTTCGTTTCGCTCGCTGGTCAGAAATTGATTTCAA
AAGTAAGTTATGGGTGATACCTGAACAGCGTGAAGCAATTGAAAACGTCAAACATTCAACTCGTGGGGCT
AAAATGAAGCGTAAGCACTTCGTTCCCCTTTGTAAGCAGGCCATGAAGATACTCAAAGAGATCCGACAAC
TGACTTATGAAGAAGGTCAAGATGATGGGTTAATCTTTACTGGCTGTTATGACTCGTTTAAGCCCATGAG
TGAAAACACCATCAACAAAGCCCTCCGCAATATGGGCTATGACACGAAGCAGGACATCTGTGGACACGGT
TTCCGCACTCTGGCCTGTAGTGCCTTAATTGAGTCCGGGCTATGGTCAGAAGACGCTGTAGAGCTTCAAA
TGAGCCATAAGGAAAGCAACAGCGTCCGTGCTGCTTATACCCACAAGGCAAAACATCTTGAGCAGCGTCG
CCTGATGCTTCAGTGGTGGGCTGATTTCCTTGATGCTAACAGCAACGATATGGTCAGGCCGTTTGAGTTT
GCTTCAAATAAATAA

>CP012376.1:2144844-2146112 Escherichia coli strain GE3
ATGTCACTTACTGATACTAAAGTAAAAAATGCCAGACCAGCGGAAAAAGCCGTCAAGCTCACTGATGGAT
TTGGTCTCTACCTTCTTGTGCATCCCAATGGTTCAAAATACTGGCAGTTAGGCTATCGCTTCGAGGGTAA
ACAGAAAGTGTTTTCCATTGGTGTTTACCCTGCGGTTTCTCTTGCTGATGCCAGACAACGACGTGATGAA
GCAAAAAAGCTGCTTGCTCAGGGAATCGATCCTAACGCTAAAAAACAGGCTGATGAAAAAGCTCTGCAGG
AAAAGCGGGATAAAACTCGTTCGTTCCGTGTCGTCGCCAGAAGCTGGTTTGCCGCCAAAACAAAATGGTC
AGAAGATTACGCCGATACGGTATGGAAGCGCCTTGAGACCTATGTATTCCCGGATATTGGCGACAGCAAC
GTTTCAGATCTGGATACGGGTGATCTGCTTGTTCCGGTTAAAAAAGCAGAAACGCTTGGCTATCTTGAAA
TTGCCATGCGGATCAAGCAATACATCACTGCCATCCTGCGTCACGCCGTCCAGCAAAAACTGATGCGCCA
TAATCCTGCTTACGATATGGAAGGTGCTGTGCAGAAACCAGAGACTGAGCACCGTCCTGCACTGGAGCTG
GAAGAGATCCCCCTGCTACTTGAACGTATTGATGCCTACAAAGGCCGTGGACTTACCACACTGGCGATTA
AACTCAATCTGTTGATTTTCATTCGTTCCAGTGAACTCCGCTATGCACGATGGTCAGAAATCGACTTCAA
CAGTAAGTTATGGGTGATACCAGAAAAGCGTGAAGCGATTGAGCGCGTCAAATATTCCACGCGTGGTGCA
AAAATGAAACGCCAGCACTTTGTTCCCCTTTGCAGGCAGGCTATTAAGATACTGAAAGAGATCCGCCAGC
TTACCTATGAAGAAGGTAATGAGGCAGGGTTAATTTTCACTGGCTGTTATGACTCATTCAAACCCATGAG
TGAAAACACCATCAATAAAGCGCTGCGTAAGATGGGCTATGATACGAAACAAGACATCTGCGGTCATGGT
TTCCGCACACTGGCATGTAGTGCTCTGATTGAATCAGGGCTATGGTCTGAAGATGCGGTAGAGCTTCAAA
TGAGCCATAAGGAAAGCAACAGCGTTCGTGCTGCCTATACCCATAAGGCCAAACATCTTGAGCAGCGAAG
GTTAATGCTACAGTGGTGGGCAGATTTTCTTGATGCTAACCGGGATGGGATGGTCAGGCCGTTTGAGTTT
ACTCAATAA

>AE014613.1:c978771-977497 Salmonella enterica subsp. enterica serovar Typhi Ty2
ATGTCACTGACTGATATTAAAGCAAAAAATGCAAAACCCCTTGAGAAGGAATACAAGCTTACTGATGGCT
TTGGTATGTTCCTTCGTGTTACCCCTAAAGGTTCGAAATACTGGCAAATGGCTTACCGCTTCGAAGGGAA
ACAAAAACTCTTCTCTATTGGTGTTTACCCTGCTGTTTCTCTTTCTGACGCAAGACAGCGCCGTGATGAG
GCCAGAAGGCTTCTTGCTCAGGGTATTGACCCTAATGCAAAGAAACAGGCAGAGGTTAAAGAGCTTAAAG
CCAAACGTGATAATACACGCTCCTTCAGAACAGTAGCCAAAGCGTGGTTCTCCACGAAAACAAAATGGTC

TGATGATTATGGTGATGCCGTATGGAAGCGCCTTGAAACTTATGTCTTCCCGGTAATCGGTGACAAAGAT
GTTGCCGAACTGGATACGGGTGATCTGCTGGTTCCGGTGAAAAAAGTTGAGGCTCTTGGTTATCTTGAAG
TTGCCATGCGCATTCAACAATACATTACGGCAATCCTGCGTCATGCCGTCCAGCAAAAGCTGATACGCCA
TAACCCAGCCTATGATATGGAAGGTGCAGTTCAGAAACCACAAACTGAACACCGCCCTGCACTTGAGCTG
GAAGAAATACCCCAGTTACTGAACAAAATTGCCGAATACAAAGGCCGCAGGTTAACCATACTGGCAATAC
AGCTCAATCTGATGATTTTCATTCGTTCCAGTGAGCTGCGTTTCGCTCGCTGGTCAGAAATTGATTTCAA
AAGTAAGTTATGGGTGATACCCGAACAGCGTGAAGCGATTGAAAACGTCAAACATTCAACTCGTGGGGCT
AAAATGAAGCGTAAGCACTTAGTTCCCCTTTGTAAGCAGGCCATGAAGATACTCAAAGAGATCCGACAAC
TGACTTATGAAGAAGGTCATGATGACGGATTAATCTTTACTGGTTGTTATGACTCGTTTAAACCCATGAG
CGAAAACACCATCAACAAAGCGCTTCGCAATATGGGCTATGACACAAAGCAGGACATCTGTGGACACGGT
TTCCGCACTCTGGCCTGTAGTGCCTTAATTGAGTCCGGGCTATGGTCAGAAGACGCTGTAGAGCTTCAAA
TGAGCCATAAGGAAAGCAATAGCGTCCGTGCTGCTTATACCCACAAGGCAAAACACCTTGACCAGCGCCG
CCTGATGCTCCAGTGGTGGGCTGATTTCCTTGATGCTAACAGCAACGATATGGTCAGGCCGTTTGAGTTT
GATTCAAATAAATAA

>CP012349.1:c1849487-1848213 Salmonella enterica subsp. enterica
serovar Sloterdijk str. ATCC 15791
ATGTCACTTACTGATACCAAAGTAAAAAATACCAGACCATCGGAAAAGGCCGTCAAGCTCACTGACGGGT
TTGGCCTCTATCTGCTGGTGCATCCTAACGGTTCAAAATACTGGCAGTTAGGCTATCGCTTTGATGGCAA
ACAGAAGGTGTTTTCCATTGGGGTTTACCCTGCGGTTTCACTTGCCGATGCCAGACAACGCCGGGACGAG
GCCAAAAGGCTGCTGGCTCAGGGGATTGACCCGAACGCTAAAAAACAGGCTGATGAAAAAGTCCTTCAGG
AGAAGCGGGATAAAACCCGTTCGTTCCGTGTCGTCGCCAAAAGCTGGTTTGCCACCAAAACAAAATGGTC
AGAAGATTACGCCGATACTGTCTGGAAGCGCCTTGAAACCTATGTCTTCCCGGATATAGGCGACAGCAAC
GTTTCAGATCTGGATACGGGTGATCTGCTTGTGCCGGTCAAAAAAGCGGAAACACTTGGCTACCTTGAAA
TTGCCATGCGGATTAAGCAATACATCACCGCGATCCTCCGTCATGCTGTCCAGCAAAAGCTTATGCGTCA
TAATCCCGCCTATGATATGGAAGGCGCTGTTCAGAAGCCAGAGACTGAACACCGCCCTGCACTGGAGCTG
GAAGAGATCCCGCTACTGCTTGAACGTATTGATGCCTACAAAGGGCGTGGACTGACTACACTGGCGATTA
AACTCAATCTGCTGATCTTCATTCGTTCCAGTGAACTTCGTTTCGCTCGGTGGTCAGAAATAGACTTCAA
AAGTAAGTTATGGGTCATTCCCGAACAGCGGGAAGCGATTGAGAATGTCAAATACTCCACTCGTGGGGCT
AAAATGAAACGCCAACACTTCGTTCCCCTCTGCCAGCAGGCTGTTACGATACTGAAAGAGATCCGCCAGC
TTACCTATGAAGAAGGTAATGATGCGGGATTAATTTTTACCGGCTGCTATGACTCATTCAAACCCATGAG
TGAAAACACCATCAACAAGGCGCTGCGTAAGATGGGCTATGACACCACACAGGACATCTGCGGTCATGGT
TTCCGCACACTGGCATGTAGCGCCTTAATTGAGTCTGGTTTATGGTCAGAAGATGCTGTAGAGCTTCAGA
TGAGCCACAAGGAAAGCAACAGCGTTCGCGCTGCTTATACCCATAAGGCCAAGCATCTTGACCAACGCCG
CCTGATGCTCCAGTGGTGGGCTGATTTTCTCGATGCGAATAGGGATGGAATGGTCAGGCCGTTTGAGTTT
GCTCAGAAACAATAA

>CP011018.1:c437987-436719 Escherichia coli strain CI5
ATGTCACTGACTGATACTAAAGTAAAAAATGCCAGACCAGCGGAAAAGGCCGTCAAGCTCACTGACGGGT
TTGGCCTCTACCTGCTGGTGCATCCCAACGGTTCGAAATACTGGCAGTTAGGCTATCGCTTCGATGGCAA
ACAGAAGGTGTTTTCCATTGGGGTTTACCCTGCGGTTTCACTTGCTGATGCCAGACAACGCCGGGACGAG
GCTAAGAGGCTGTTAGCTCAGGGAATCGATCCTAACGCTAAAAAACAGGCTGATGAAAAAGCTCTACAGG
AAAAGCGGGATAAAACCCGTTCGTTCCGTGTCGTCGCCAGAAGCTGGTTTGCCACCAAAACAAAATGGTC
AGAAGATTACGCCGATACGGTATGGAAGCGCCTTGAGACCTATGTATTCCCGGACATTGGCGACAGCAAC
GTTTCAGATCTGGATACAGGTGACTTGCTTGTTCCGGTTAAAAAAGCAGAAACGCTTGGCTATCTTGAAA
TTGCCATGCGGATCAAGCAATACATCACTGCCATCCTGCGTCACGCCGTCCAGCAAAAACTTATGCGCCA
TAATCCGGCCTATGATATGGAAGGTGCTGTGCAGAAACCAGAGACTGAGCACCGTCCAGCACTGGAGCTT
GAAGAAATCCCCCTGCTACTTGAACGTATTGATGCCTACAAAGGCCGTGGACTTACCACACTGGCAATTA
AACTCAATCTGTTGATTTTCATTCGTTCCAGTGAACTCCGCTATGCACGATGGTCAGAAATCGACTTCAA
CAGTAAGTTATGGGTGATACCAGAAAAACGTGAAGCGATTGAGCGCGTCAAATATTCCACGCGTGGCGCA
AAAATGAAACGCCAGCACTTTGTTCCCCTCTGCGGGCAGGCTATTAAGATACTGAAAGAGATCCGTCAGC
TTACCTATGAAGAAGGTAATGATGCGGGATTAGTTTTTACCGGCTGTTATGACTCATTCAAACCCATGAG
TGAAAACACTATTAACAAGGCACTGCGTAAGATGGGCTATGACACCACGCAGGACATCTGCGGTCATGGT
TTCCGCACGCTGGCATGTAGTGCTCTGATTGAATCAGGGCTATGGACTGAAGATGCGGTAGAACTCCAGA
TGAGCCATAAGGAAAGCAACAGCGTTCGTGCTGCCTATACCCATAAGGCCAAGCATCTTGAGCAGCGAAG
GTTGATGCTACAGTGGTGGGCAGATTTTCTTGATGCAAACCGGGATGGGATGGTCAGGCCGTTTGAGTTT
ACTCAATAA

>LT904854.1:2870642-2871916 Salmonella enterica subsp. enterica serovar Typhi strain M223
ATGTCACTGACTGATATTAAAGCAAAAAATGCAAAACCCCTTGAGAAGGAATACAAGCTTACTGATGGCT
TTGGTATGTTCCTTCGTGTTACCCCTAAAGGTTCGAAATACTGGCAAATGGCTTACCGCTTCGAAGGGAA
ACAAAAACTCTTCTCTATTGGTGTTTACCCTGCAGTTTCTCTTTCTGACGCAAGACAACGCCGTGACGAG
GCCAGAAGGCTTCTGGCTCAGGGTATTGACCCTAATGCAAAGAAACAGGCAGAGGTTAAAGAGCTTAAAG
CCAAACGTGATAATACACGCACCTTCAGAACAGTAGCCAAAGCGTGGTTCTCCACGAAAACAAAATGGTC
TGATGATTATGGTGATGCCGTATGGAAGCGCCTTGAAACTTATGCCTTCCCGGTAATCGGTGACAAAGAT
GTTGCCGAACTCGATACGGGTGATCTGCTGGTTCCGGTGAAAAAAGTTGAGGCTCTTGGTTATCTTGAAG
TTGCCATGCGCATTCAACAATACATTACGGCAATCCTGCGTCATGCTGTCCAGCAAAAGCTGATACGCCA
TAACCCAGCCTATGATATGGAAGGTGCAGTTCAGAAACCACAAACTGAACACCGCCCTGCACTTGAGCTG
GAAGAAATACCCCAGCTACTGAACAAAATTGCCGAATACAAAGGCCGCAGGTTAACCATACTGGCAATAC
AGCTCAATCTGATGATTTTCATTCGTTCCAGTGAGCTTCGTTTCGCTCGCTGGTCTGAAATTGATTTCAA
AAGTAAGTTATGGGTGATACCCGAACAGCGTGAAGCAATTGAAAACGTCAAACATTCGACTCGTGGTGCC
AAAATGAAGCGTAAGCACTTCGTTCCCCTCTGTAAGCAAGCTATAAGGATACTAAAAGAGATCCGACAAC
TGACTTATGAAGAAGGCCATGATGATGGATTAATCTTCACTGGCTGTTATGACTCGTTTAAACCCATGAG
TGAAAACACCATCAACAAAGCCCTGCGTAATATGGGATATAACACGAAGCAGGACATCTGTGGACACGGT
TTCCGCACTCTGGCCTGTAGTGCCTTAATTGAGTCCGGGCTATGGTCAGAAGACGCTGTGGAGCTTCAAA
TGAGCCATAAGGAAAGTAACAGCGTCCGTGCTGCTTATACCCACAAGGCAAAACATCTTGAGCAACGTCG
CCTGATGCTCCAATGGTGGGCTGACTACCTTGATGCAAGCAGAAACGGTATGGTAAGGCCGTTTGAGTTT
GCTACAAATAAATAA

>CP010150.1:c4276508-4275234 Escherichia coli strain D7
ATGTCACTTACTGATACTAAAGTAAAAAATGCCAGACCAGCGGAAAAAGCCGTCAAGCTCACTGATGGAT
TCGGTCTCTATCTCCTTGTGCATCCCAATGGTTCAAAATACTGGCAGTTAGGCTATCGCTTCGATGGTAA
ACAGAAAGTGTTTTCCATTGGTGTTTACCCTGCGGTTTCTCTTGCTGATGCCAGACAACGACGTGATGAA
GCAAAAAAGCTGCTTGCTCAGGGAATCGATCCTAACGCTAAAAAACAGGCTGATGAAAAAGCTCTGCAGG
AAAAGCGGGATAAAACCCTTTCGTTCCGGGTCGTTGCCAGAAGCTGGTTTGCCACCAAAACAAAATGGTC
AGAAGATTACGCCGATACGGTATGGAAGCGCCTTGAGACCTATGTATTCCCGGACATTGGCGACAGCAAC
GTTTCAGATCTGGATACAGGTGACTTGCTTGTTTCGGTTAAAAAAGCAGAAACGCTTGGCTATCTTGAAA
TTGCCATGCGGATCAAGCAATACATCACTGCCATCCTGCGTCACGCCGTCCAGCAAAAGCTTATGCGCCA
TAATCCTGCTTACGATATGGAAGGTGCTGTGCAGAAACCAGAGACTGAGCACCGTCCTGCACTGGAGCTG
GAAGAGATCCCCCTGTTACTTGAACGTATTGATGCTTACAAAGGCCGTGGACTTACCACACTGGCGATTA
AACTCAATCTGTTGATTTTCATTCGTTCCAGTGAACTCCGCTATGCACGATGGTCAGAAATCGACTTCAA
CAGTAAGTTATGGGTGATACCAGAAAAGCGTGAAGCGATTGAGCGCGTCAAATATTCCACGCGTGGTGCA
AAAATGAAACGCCAGCACTTTGTTCCCCTTTGCAGGCAGGCTATTAAGATACTGAAAGAGATCCGTCAGC
TTACCTATGAAGAAGGTAATGAGACGGGGTTAATTTTTACGGGCTGTTATGACTCGTTCAAACCCATGAG
TGAAAACACCATCAATAAAGCACTACGTAAGATGGGCTATGACACCACGCAGGATATTTGCGGTCATGGT
TTCCGCACACTGGCATGTAGTGCTCTGATTGAATCAGGGCTATGGTCTGAAGATGCTGTAGAGCTTCAGA
TGAGCCATAAGGAAAGCAACAGCGTTCGCGCTGCCTATACCCACAAAGCTAAGCATCTTGACCAACGCCG
CCTGATGCTTCAGTGGTGGGCTGATTTTCTTGATGCGAATCGGAATGGGATGGTCAGGCCGTTTGAGTTT
GCTAATCAAAAGTAA

>CP022019.1:1273301-1274575 Salmonella enterica subsp. enterica serovar Quebec str. S-1267
ATGTCACTTACTGATACCAGAGTAAAAAATGCCAGACCATCGGAAAAGGTCGTCAAGCTCACTGATGGAT
TTGGTCTCTACCTTCTCGTGCATCCCAACGGTTCGAAATACTGGCAGTTAGGCTACCGCTTTAATGGCAA
ACAGAAGGTGTTTTCCATTGGGGTTTACCCTGCGGTTTCTCTTGCTGATGCCAGACAACGACGTGATGAA
GCAAAAAAGCTGCTTGCTCAGGGAATCGATCCTAACGCTAAAAAACAGGCTGATGAAAAAGCTCTGCAGG
AAAAGCGGGATAAAACCCGTTCGTTCCGGGTCGTTGCCAGAAGCTGGTTTGCCACCAAAACAAAATGGTC
TGAAGATTACGCCGATACAGTATGGAAGCGCCTTGAGACCTATGTATTCCCGGATATTGGCGACAGCAAC
GTTTCAGATCTGGATACGGGTGACCTGCTTGTGCCAGTCAAAAAAGCGGAAACGCTTGGCTACCTTGAAA
TTGCCATGCGGATTAAACAATACATCACCGCGATCCTGCGCCATGCCGTCCAGCAAAAGCTTATGCGTCA
TAATCCCGCCTATGATATGGAAGGCGCTGTTCAGAAGCCAGAGACTGAACACCGCCCTGCACTGGAGCTG
GAAGAGATCCCGCTACTGCTTGAACGTATTGATGCCTACAAAGGGCGTGGACTGACTACACTGGCGATTA
AACTCAATCTGCTGATCTTCATTCGTTCCAGCGAACTTCGTTTCGCTCGGTGGTCGGAAATCGACTTCAA
AAGTAAGTTATGGGTGATCCCCGAACAGCGGGAAGCGATTGAAAACGTCAAGCACTCGACTCGTGGGGCT
AAAATGAAACGTCAGCACTTCGTTCCCCTTTGCAGGCAGGCTCTTAAGATACTGAAAGAGATCCGCCAGC

TTACCTATGAAGAAGGTAACGAAGCCGGATTAATTTTTACTGGATGTTATGACTCATTCAAACCCATGAG
TGAAAACACCATAAACAAGGCGCTGCGTAAGATGGGCTATGACACCACACAGGACATCTGCGGTCATGGC
TTCCGCACACTGGCATGTAGCGCCTTAATTGAGTCTGGCCTATGGTCAGAAGACGCTGTGGAGCTTCAAA
TGAGCCATAAGGAAAGTAACAGCGTTCGCGCAGCCTATACCCATAAGGCCAAGCATCTTGACCAACGCCG
CCTGATGCTCCAGTGGTGGGCTGATTTTCTCGATGCGAATAGGGATGGAATGGTCAGGCCGTTTGAGTTT
GCTCAGAAACAATAA

>CP023525.1:c1720180-1718906 Cedecea neteri strain FDAARGOS_392
ATGCCACTTACTGATACTAAAGTAAAAAATGCTAAACCCCTTGAGAAGGAATACAAGCTGACCGATGGCT
TTGGTATGTTCCTTCGCGTTACCCCCAAGGGTTCCAAATACTGGCAAATGGCTTACCGCTTCGAAGGAAA
GCAAAAACTTTTCTCTATTGGTGTTTACCCTGCTGTTTCACTTGCTGATGCAAGACAACGTCGTGATGAG
GCCAGAAGGCTTCTTGCTCAGGGCATTGACCCTAATGCAAAGAAACAGGCAGAGGTTAAAGAACTAAAGG
CCAAGCGTGATAAGACATGCACCTTCACTGTGGTGGCAAAAGCTTGGTTCGCCACAAAAACAAAATGGTC
AGAAGATTACGGTGATTCCGTATGGAAGCGCCTTGAAACCTATGTCTTCCCGGCGATTGGAGATAAAGAT
GTTGCCGAACTGGATACGGGTGACCTGCTGGTTCCGGTGAAAAAGGTTGAGGCACTTGGCTATCTTGAAG
TTGCCATGCGCATTCAACAATACATTACGGCGATCCTCCGTCATGCTGTCCAGCAAAAGCTGATACGCCA
TAACCCTGCCTATGACATGGAAGGTGCTGTTCAGAAACCACAGACTGAACACCGCCCTGCGTTAGAGCTG
GAAGAAATCCCTCAACTACTGAAAAAAATTGCCGAATACAAAGGTCGCAGGTTAACCATACTGGCAATCC
AGCTCAATCTGATGATTTTTATTCGTTCCAGTGAGCTTCGTTTCGCTCGCTGGTCAGAAATTGATTTCAA
AAGTAAGTTATGGGTGATACCCGAACAGCGTGAAGCGATTGAAAACGTCAAACATTCAACTCGTGGGGCA
AAAATGAAGCGTAAGCACTTCGTCCCCCTTTGTAAGCAGGCCATGAAGATACTCAAAGAGATCCGACAAC
TGACTTATGAAGAAGGTCATGATGACGGATTAATCTTTACTGGCTGTTATGACTCGTTTAAGCCCATGAG
CGAAAATACCATCAACAAAGCCCTTCGCAATATGGGCTATGACACGAAGCAGGACATCTGTGGACACGGT
TTCCGCACACTGGCCTGTAGTGCCTTAATTGAGTCAGGTTTGTGGTCAGAAGACGCTGTAGAGCTTCAAA
TGAGTCACAAGGAAAGCAATAGTGTCCGTGCTGCTTACACTCACAAGGCCAAGCATCTTGATCAGCGCCG
CCTGATGCTGCAGTGGTGGGCTGATTATCTTGACGCTAACAGCTATGGCATGATCAGGCCGTTTGAGTTT
GCTCATCACAAGTAA

>FM180568.1:c2172882-2171614 Escherichia coli 0127:H6 strain E2348/69
ATGTCACTTACTGATACTAAAGTAAAAAATGCCAGACCAGCGGAAAAAGCCGTCAAGCTCACTGATGGAT
TTGGTCTCTACCTTCTTTTGCATCCCAATGGTTCAAAATACTGGCAGTTAGGCTATCGCTTCGATGGTAA
ACAGAAAGTGTTTTCCATTGGTGTTTACCCTGCGGTTTCTCTTGCTGATGCCAGACAACGACGTGATGAA
GCAAAAAAGCTGCTTGCTCAGGGAATCGATCCTAACGCTAAAAAACAGGCTGATGAAAAAGCTCTGCAGG
AAAAGCGGGATAAAACCCGTTCGTTCCGTGTCGTCGCCAGAAGCTGGTTTGCCACCAAAACAAAATGGTC
TGAAGATTACGCCGATACGGTATGGAAGCGCCTTGAGACCTATGTATTCCCGGATATTGGTGACAGCAAC
GTTTCAGATCTGGATACGGGTGATCTGCTTGTTCCGGTAAAAAAAGCAGAAACGCTTGGCTATCTTGAAA
TTGCCATGCGGATCAAGCAATACATCACTGCCATCCTGCGTCACGCCGTCCAGCAAAAACTTATGCGCCA
TAATCCTGCTTACGATATGGAAGGTGCTGTGCAGAAACCAGAGACTGAGCACCGTCCCGCACTGGAGCTG
GAAGAGATCCCCCTGCTACTTGAACGTATTGATGCCTACAAAGGCCGTGGACTTACCACACTGGCGATTA
AACTCAATCTGTTGATTTTCATTCGTTCCAGTGAACTCCGCTATGCACGATGGTCAGAAATCGACTTCAA
CAGTAAGTTATGGGTGATACCAGAAAAGCGTGAAGTGATTGAGCGCGTCAAATATTCCACGCGTGGCGCA
AAAATGAAACGCCAGCACTTTGTTCCCCTTTGCAGGCAGGCTCTTAAGATACTGAAAGAGATCCGTCAGC
TTACCTATGAAGAAGGTAATGACGCCGGATTAATTTTTACTGGATGTTATGACTCATTCAAACCCATGAG
TGAAAACACCATCAACAAGGCGCTGCGTAAGATGGGCTATGACACCACGCAGGACATCTGCGGTCATGGT
TTCCGAACACTGGCATGTAGTGCTCTGATTGAATCAGGGCTATGGTCTAAAGATGCGGTAGAACTTCAGA
TGAGCCACAAGGAAAGCAACAGTGTGCGTGCTGCCTATACCCATAAGGCCAAACATCTTGAACAGAGAAG
ATTGATGTTACAGTGGTGGGCAGATTATCTTGATGCAAATCGGGATGGGATGGTCAGGCCGTTTGAGTTT
GCTCAATAA

>CP012091.1:c4785386-4784112 Salmonella enterica subsp. enterica serovar Typhi strain PM016/13
ATGTCACTGACTGATATTAAAGCAAAAAATGCAAAACCCCTTGAGAAGGAATACAAGCTTACTGATGGCT
TTGGTATGTTCCTTCGTGTTACCCCTAAAGGTTCGAAATACTGGCAAATGGCTTACCGCTTCGAAGGGAA
ACAAAAACTCTTCTCTATTGGTGTTTACCCTGCAGTTTCTCTTTCTGACGCAAGACAACGCCGTGACGAG
GCCAGAAGGCTTCTGGCTCAGGGTATTGACCCTAATGCAAAGAAACAGGCAGAGGTTAAAGAGCTTAAAG
CCAAACGTGATAATACACGCACCTTCAGAACAGTAGCCAAAGCGTGGTTCTCCACGAAAACAAAATGGTC
TGATGATTATGGTGATGCCGTATGGAAGCGCCTTGAAACTTATGCCTTCCCGGTAATCGGTGACAAAGAT
GTTGCCGAACTCGATACGGGTGATCTGCTGGTTCCGGTGAAAAAAGTTGAGGCTCTTGGTTATCTTGAAG

TTGCCATGCGCATTCAACAATACATTACGGCAATCCTGCGTCATGCTGTCCAGCAAAAGCTGATACGCCA
TAACCCAGCCTATGATATGGAAGGTGCAGTTCAGAAACCACAAACTGAACACCGCCCTGCACTTGAGCTG
GAAGAAATACCCCAGCTACTGAACAAAATTGCCGAATACAAAGGCCGCAGGTTAACCATACTGGCAATAC
AGCTCAATCTGATGATTTTCATTCGTTCCAGTGAGCTTCGTTTCGCTCGCTGGTCTGAAATTGATTTCAA
AAGTAAGTTATGGGTGATACCCGAACAGCGTGAAGCAATTGAAAACGTCAAACATTCGACTCGTGGTGCC
AAAATGAAGCGTAAGCACTTCGTTCCCCTCTGTAAGCAAGCTATAAGGATACTAAAAGAGATCCGACAAC
TGACTTATGAAGAAGGCCATGATGATGGATTAATCTTCACTGGCTGTTATGACTCGTTTAAACCCATGAG
TGAAAACACCATCAACAAAGCCCTGCGTAATATGGGATATAACACGAAGCAGGACATCTGTGGACACGGT
TTCCGCACTCTGGCCTGTAGTGCCTTAATTGAGTCCGGGCTATGGTCAGAAGACGCTGTGGAGCTTCAAA
TGAGCCATAAGGAAAGTAACAGCGTCCGTGCTGCTTATACCCACAAGGCAAAACATCTTGAGCAACGTCG
CCTGATGCTCCAATGGTGGGCTGACTACCTTGATGCAAGCAGAAACGGTATGGTAAGGCCGTTTGAGTTT
GCTACAAATAAATAA

>CP012347.1:c1856941-1855667 Salmonella enterica subsp. enterica
serovar Pullorum str. ATCC 9120
ATGTCACTTACTGATACCAAAGTAAAAAATACCAGACCATCGGAAAAGGCCGTCAAGCTCACTGACGGGT
TTGGCCTCTATCTGCTGGTGCATCCTAACGGTTCAAAATACTGGCAGTTAGGCTATCGCTTTGATGGCAA
ACAGAAGGTGTTTTCCATTGGGGTTTACCCTGCGGTTTCACTTGCCGATGCCAGACAACGCCGGGACGAG
GCCAAAAGGCTGCTGGCTCAGGGGATTGACCCGAACGCTAAAAAACAGGCTGATGAAAAAGTCCTTCAGG
AGAAGCGGGATAAAACCCGCTCGTTCCGTGTCGTCGCCAAAAGCTGGTTTGCCACCAAAACAAAATGGTC
AGAAGATTACGCCGATACTGTCTGGAAGCGCCTTGAAACCTATGTCTTCCCGGATATAGGCGACAGAAAC
GTTTCAGAACTGGATACGGGTGATCTGCTTGTCCCGGTCAAAAAAGCGGAAACACTCGGCTACCTTGAAA
TTGCCATGCGGATTAAGCAATACATCACCGCGATCCTACGTCATGCCGTCCAGCAAAAGCTTATGCGTCA
TAATCCGGCCTATGATATGGAAGGCGCTGTCCAGAAACCAGAGACGGAACACCGCCCTGCACTGGAGCTG
GAAGAGATCCCGCTACTGCTTGAACGTATTGATGCCTACAAAGGTCGTGGACTGACTACGCTAGCGATTA
AACTCAATCTGCTGATCTTCATTCGTTCCAGCGAACTTCGTTTCGCCCGGTGGTCGGAAATCGACTTCAA
AAGTAAGTTATGGGTGATCCCCGAACAGCGGGAAGCGATTGAAAACGTCAAGCACTCGACTCGTGGGGCT
AAAATGAAACGTCAGCACTTCGTTCCCCTTTGCAGGCAGGCTCTCAAGATACTGAAAGAGATCCGCCAGC
TTACCTATGAAGAAGGCAACGAAGCAGAATTAATTTTCACTGGCTGTTATGATTCATTCAAACCCATGAG
TGAAAACACTATTAACAAGGCGCTACGTAAGATGGGCTATGACACCACACAGGACATCTGCGGTCATGGT
TTCCGCACACTGGCGTGTAGTGCCTTAATTGAGTCTGGTCTATGGTCAGAAGATGCTGTAGAGCTTCAAA
TGAGCCATAAGGAAAGTAACAGCGTTCGCGCAGCCTATACCCATAAGGCCAAGCATCTTGACCAACGCCG
CCTGATGCTCCAGTGGTGGGCTGATTTTCTTGATGAGAATCGGTATGAGATGGTCAGGCCGTTTGAGTTT
GCTCAGAAACAATAA

>AM933173.1:2052521-2053861 Salmonella enterica subsp. enterica
serovar Gallinarum str. 287/91
TTGCTGGTAAAAATGCTGGTAAAGCTGGTTCGATTTGTGTTTTACCAGCACGCGGAGGGAAACGTCATGT
CACTTACTGATACCAAAGTAAAAAATACCAGACCATCGGAAAAGGCCGTCAAGCTCACTGACGGGTTTGG
CCTCTATCTGCTGGTGCATCCTAACGGTTCAAAATACTGGCAGTTAGGCTATCGCTTTGATGGCAAACAG
AAGGTGTTTTCCATTGGGGTTTACCCTGCGGTTTCACTTGCCGATGCCAGACAACGCCGGGACGAGGCCA
AAAGGCTGCTGGCTCAGGGGATTGACCCGAACGCTAAAAAACAGGCTGATGAAAAAGTCCTTCAGGAGAA
GCGGGATAAAACCCGCTCGTTCCGTGTCGTCGCCAAAAGCTGGTTTGCCACCAAAACAAAATGGTCAGAA
GATTACGCCGATACTGTCTGGAAGCGCCTTGAAACCTATGTCTTCCCGGATATAGGCGACAGAAACGTTT
CAGAACTGGATACGGGTGATCTGCTTGTCCCGGTCAAAAAAGCGGAAACACTCGGCTACCTTGAAATTGC
CATGCGGATTAAGCAATACATCACCGCGATCCTACGTCATGCCGTCCAGCAAAAGCTTATGCGTCATAAT
CCGGCCTATGATATGGAAGGCGCTGTCCAGAAACCAGAGACGGAACACCGCCCTGCACTGGAGCTGGAAG
AGATCCCGCTACTGCTTGAACGTATTGATGCCTACAAAGGTCGTGGACTGACTACGCTAGCGATTAAACT
CAATCTGCTGATCTTCATTCGTTCCAGCGAACTTCGTTTCGCCCGGTGGTCGGAAATCGACTTCAAAAGT
AAGTTATGGGTGATCCCCGAACAGCGGGAAGCGATTGAAAACGTCAAGCACTCGACTCGTGGGGCTAAAA
TGAAACGTCAGCACTTCGTTCCCCTTTGCAGGCAGGCTCTCAAGATACTGAAAGAGCTCCGCCAGCTTAC
CTATGAAGAAGGTAACGAAGCAGAATTAATTTTCACTGGCTGTTATGATTCATTCAAACCCATGAGTGAA
AACACTATTAACAAGGCGCTACGTAAGATGGGCTATGACACCACACAGGACATCTGCGGTCATGGTTTCC
GCACACTGGCGTGTAGTGCCTTAATTGAGTCTGGTCTATGGTCAGAAGATGCTGTAGAGCTTCAAATGAG
CCATAAGGAAAGTAACAGCGTTCGCGCAGCCTATACCCATAAGGCCAAGCATCTTGACCAACGCCGCCTG
ATGCTCCAGTGGTGGGCTGATTTTCTTGATGAGAATCGGTATGAGATGGTCAGGCCGTTTGAGTTTGCTC
AGAAACAATAA

>AM933172.1:2061363-2062637 Salmonella enterica subsp. enterica serovar Enteritidis str. P125109
ATGTCACTTACTGATACCAAAGTAAAAAATACCAGACCATCGGAAAAGGCCGTCAAGCTCACTGACGGGT
TTGGCCTCTATCTGCTGGTGCATCCTAACGGTTCAAAATACTGGCAGTTAGGCTATCGCTTTGATGGCAA
ACAGAAGGTGTTTTCCATTGGGGTTTACCCTGCGGTTTCACTTGCCGATGCCAGACAACGCCGGGACGAG
GCCAAAAGGCTGCTGACTCAGGGGATTGACCCGAACGCTAAAAAACAGGCTGATGAAAAAGTCCTTCAGG
AGAAGCGGGATAAAACCCGCTCGTTCCGTGTCGTCGCCAAAAGCTGGTTTGCCACCAAAACAAAATGGTC
AGAAGATTACGCCGATACTGTCTGGAAGCGCCTTGAAACCTATGTCTTCCCGGATATAGGCGACAGAAAC
GTTTCAGAACTGGATACGGGTGATCTGCTTGTCCCGGTCAAAAAAGCGGAAACACTCGGCTACCTTGAAA
TTGCCATGCGGATTAAGCAATACATCACCGCGATCCTACGTCATGCCGTCCAGCAAAAGCTTATGCGTCA
TAATCCGGCCTATGATATGGAAGGCGCTGTCCAGAAACCAGAGACGGAACACCGCCCTGCACTGGAGCTG
GAAGAGATCCCGCTACTGCTTGAACGTATTGATGCCTACAAAGGTCGTGGACTGACTACGCTAGCGATTA
AACTCAATCTGCTGATCTTCATTCGTTCCAGCGAACTTCGTTTCGCCCGGTGGTCGGAAATCGACTTCAA
AAGTAAGTTATGGGTGATCCCCGAACAGCGGGAAGCGATTGAAAACGTCAAGCACTCGACTCGTGGGGCT
AAAATGAAACGTCAGCACTTCGTTCCCCTTTGCAGGCAGGCTCTCAAGATACTGAAAGAGATCCGCCAGC
TTACCTATGAAGAAGGTAACGAAGCAGAATTAATTTTCACTGGCTGTTATGATTCATTCAAACCCATGAG
TGAAAACACTATTAACAAGGCGCTACGTAAGATGGGCTATGACACCACACAGGACATCTGCGGTCATGGT
TTCCGCACACTGGCGTGTAGTGCCTTAATTGAGTCTGGTCTATGGTCAGAAGATGCTGTAGAGCTTCAAA
TGAGCCATAAGGAAAGTAACAGCGTTCGCGCAGCCTATACCCATAAGGCCAAGCATCTTGACCAACGCCG
CCTGATGCTCCAGTGGTGGGCTGATTTTCTTGATGAGAATCGGTATGAGATGGTCAGGCCGTTTGAGTTT
GCTCAGAAACAATAA

>CP001144.1:2188380-2189654 Salmonella enterica subsp. enterica serovar Dublin str. CT_02021853
ATGTCACTTACTGATACCAAAGTAAAAAATACCAGACCATCGGAAAAGGCCGTCAAGCTCACTGACGGGT
TTGGCCTCTATCTGCTGGTGCATCCTAACGGTTCAAAATACTGGCAGTTAGGCTATCGCTTTGATGGCAA
ACAGAAGGTGTTTTCCATTGGGGTTTACCCTGCGGTTTCACTTGCCGATGCCAGACAACGCCGGGACGAG
GCCAAAAGGCTGCTGGCTCAGGGGATTGACCCGAACGCTAAAAAACAGGCTGATGAAAAAGTCCTTCAGG
AGAAGCGGGATAAAACCCGCTCGTTCCGTGTCGTCGCCAAAAGCTGGTTTGCCACCAAAACAAAATGGTC
AGAAGATTACGCCGATACTGTCTGGAAGCGCCTTGAAACCTATGTCTTCCCGGATATAGGCGACAGAAAC
GTTTCAGAACTGGATACGGGTGATCTGCTTGTCCCGGTCAAAAAAGCGGAAACACTCGGCTACCTTGAAA
TTGCCATGCGGATTAAGCAATACATCACCGCGATCCTACGTCATGCCGTCCAGCAAAAGCTTATGCGTCA
TAATCCGGCCTATGATATGGAAGGCGCTGTCCAGAAACCAGAGACGGAACACCGCCCTGCACTGGAGCTG
GAAGAGATCCCGCTACTGCTTGAACGTATTGATGCCTACAAAGGTCGTGGACTGACTACGCTAGCGATTA
AACTCAATCTGCTGATCTTCATTCGTTCCAGCGAACTTCGTTTCGCCCGGTGGTCGGAAATCGACTTCAA
AAGTAAGTTATGGGTGATCCCCGAACAGCGGGAAGCGATTGAAAACGTCAAGCACTCGACTCGTGGGGCT
AAAATGAAACGTCAGCACTTCGTTCCCCTTTGCAGGCAGGCTCTCAAGATACTGAAAGAGATCCGCCAGC
TTACCTATGAAGAAGGTAACGAAGCAGAATTAATTTTCACTGGCTGTTATGATTCATTCAAACCCATGAG
TGAAAACACTATTAACAAGGCGCTACGTAAGATGGGCTATGACACCACACAGGACATCTGCGGTCATGGT
TTCCGCACACTGGCGTGTAGTGCCTTAATTGAGTCTGGTCTATGGTCAGAAGATGCTGTAGAGCTTCAAA
TGAGCCATAAGGAAAGTAACAGCGTTCGCGCAGCCTATCCCCATAAGGCCAAGCATCTTGACCAACGCCG
CCTGATGCTCCAGTGGTGGGCTGATTTTCTTGATGAGAATCGGTATGAGATGGTCAGGCCGTTTGAGTTT
GCTCAGAAACAATAA

>CP019416.1:2061702-2062976 Salmonella enterica subsp. enterica serovar Nitra strain S-1687
ATGTCACTTACTGATACCAAAGTAAAAAATACCAGACCATCGGAAAAGGCCGTCAAGCTCACTGACGGGT
TTGGCCTCTATCTGCTGGTGCATCCTAACGGTTCAAAATACTGGCAGTTAGGCTATCGCTTTGATGGCAA
ACAGAAGGTGTTTTCCATTGGGGTTTACCCTGCGGTTTCACTTGCCGATGCCAGACAACGCCGGGACGAG
GCCAAAAGGCTGCTGACTCAGGGGATTGACCCGAACGCTAAAAAACAGGCTGATGAAAAAGTCCTTCAGG
AGAAGCGGGATAAAACCCGCTCGTTCCGTGTCGTCGCCAAAAGCTGGTTTGCCACCAAAACAAAATGGTC
AGAAGATTACGCCGATACTGTCTGGAAGCGCCTTGAAACCTATGTCTTCCCGGATATAGGCGACAGAAAC
GTTTCAGAACTGGATACGGGTGATCTGCTTGTCCCGGTCAAAAAAGCGGAAACACTCGGCTACCTTGAAA
TTGCCATGCGGATTAAGCAATACATCACCGCGATCCTACGTCATGCCGTCCAGCAAAAGCTTATGCGTCA
TAATCCGGCCTATGATATGGAAGGCGCTGTCCAGAAACCAGAGACGGAACACCGCCCTGCACTGGAGCTG
GAAGAGATCCCGCTACTGCTTGAACGTATTGATGCCTACAAAGGTCGTGGACTGACTACGCTAGCGATTA
AACTCAATCTGCTGATCTTCATTCGTTCCAGCGAACTTCGTTTCGCCCGGTGGTCGGAAATCGACTTCAA
AAGTAAGTTATGGGTGATCCCCGAACAGCGGGAAGCGATTGAAAACGTCAAGCACTCGACTCGTGGGGCT

AAAATGAAACGTCAGCACTTCGTTCCCCTTTGCAGGCAGGCTCTCAAGATACTGAAAGAGATCCGCCAGC
TTACCTATGAAGAAGGTAACGAAGCAGAATTAATTTTCACTGGCTGTTATGATTCATTCAAACCCATGAG
TGAAAACACTATTAACAAGGCGCTACGTAAGATGGGCTATGACACCACACAGGACATCTGCGGTCATGGT
TTCCGCACACTGGCGTGTAGTGCCTTAATTGAGTCTGGTCTATGGTCAGAAGATGCTGTAGAGCTTCAAA
TGAGCCATAAGGAAAGTAACAGCGTTCGCGCAGCCTATACCCATAAGGCCAAGCATCTTGACCAACGCCG
CCTGATGCTCCAGTGGTGGGCTGATTTTCTTGATGAGAATCGGTATGAGATGGTCAGGCCGTTTGAGTTT
GCTCAGAAACAATAA

>CP022273.1:c5193700-5192426 Citrobacter freundii strain 18-1
ATGTCACTGACTGATATTAAAGCAAAAAATGCAAAACCCCTTGAGAAGGAATACAAGCTTACTGATGGCT
TTGGTATGTTCCTTCGTGTTACCCCTAAAGGTTCGAAATACTGGCAAATGGCCTACCGTTTCGAAGGGAA
GCAAAAACTTTTCTCTATCGGTGTTTACCCTGCAGTTTCTCTTTCTGACGCAAGACAACGCCGTGACGAG
GCCAGAAGGCTTCTGGCTCAGGGTATTGACCCTAATGCAAAGAAACAGGCAGAGGTTAAAGAGCTTAAAG
CCAAACGTGATAATACACGCTCCTTCAGAACAGTAGCCAAAGCGTGGTTCTCTACGAAAACAAAATGGTC
TGATGATTATGGTGATGCCGTATGGAAGCGCCTTGAAACTTATGTCTTCCCGGTAATCGGTGACAAAGAT
GTTGCCGAACTGGATACGGGTGATCTGCTGGTTCCGGTGAAAAAGGTTGAGGCACTTGGTTATCTTGAAG
TTGCCATGCGCATTCAACAATACATTACGGCAATCCTGCGTCATGCCGTCCAGCAAAAGCTGATACGCCA
TAACCCAGCCTATGATATGGAAGGTGCAGTTCAGAAACCACAAACTGAACACCGCCCTGCACTTGAGCTG
GAAGAAATACCCCAGCTACTGAACAAAATTGCCGAATACAAAGGCCGCAGGTTAACCATACTGGCAATAC
AGCTCAATCTGATGATTTTCATTCGTTCCAGTGAGCTTCGTTTCGCTCGCTGGTCTGAAATTGATTTCAA
AAGTAAGTTATGGGTGATACCCGAACAGCGTGAAGCAATTGAAAACGTCAAACATTCGACTCGTGGTGCC
AAAATGAAGCGTAAGCACTTCGTTCCCCTTTGTAAGCAAGCTATGAGGATACTCAAAGAGATCCGACAAC
TGACTTATGAAGAAGGCCATGATGATGGATTAATCTTCACTGGCTGTTATGACTCGTTTAAACCCATGAG
TGAAAACACCATCAACAAAGCCCTGCGTAATATGGGATATAACACGAAGCAGGACATCTGTGGACACGGT
TTCCGCACTCTGGCCTGTAGTGCCTTGATTGAGTCCGGTTTATGGTCAGAAGACGCTGTAGAGCTTCAGA
TGAGCCATAAGGAAAGCAACAGTGTCCGGGCAGCTTATACCCACAAGGCTAAACACCTTGAACAGCGCCG
CCTGATGCTTCAGTGGTGGGCTGATTTCCTTGATGCTAACAGCAACGATATGGTCAGGCCGTTTGAGTTT
GCTTCAAATAAATAA

>CP007483.2:c1900930-1899659 Salmonella enterica subsp. enterica
serovar Anatum str. USDA-ARS-USMARC-1175
ATGTCACTGACTGATATTAAAGCAAAAAATGCCAAACCCCTTGAGAAGGAATACAAGCTTACTGATGGCT
TTGGTATGTTCCTTCGCGTTACCCCGAAGGGTTCCAAATACTGGCAAATGGCCTACCGTTTTGAAGGGAA
GCAAAAACTCTTCTCTATTGGTGTTTACCCTGCTGTTTCTCTTTCTGACGCAAGACAACGCCGTGATGAA
GCCAGAAGACTTCTTGCTCAGGGCATTGACCCTAATGCCAAGAAACAGGCAGAAGTTAAAGAGCTAAAAG
CTAAACGTGATAAAACACGCTCTTTCAGCGTAGTCGCTAAAGCTTGGTTCTCCACGAAAACAAAATGGTC
TAAAGATTATGGTGATTCCGTATGGAAGCGCCTTGAAACCTATGTCTTCCCGACAATTGGCGATAAAGAT
GTTGCCGAACTGGATACGGGTGATCTGCTGGTTCCAGTGAAAAAGGTTGAGGCACTTGGCTATCTTGAAG
TTGCCATGCGCATTCAACAATACATTACTGCGATCCTGCGTCATGCTGTTCAGCAGAAACTTATACGTCA
TAACCCGGCCTATGATATGGAAGGTGCTATTCAGAAACCGCAGACTGAACACCGCCCTGCACTGGAACTG
GAAGAAATACCCCAACTACTGAAAAAAATTGCCGAATACAAAGGCCGCAGGTTAACCATACTGGCAATAC
AGCTCAATCTGATGATTTTCATTCGTTCCAGTGAGCTGCGTTTCGCTCGCTGGTCAGAAATTGATTTCAA
AAGTAAGTTATGGGTGATACCCGAACAGCGTGAAGCGATTGAAAACGTCAAACATTCAACTCGTGGGGCT
AAAATGAAGCGTAAGCACTTCGTTCCCCTTTGTAAGCAGGCCATGAAGATACTCAAAGAGATCCGACAAC
TGACTTATGAAGAAGGCCATGATGATGGATTAATCTTTACTGGCTGTTATGACTCGTTTAAGCCCATGAG
CGAAAATACCATCAACAAAGCCCTTCGCAATATGGGCTATGACACGAAGCAGGACATCTGTGGGCACGGT
TTTCGCACGCTGGCCTGTAGTGCCTTAATTGAGTCAGGTTTGTGGTCAGAAGACGCTGTAGAGCTTCAGA
TGAGTCATAAGGAAAGCAACAGCGTCCGTGCTGCTTATACCCATAAGGCTAAACACCTTGACCAGCGCCG
ACTGATGCTCCAGTGGTGGGCTGACTTCCTTGATGCCAATCGAAACGATATGGTCAGGCCGTTTGAGTTT
GCCCAAAGGTGA

>CP023346.1:2221844-2223115 Escherichia coli strain ETEC-2265
ATGTCACTGACTGATATTAAAGCAAAAAATGCCAAACCCCTTGAGAAGGAATACAAGCTTACTGATGGCT
TTGGTATGTTCCTTCGCGTTACCCCGAAGGGTTCCAAATACTGGCAAATGGCCTACCGTTTTAAAGGGAA
GCAAAAACTCTTCTCTATTGGTGTTTACCCTGCTGTTTCTCTTTCTGACGCAAGACAACGCCGTGATGAA
GCCAGAAGGCTTCTTGCTCAGGGCATTGACCCTAATGCCAAGAAACAGGCAGAAGTTAAAGAGCTAAAAG
CTAAACGTGATAAAACACGCTCTTTCAGCGTAGTCGCTAAAGCTTGGTTCTCCACGAAAACAAAATGGTC
TAAAGATTATGGTGATTCCGTATGGAAGCGCCTTGAAACCTATGTCTTCCCGACAATTGGCGATAAAGAT

GTTGCCGAACTGGATACGGGTGATCTGCTGGTTCCAGTGAAAAAGGTTGAGGCACTTGGCTATCTTGAAG
TTGCCATGCGCATTCAACAATACATTACTGCGATCCTGCGTCATGCTGTTCAGCAGAAACTTATACGTCA
TAACCCGGCCTATGATATGGAAGATGCTATTCAGAAACCGCAGACTGAACACCGCCCTGCACTGGAACTG
GAAGAAATACCCCAACTACTGAAAAAAATTGCCGAATACAAAGGCCGCAGGTTAACCATACTGGCAATAC
AGCTCAATCTGATGATTTTCATTCGTTCCAGTGAGCTGCGTTTCGCTCGCTGGTCAGAAATTGATTTCAA
AAGTAAGTTATGGGTGATACCCGAACAGCGTGAAGCGATTGAAAACGTCAAACATTCAACTCGTGGGGCT
AAAATGAAGCGTAAGCACTTCGTTCCCCTTTGTAAGCAGGCCATGAAGATACTCAAAGAGATCCGACAAC
TGACTTATGAAGAAGGCCATGATGATGGATTAATCTTTACTGGCTGTTATGACTCGTTTAAGCCCATGAG
CGAAAATACCATCAACAAAGCCCTTCGCAATATGGGCTATGACACGAAGCAGGACATCTGTGGGCACGGT
TTTCGCACGCTGGCCTGTAGTGCCTTAATTGAGTCAGGTTTGTGGTCAGAAGACGCTGTAGAGCTTCAGA
TGAGTCATAAGGAAAGCAACAGCGTCCGTGCTGCTTATACCCATAAGGCTAAACACCTTGACCAGCGCCG
ACTGATGCTCCAGTGGTGGGCTGACTTCCTTGATGCCAATCGAAACGATATGGTCAGGCCGTTTGAGTTT
GCCCAAAGGTGA

>CP013112.1:1592022-1593296 Escherichia coli strain YD786
ATGTCACTTACTGATACTAAAGTAAAAAATGCCAGACCAGCGGAAAAAGCCGTCAAGCTCACTGATGGAT
TTGGTCTCTACCTTCTTGTGCATCCCAATGGTTCAAAATACTGGCAGTTAGGCTATCGCTTCGTTGGTAA
ACAGAAAGTGTTTTCCATTGGTGTTTACCCTGCAGTTTCTCTTGCTGATGCCAGACAACGACGTGATGAA
GCAAAAAAGCTGCTTGCTCAGGGAATCGATCCTAACGCTAAAAAACAGGCTGATGAAAAAGCTCTGCAGG
AAAAGCGGGATAAAACCCGTTCGTTCCGTGTCGTCGCCAGAAGCTGGTTTGCCACCAAAACAAAATGGTC
AGAAGATTACGCCGATACGGTATGGAAGCGCCTTGAGACCTATGTATTCCCGGACATTGGTGACAGCAAC
GTTTCAGATCTGGATACGGGTGATCTGCTTGTTCCTGTTAAAAAAGCAGAAACGCTCGGCTATCTTGAAA
TTGCCATGCGGATCAAGCAATACATCACCGCCATCCTGCGTCACGCCGTCCAGCAAAAACTTATGCGCCA
TAATCCTGCTTATGATATGGAAGGTGCAGTGCAGAAACCAGAGACTGAGCACCGTCCCGCACTGGAGCTG
GAAGAGATCCCCCTGCTACTTGAACGTATTGATGCCTACAAAGGCCGTAGACTTACCACACTGGCGATTA
AACTCAATCTGTTGATTTTCATTCGTTCCAATGAACTCCGCTATGCACGATGGTCAGAAATCGACTTCAA
CAGTAAGTTATGGGTGATACCAGAAAAGCGTGAAGCGATTGAGCGCGTCAAATATTCCACGCGTGGAGCA
AAAATGAAACGCCAGCACTTTGTTCCCCTATGCAGGCAAGCTCTTAAGATACTGAAAGAGATCCGTCAGC
TTACCTATGAAGAAGGTAACGAAGCCGGATTAATTTTAACTGGCTGTTATGACTCATTCAAACCCATGAG
TGAAAACACCATCAATAAAGCGCTGCGTAAGATGGGCTATGATACGAAGCAAGACATCTGTGGACACGGC
TTTCGCACACTGGCCTGTAGTGCCTTAATTGAGTCAGGTTTGTGGTCTGAAGATGCGGTAGAACTCCAGA
TGAGCCACAAGGAAAGCAACAGCGTTCGTGCTGCTTATACCCACAAGGCCAAGCATCTTGAGCAGCGAAG
GTTGATGCTACAATGGTGGGCAGATTTTCTTGATGCTAACCGGGATGGGATGGTCAGGCCGTTTGAGTTT
GCTCAGAAACAATAA

>CP012487.1:c1917234-1915960 Enterobacter sp. FY-07
ATGTCACTGACTGATATTAAAGCAAAAAATGCAAAACCCCTTGAGAAGGAATACAAGCTTACTGATGGCT
TTGGTATGTTCCTTCGTGTTACCCCTAAAGGTTCGAAATACTGGCAAATGGCTTACCGCTTCGAAGGGAA
ACAAAAACTCTTCTCTATTGGTGTTTACCCTGCAGTTTCTCTTTCTGACGCAAGACAACGCCGTGACGAG
GCCAGAAGGCTTCTGGCTCAGGGTATTGACCCTAATGCAAAGAAACAGGCAGAGGTTAAAGAGCTTAAAG
CCAAACGTGATAATACACGCTCCTTCAGAACAGTAGCCAAAGCGTGGTTCTCCACGAAAACAAAATGGTC
TGATGATTATGGTGATGCCGTATGGAAGCGCCTTGAAACTTATGTCTTCCCGGTAATCGGTGACAAAGAT
GTTGCCGAACTGGATACGGGTGATCTGCTGGTTCCGGTGAAAAAAGTTGAGGCTCTTGGTTATCTTGAAG
TTGCCATGCGCATTCAACAATACATTACGGCAATCCTGCGTCATGCCGTCCAGCAAAAGCTGATACGCCA
TAACCCAGCCTATGATATGGAAGATGCAGTTCAGAAACCACAAACTGAACACCGCCCTGCACTTGAGCTG
GAAGAAATACCCCAGCTACTGAACAAAATTGCCGAATACAAAGGCCGCAGGTTAACCATACTGGCAATAC
AGCTCAATCTGATGATTTTCATTCGTTCCAGTGAGCTTCGTTTCGCTCGCTGGTCTGAAATTGATTTCAA
AAGTAAGTTATGGGTGATACCCGAACAGCGTGAAGCAATTGAAAACGTCAAACATTCGACTCGTGGTGCC
AAAATGAAGCGTAAGCACTTCGTTCCCCTTTGTAAGCAAGCTATGAGGATACTAAAAGAGATCCGACAAC
TGACTTATGAAGAAGGCCATGATGATGGATTAATCTTCACTGGCTGTTATGACTCGTTTAAGCCCATGAG
TGAAAACACCATCAACAAAGCTCTTCGCAATATGGGATATAACACGAAGCAGGACATCTGTGGACACGGT
TTCCGCACTCTGGCCTGTAGTGCCTTAATTGAGTCCGGGCTATGGTCAGAAGACGCTGTAGAGCTTCAGA
TGAGCCATAAGGAAAGCAATAGTGTCCGGGCAGCTTATACCCACAAGGCTAAACACCTTGATCAACGCCG
TCTGATGCTTCAGTGGTGGGCCGATTTCCTTGATGCTAACAGCAATCATATGGTCAGGCCGTTTGAGTTT
GCTTCAAATAAATAA

>CP006918.1:c1903820-1902546 Klebsiella pneumoniae 30684/NJST258_2
ATGTCACTGACTGATATCAAAGCAAAAAATGCAAAACCCCTTGAGAAGGAATACAAGCTGACTGATGGCT

TTGGTATGTTCCTTCGCGTTACCCCGAAGGGTTCCAAATACTGGCAAATGGCTTACCGCTTCGAAGGGAA
ACAAAAACTCTTCTCTATTGGTGTTTACCCTGCAGTTTCTCTTTCTGACGCAAGACAACGCCGTGACGAG
GCCAGAAGGCTTCTGGCTCAGGGTATTGACCCTAATGCAAAGAAACAGGCAGAGGTTAAAGAGCTTAAAG
CCAAACGTGATAATACACGCTCCTTCAGAACAGTAGCCAAAGCGTGGTTCTCTACGAAAACAAAATGGTC
TGATTATTATGGTGATGCCGTATGGAAGCGCCTTGAAACTTATGTCTTCCCGGTAATCGGTGACAAAGAT
GTTGCCGAACTGGATACGGGTGATCTGCTGGTTCCGGTGAAAAAAGTTGAGGCTCTTGGTTATCTTGAAG
TTGCCATGCGCATTCAACAATACATTACGGCAATCCTGCGTCATGCCGTCCAGCAAAAGCTGATACGCCA
TAACCCAGCCTATGATATGGAAGGTGCAGTTCAGAAACCACAAACTGAACACCGCCCTGCACTTGAGCTG
GAAGAAATACCCCAGCTACTGAACAAAATTGCCGAATACAAAGGCCGCAGGTTAACCATACTGGCAATAC
AGCTCAATCTGATGATTTTCATTCGTTCCAGTGAGCTTCGTTTCGCTCGCTGGTCTGAAATTGATTTCAA
AAGTAAGTTATGGGTGATACCCGAACAGCGTGAAGCGATTGAAAACGTCAAACATTCAACTCGTGGGGCT
AAAATGAAGCGTAAGCACTTCGTTCCCCTTTGTAAGCAGGCCATGAAGATACTCAAAGAGATCCGACAAC
TGACTTATGAAGAAGGCCATGATGATGGATTAATCTTTACTGGCTGTTATGACTCGTTTAAGCCCATGAG
CGAAAATACCATCAACAAAGCCCTTCGCAATATGGGCTATGACACGAAGCAGGACATCTGCGGGCACGGT
TTTCGCACGCTGGCCTGTAGTGCCTTAATTGAGTCAGGTTTGTGGTCAGAAGACGCTGTAGAGCTTCAGA
TGAGTCATAAGGAAAGCAACAGCGTCCGTGCTGCTTATACCCATAAGGCTAAACACCTTGACCAGCGCCG
ACTGATGCTCCAGTGGTGGGCTGATTTCCTTGATGCTAACAGCAACGGTATGGTCAGGCCGTTTGAGTTT
GCTTCCAATAAATAA

>CP015750.1:59853-61130 Pectobacterium wasabiae CFBP 3304
ATGTCACTTACTGATACTAAAGTAAAAAATGCCAAGCCGTCAGAAAAGGCGGTGAAGCTCACTGACGGGT
TCGGCCTCTACTTGCTGGTTCATCCCAATGGTTCCAAATACTGGCAGTTAGGCTATCGCTTCGAAGGAAA
ACAGAAGGTGTTTTCCATCGGCGTCTACCCTGCTGTTTCTCTGGCTGATGCAAGACAACGCCGGGATGAA
GCAAAAAAACTGCTAGCTTCTGGAATTGACCCTAGCGCTAAAAAGCAGGCTGACAACAAAACCATTCAAG
AGAAGCGTAACAATACCCGCGCTTTCAAGACCGTTGCCAAAAGCTGGTTTGCCACCAAAACCACATGGTC
GGAAGATTATCAGCGTTCTGTCTGGACACGACTGGAAACCTACCTATTTCCTGACATAGGTAACAAAGAC
ATTGCTGAGCTGGATACAGGCGATCTGCTGATTCCCATCAAAAAGATAGAGAAGCTGGGTTATCTGGAAA
TCGCTATGCGGGTGAAACAGTACGCGACCGCCATCATGCGTTATGCCGTCCAGCAAAAGATGATCCGTTT
CAATCCTGCTTATGACTTGGAAGGTGCGGTTCAGAAGCCACAGACGGAACACCGCCCTGCTATTGAACTG
GAAGAGATACCTACCCTACTGGAACGTATTGAAGCCTATAAAGGCCGTAGCAGGCTGACCCAATTGGCGA
TAAAACTCAATCTACTGATTTTTGTGCGTTCTAGTGAACTTCGCTTTGCCCGGTGGTCAGAGATCGATTT
CAAAAGCGCTTTGTGGGTTATCCCTGAACAGCGTGAAGCCATTGAAGGGATCAAACATTCAGGCCGTGGT
GCCAAAATGCGTAGGAAGCATTATGTTCCACTGTGTCGTCAGGCGCTGGCAATTTTGGAAGAGCTGAAAG
ACCTCACCTATGACGTTAACGGTGATGACGGCTTTATCCTGACTGGCTGTTATGATGCGATGAAGCCGAT
GAGTGAAAACACCATCAACAAGGCACTGCGCAAAATGGGCTATGACACCAAAACTGACCTGTGTGGTCAT
GGTTTCCGAACGCTGGCGTGTAGTGCCTTAATTGAATCGGGTATTTGGCTTGAAGATGTGGTTGAACTTC
AGATGAGCCACATGGAAAAGAACAACGTTCGCGCTGCCTACACTCACAAGGCCAAACACCTTGAGCAACG
CCGCCTCATGTTGCAATGGTGGGCTGATTTTCTGGATGCTAACAGCAACGCGATGGTCAGGCCGTTTGAG
TTTGCGCAGAAGGGATAA

>BX950851.1:3236381-3237667 Pectobacterium atrosepticum SCRI1043
ATGTCACTGACTGATACTAAAGTAAAAAATGCCAAGCCATCAGAAAAGGTGGTTAAGCTCACTGACGGGT
TCGGCCTCTATCTGCTGGTGCATACTAACGGTTCAAAATACTGGCAGTTAGGCTATCGCTTCGAAGGGAA
ACAGAAGGTGTTCTCCATCGGCGTCTATCCTGCTGTTTCTCTGGCTAATGCAAGACAGCGCCGAGATGAA
GCAAAAAAGCTGTTAGCCGCAGGAGTAGATCCCAGCGCCAAAAAACGGGCTGACAACAAGTCCGTTCAAG
AAAAGCGGAACAATACCCGCGCTTTCAAAACGGTTGCCAAAAGCTGGTTTGCCACCAAAACCACATGGTC
GGAAGATTATCAGCGTTCTGTATGGACACGACTGGAAACCTACCTGTTCCCTGATATTGGCAACAAAGAT
ATTGCTGAACTGGATACAGGCGATCTGCTGGTTCCCATCAAAAAGATAGAGAAGCTGGGTTATCTGGAAA
TCGCCATGAGGGTAAAACAGTACGCCACAGCCATCATGCGTTATGCCGTCCAGCAAAAGATGATCCGTTT
CAATCCGGCCTATGATTTGGAAGGTGCGGTTCAGAAGCCACAGACGGAACACCGTCCCGCTATCGAACTG
GAAGAGATACCTACCCTGTTGGAACGCATTGAAGGCTATAAAGGCCGTAGCAGGCTGACCCAATTAGCGA
TAAAACTCAATCTGCTGATTTTTATCCGTTCCAGTGAACTCCGCTTTGCCCGATGGTCAGAGATCGATTT
CAAAAGCGGTTTGTGGGTTATCCCTGAACAGCGAGAAGCTATTAAAGGGATCAAGCATTCAGACCGTGGT
GCCAAAATGCGCAGAAAGCATTATGTTCCTCTATGCGAACAAGCACTGGTAATTTTGGGAGAGCTTAAAG
ACCTCACCTATGACGTTAACGGTGATGACGGCTTTATCCTGACAGGCTGTTATGATGCGATGAAACCGAT
GAGTGAAAACACCATCAACAAAGCACTGCGCAAAATGGGTTATGACACCAAAACTGATCTGTGCGGTCAT
GGTTTCCGAACGCTGGCGTGTAGTGCCTTGATTGAATCGGGTATCTGGCCTGAAGATGTGGTTGAACTTC
AGATGAGCCACATGGAAAAGAACAACGTTCGTGCTGCCTATACCCATAAGGCTAAACACCTTGAGCAACG

CCGCCTGATGTTGCAATGGTGGGCTGATTTTCTGGATGCTAACGGCAGCGGGATGGTTAGGCCGTTTGAG
TTTGCTCAAGGAGCGACTCAATATTAG

>CP012635.1:c1941415-1940144 Escherichia coli strain SF-088
ATGTCACTGACTGATATTAAAGCAAAAAATGCCAAACCCCTTGAGAAGGAATACAAGCTTACTGATGGCT
TTGGTATGTTCCTTCGCGTTACCCCGAAGGGTTCCAAATACTGGCAAATGGCCTACCGTTTTGAAGGGAA
GCAAAAACTCTTCTCTATTGGTGTTTACCCTGCTGTTTCTCTTTCTGACGCAAGACAACGCCGTGATGAA
GCCAGAAGGCTTCTTGCTCAGGGCATTGACCCTAATGCCAAGAAACAGGCAGAAGTTAAAGAGCTAAAAG
CTAAACGTGATAAAACACGCTCTTTCAGCGTAGTCGCTAAAGCTTGGTTCTCCACGAAAACAAAATGGTC
TAAAGATTATGGTGATTCCGTATGGAAGCGCCTTGAAACCTATGTCTTCCCGACAATTGGCGATAAAGAT
GTTGCCGAACTGGATACGGGTGATCTGCTGGTTCCAGTGAAAAAGGTTGAGGCACTTGGCTATCTTGAAG
TTGCCATGCGCATTCAACAATACATTACTGCGATCCTGCGTCATGCTGTTCAGCAGAAACTTATACGTCA
TAACCCGGCCTATGATATGGAAGGTGCTATTCAGAAACCGCAGACTGAACACCGCCCTGCACTGGAACTG
GAAGAAATACCCCAACTACTGAAAAAAATTGCCGAATACAAAGGCCGCAGGTTAACCATACTGGCAATAC
AGCTCAATCTGATGATTTTCATTCGTTCCAGTGAGCTGCGTTTCGCTCGCTGGTCAGAAATTGATTTCAA
AAGTAAGTTATGGGTGATACCCGAACAGCGTGAAGCGATTGAAAACGTCAAACATTCAACTCGTGGGGCT
AAAATGAAGCGTAAGCACTTCGTTCCCCTTTGTAAGCAGGCCATGAAGATACTCAAAGAGATCCGACAAC
TGACTTATGAAGAAGGCCATGATGATGGATTAATCTTTACTGGCTGTTATGACTCGTTTAAGCCCATGAG
CGAAAATACCATCAACAAAGCCCTTCGCAATATGGGCTATGACACGAAGCAGGACATCTGTGGGCACGGT
TTTCGCACGCTGGCCTGTAGTGCCTTAATTGAGTCAGGTTTGTGGTCAGAAGACGCTGTAGAGCTTCAGA
TGAGTCATAAGGAAAGCAACAGCGTCCGTGCTGCTTATACCCATAAGGCTAAACACCTTGACCAGCGCCG
ACTGATGCTCCAGTGGTGGGCTGACTTCCTTGATGCCAATCGAAACGATATGGTCAGGCCGTTTGAGTTT
GCCCAAAGGTGA

>CP021950.1:c5170217-5168943 Klebsiella pneumoniae strain AR_0148
ATGTCACTGACTGATATCAAAGCAAAAAATGCAAAACCCCTTGAGAAGGAATACAAGCTGACTGATGGCT
TTGGTATGTTCCTTCGCGTTACCCCGAAGGGTTCCAAATACTGGCAAATGGCTTACCGCTTCGAAGGGAA
ACAAAAACTCTTCTCTATTGGTGTTTACCCTGCAGTTTCTCTTTCTGACGCAAGACAACGCCGTGACGAG
GCCAGAAGGCTTCTGGCTCAGGGTATTGACCCTAATGCAAAGAAACAGGCAGAGGTTAAAGAGCTTAAAG
CCAAACGTGATAATACACGCTCCTTCAGAACAGTAGCCAAAGCGTGGTTCTCTACGAAAACAAAATGGTC
TGATTATTATGGTGATGCCGTATGGAAGCGCCTTGAAACTTATGTCTTCCCGGTAATCGGTGACAAAGAT
GTTGCCGAACTGGATACGGGTGATCTGCTGGTTCCGGTGAAAAAAGTTGAGGCTCTTGGTTATCTTGAAG
TTGCCATGCGCATTCAACAATACATTACGGCAATCCTGCGTCATGCCGTCCAGCAAAAGCTGATACGCCA
TAACCCAGCCTATGATATGGAAGGTGCAGTTCAGAAACCACAAACTGAACACCGCCCTGCACTTGAGCTG
GAAGAAATACCCCAGCTACTGAACAAAATTGCCGAATACAAAGGCCGCAGGTTAACCATACTGGCAATAC
AGCTCAATCTGATGATTTTCATTCGTTCCAGTGAGCTTCGTTTCGCTCGCTGGTCTGAAATTGATTTCAA
AAGTAAGTTATGGGTGATACCCGAACAGCGTGAAGCGATTGAAAACGTCAAACATTCAACTCGTGGGGCT
AAAATGAAGCGTAAGCACTTCGTTCCCCTTTGTAAGCAGGCCATGAAGATACTCAAAGAGATCCGACAAC
TGACTTATGAAGAAGGCCATGATGATGGATTAATCTTTACTGGCTGTTATGACTCGTTTAAGCCCATGAG
CGAAAATACCATCAACAAAGCCCTTCGCAATATGGGCTATGACACGAAGCAGGACATCTGCGGGCACGGT
TTTCGCACGCTGGCCTGTAGTGCCTTAATTGAGTCAGGTTTGTGGTCAGAAGACGCTGTAGAGCTTCAGA
TGAGTCATAAGGAAAGCAACAGCGTCCGTGCTGCTTATACCCATAAGGCTAAACACCTTGACCAGCGCCG
ACTGATGCTCCAGTGGTGGGCTGATTTCCTTGATGCTAACAGCAACGGTATGGTCAGGCCGTTTGAGTTT
GCTTCCAATAAATAA

>AP013063.1:2720507-2721781 Serratia marcescens SM39
ATGTCACTGACTGATATTAAAGTAAAAAATGCAAAACCCCTTGAGAAGGAATACAAGCTTACTGATGGCT
TTGGTATGTTCCTTCGTGTTACCCCTAAAGGTTCGAAATACTGGCAAATGGCTTACCGCTTCGAAGGGAA
ACAAAAACTCTTCTCTATTGGTGTTTACCCTGCTGTTTCTCTTTCTGACGCAAGGCAACGCCGTGACGAG
GCCAGAAGGCTTCTGGCTCAGGGTATTGACCCTAATGCAAAGAAACAGGCAGAGGTTAAAGAGCTTAAAG
CCAAACGTGATAATACACGCTCCTTCAGAACAGTAGCCAAAGCGTGGTTCTCTACGAAAACAAAATGGTC
TGATGATTATGGTGATGCCGTATGGAAGCGCCTTGAAACTTATGTCTTCCCGGTAATCGGTGACAAAGAT
GTTGCCGAGCTTGATACGGGTGATCTGCTGGTTCCGGTGAAAAAAGTGGAGGCACTTGGTTATCTTGAAG
TTGCCATGCGCATTCAACAATACATTACGGCAATCCTGCGTCATGCCGTCCAGCAAAAGCTGATACGCCA
TAACCCAGCCTATGATATGGAAGGTGCAGTTCAGAAACCACAAACTGAACACCGCCCTGCACTTGAGCTG
GAAGAAATACCCCAGCTACTGAACAAAATTGCCGAATACAAAGGCCGCAGGTTAACCATACTGGCAATAC
AGCTCAATCTGATGATTTTCATTCGTTCCAGTGAGCTTCGTTTCGCTCGCTGGTCTGAAATTGATTTCAA
AAGTAAGTTATGGGTGATACCCGAACAGCGTGAAGCAATTGAAAACGTCAAACATTCGACTCGTGGTGCC

AAAATGAAGCGTAAGCACTTCGTTCCCCTTTGTAAGCAAGCTATGAGGATACTAAAAGAGATCCGACAAC
TGACTTATGAAGAAGGTCAAGATGATGGGTTAATCTTTACTGGCTGTTATGACTCGTTTAAGCCCATGAG
TGAAAACACCATCAACAAAGCCCTCCGCAATATGGGCTATGACACGAAGCAGGACATCTGTGGACACGGT
TTCCGCACTCTGGCCTGTAGTGCCTTAATTGAGTCCGGGCTATGGTCAGAAGACGCTGTAGAGCTTCAAA
TGAGCCATAAGGAAAGCAACAGCGTCCGTGCTGCTTATACCCACAAGGCAAAACATCTTGAGCAGCGTCG
CCTGATGCTTCAGTGGTGGGCTGATTTCCTTGATGCTAACAGCAACGATATGGTCAGGCCGTTTGAGTTT
GCTTCAAATAAATAA

>CP011642.1:c4518665-4517391 Serratia marcescens strain CAV1492
ATGTCACTGACTGATATTAAAGCAAAAAATGCAAAACCCCTTGAGAAGGAATATAAGCTGACTGATGGCT
TTGGTATGTTCCTTCGTGTTACCCCTAAAGGTTCGAAATACTGGCAAATGGCCTACCGTTTCGAAGGAAA
GCAAAAACTTTTCTCTATCGGTGTTTACCCTGCTGTTTCTCTTTCTGACGCAAGACAACGCCGTGACGAG
GCCAGAAGGCTTCTGGCTCAGGGTATTGACCCTAATGCAAAGAAACAGGCAGAGGTTAAAGAGCTTAAAG
CCAAACGTGATAATACACGCTCCTTCAGAACAGTAGCCAAAGCGTGGTTCTCCACGAAAACAAAATGGTC
TGATGATTATGGTGATGCCGTATGGAAGCGCCTTGAAACTTATGTCTTCCCGGTAATCGGTGACAAAGAT
GTTGCCGAACTGGATACGGGTGATCTGCTGGTTCCGGTGAAAAAAGTTGAGGCTCTTGGTTATCTTGAAG
TTGCCATGCGCATTCAACAATACATTACGGCAATCCTGCGTCATGCCGTCCAGCAAAAGCTGATACGCCA
TAACCCAGCCTATGATATGGAAGGTGCAGTTCAGAAACCACAAACTGAACACCGCCCTGCACTTGAGCTG
GAAGAAATACCCCAGCTACTGAACAAAATTGCCGAATACAAAGGCCGCAGGTTAACCATACTGGCAATAC
AGCTCAATCTGATGATTTTCATTCGTTCCAGTGAGCTTCGTTTCGCTCGCTGGTCAGAAATTGATTTCAA
AAGTAAGTTATGGGTGATACCCGAACAGCGTGAAGCAATTGAAAACGTCAAACATTCGACTCGTGGGGCT
AAAATGAAGCGTAAGCACTTCGTTCCCCTCTGTAAGCAAGCTATGAGGATACTAAAAGAGATCCGACAAC
TGACTTATGAAGAAGGCCATGATGATGGATTAATCTTCACTGGCTGTTATGACTCGTTTAAACCCATGAG
TGAAAACACCATCAACAAAGCCCTGCGTAATATGGGATATAACACGAAGCAGGACATCTGTGGACACGGT
TTCCGCACTCTGGCCTGTAGTGCCTTAATTGAGTCCGGGCTATGGTCAGAAGACGCTGTAGAGCTTCAAA
TGAGCCATAAGGAAAGCAACAGCGTCCGTGCTGCTTATACCCACAAGGCTAAACACCTTGATCAACGCCG
TCTGATGCTTCAGTGGTGGGCCGATTTCCTTGATGCTAACAGCAATCATATGGTCAGGCCGTTTGAGTTT
GCTTCAAATAAATAA

>CP011602.1:c1111277-1110003 Kluyvera intermedia strain CAV1151
ATGTCACTGACTGATATTAAAGCAAAAAATGCAAAACCCCTTGAGAAGGAATACAAGCTTACTGATGGCT
TTGGTATGTTCCTTCGTGTTACCCCTAAAGGTTCGAAATACTGGCAAATGGCTTACCGCTTCGAAGGGAA
ACAAAAACTCTTCTCTATTGGTGTTTACCCTGCAGTTTCTCTTTCTGACGCAAGACAACGCCGTGACGAG
GCCAGAAGGCTTCTGGCTCAGGGTATTGACCCTAATGCAAAGAAACAGGCAGAGGTTAAAGAGCTTAAAG
CCAAACGTGATAATACACGCTCCTTCAGAACAGTAGCCAAAGCGTGGTTCTCCACGAAAACAAAATGGTC
TGATGATTATGGTGATGCCGTATGGAAGCGCCTTGAAACTTATGTCTTCCCGGTAATCGGTGACAAAGAT
GTTGCCGAACTGGATACGGGTGATCTGCTGGTTCCGGTGAAAAAAGTTGAGGCTCTTGGTTATCTTGAAG
TTGCCATGCGCATTCAACAATACATTACGGCAATCCTGCGTCATGCCGTCCAGCAAAAGCTGATACGCCA
TAACCCAGCCTATGATATGGAAGGTGCAGTTCAGAAACCACAAACTGAACACCGCCCTGCACTTGAGCTG
GAAGAAATACCCCAGCTACTGAACAAAATTGCCGAATACAAAGGCCGCAGGTTAACCATACTGGCAATAC
AGCTCAATCTGATGATTTTCATTCGTTCCAGTGAGCTTCGTTTCGCTCGCTGGTCTGAAATTGATTTCAA
AAGTAAGTTATGGGTGATACCCGAACAGCGTGAAGCAATTGAAAACGTCAAACATTCGACTCGTGGTGCC
AAAATGAAGCGTAAGCACTTCGTTCCCCTCTGTAAGCAAGCTATGAGGATACTAAAAGAGATCCGACAAC
TGACTTATGAAGAAGGCCATGATGATGGATTAATCTTCACTGGCTGTTATGACTCGTTTAAACCCATGAG
TGAAAACACCATCAACAAAGCCCTGCGTAATATGGGATATAACACGAAGCAGGACATCTGTGGACACGGT
TTCCGAACACTGGCCTGTAGTGCCTTAATTGAGTCCGGGCTATGGTCAGAAGACGCTGTAGAGCTTCAGA
TGAGCCATAAGGAAAGCAACAGTGTCCGGGCAGCTTATACCCACAAGGCTAAACACCTTGATCAACGCCG
TCTGATGCTTCAGTGGTGGGCTGATTTCCTTGATGCTAACAGTAACGATATGGTCAGACCGTTTGAGTTT
GCTTCAAATAAATAA

>CU928162.2:2237821-2239095 Escherichia coli ED1a chromosome
ATGTCACTGACTGATATTAAAGCAAAAAATGCAAAACCCCTTGAGAAGGAATACAAGCTGACTGATGGCT
TTGGTATGTTCCTTCGTGTTACCCCTAAAGGTTCGAAATACTGGCAAATGGCTTACCGCTTCGAAGGGAA
ACAAAAACTCTTCTCTATTGGTGTTTACCCTGCAGTTTCTCTTTCTGACGCAAGACAACGCCGTGACGAG
GCCAGAAGGCTTCTGGCTCAGGGTATTGACCCTAATGCAAAGAAACAGGCAGAGGTTAAAGAGCTTAAAG
CCAAACGTGATAATACACGCTCCTTCAGAACAGTAGCCAAAGCGTGGTTCTCTACGAAAACAAAATGGTC
TGATGATTATGGTGATGCCGTATGGAAGCGCCTTGAAACTTATGTCTTCCCGGTAATCGGTGACAAAGAT
GTTGCCGAACTGGATACGGGTGATCTGCTGGTTCCGGTGAAAAAAGTGGAGGCACTTGGTTATCTTGAAG

TTGCCATGCGCATTCAACAATACATTACGGCAATCCTGCGTCATGCCGTCCAGCAAAAGCTGATACGCCA
TAACCCAGCCTATGATATGGAAGGTGCAGTTCAGAAACCACAAACTGAACACCGCCCTGCACTTGAGCTG
GAAGAAATACCCCAGCTACTTAACAAAATTGCCGAATACAAAGGCCGCAGGTTAACCATACTGGCAATAC
AGCTCAATCTGATGATTTTCATTCGTTCCAGTGAGTTGCGTTTCGCTCGCTGGTCTGAAATTGATTTCAA
AAGTAAGTTATGGGTGATACCCGAACAGCGTGAAGCAATTGAAAACGTCAAACATTCGACTCGTGGGGCT
AAAATGAAGCGTAAGCACTTCGTTCCCCTTTGTAAGCAGGCCATGAAGATACTCAAAGAGATCCGACAAC
TGACTTATGAAGAAGGCCATGATGATGGATTAATCTTCACTGGCTGTTATGACTCGTTTAAACCCATGAG
TGAAAACACCATCAACAAAGCCCTGCGTAATATGGGATATAACACGAAGCAGGACATCTGTGGACACGGT
TTCCGCACTCTGGCCTGTAGTGCCTTAATTGAGTCCGGGCTATGGTCAGAAGACGCTGTGGAGCTTCAAA
TGAGCCATAAGGAAAGCAACAGCGTCCGTGCTGCTTATACCCACAAGGCAAAACATCTTGAGCAACGTCG
CCTGATGCTCCAATGGTGGGCTGACTACCTTGATGCAAGCAGAAACGGTATGGTAAGGCCGTTTGAGTTT
GCTACAAATAAATAA

>CP003785.1:c1952189-1950915 Klebsiella pneumoniae subsp. pneumoniae
1084
ATGTCACTGACTGATATTAAAGCAAAAAATGCAAAACCCCTTGAGAAGGAATACAAGCTTACTGATGGCT
TTGGTATGTTCCTTCGTGTTACCCCTAAAGGTTCGAAATACTGGCAAATGGCTTACCGCTTCGAAGGGAA
ACAAAAACTCTTCTCTATTGGTGTTTACCCTGCAGTTTCTCTTTCTGACGCAAGACAACGCCGTGACGAG
GCCAGAAGGCTTCTGGCTCAGGGTATTGACCCTAATGCAAAGAAACAGGCAGAGGTTAAAGAGCTTAAAG
CCAAACGTGATAATACACGCTCCTTCAGAACAGTAGCCAAAGCGTGGTTCTCCACGAAAACAAAATGGTC
TGATGATTATGGTGATGCCGTATGGAAGCGCCTTGAAACTTATGTATTCCCGGCGATTGGAGATAAAGAT
GTTGCCGAACTGGATACGGGTGACCTGCTGGTTCCGGTGAAAAAGGTTGAGGCTCTTGGTTATCTTGAAG
TTGCCATGCGCACTCAACAATACATTACGGCAATCCTGCGTCATGCCGTCCAGCAAAAGCTGATACGCCA
TAACCCAGCCTATGATATGGAAGGTGCAGTTCAGAAACCACAAACTGAACACCGCCCTGCACTTGAGCTG
GAAGAAATACCCCAGCTACTTAACAAAATTGCCGAATACAAAGGCCGCAGGTTAACCATACTGGCAATAC
AGCTCAATCTGATGATTTTCATTCGTTCCAGTGAGTTGCGTTTCGCTCGCTGGTCAGAAATTGATTTCAA
AAGTAAGTTATGGGTGATACCCGAACAGCGTGAAGCAATTGAAAACGTCAAACATTCGACTCGTGGTGCC
AAAATGAAGCGTAAGCACTTCGTTCCCCTCTGTAAGCAAGCTATAAGGATACTAAAAGAGATCCGACAAC
TGACTTATGAAGAAGGCCATGATGATGGATTAATCTTCACTGGCTGTTATGACTCGTTTAAACCCATGAG
TGAAAACACCATCAACAAAGCCCTGCGTAATATGGGATATAACACGAAGCAGGACATCTGTGGACACGGT
TTCCGCACTCTGGCCTGTAGTGCCTTAATTGAGTCCGGGCTATGGTCAGAAGACGCTGTGGAGCTTCAAA
TGAGCCATAAGGAAAGTAACAGCGTCCGTGCTGCTTATACCCACAAGGCAAAACATCTTGAGCAACGTCG
CCTGATGCTCCAATGGTGGGCTGACTACCTTGATGCAAGCAGAAACGGTATGGTAAGGCCGTTTGAGTTT
GCTACAAATAAATAA

>CP009801.1:c3911165-3909894 Yersinia intermedia strain Y228
ATGCCACTTACTGATACTAAAGTAAAAAACGCCAAGCCCCTCGATAAGGAATACAAGCTGACTGATGGCT
TTGGTATGTTCCTTCGCGTTACCCCTAAGGGTTCCAGATACTGGCAAATGGCTTACCGCTTCGAAGGGAA
GCAAAAAATCTTCTCCATAGGTGTCTACCCTGCTGTTTCACTTGCTGATGCAAGACAGCGCCGTGATGAA
GCTAAAAGACTTCTTGCTCAGGGCATTGATCCTAACGTCAAGAAACAGGCCGAAGTTAAAGAGTTGAAAG
CAAAACGCGACAACACACGATCTTTCAGGTTTGTCGCCAAGGCGTGGTTTTCCACCAAAAAGAAATGGTC
TGAAGATTATCGCAATACAGTTTTGACCCGACTTGAAACCTACATATTCCCAGATATCGGTAACAAAGAC
GTCACGGAGCTTGATACAGGTGATTTGTTAGCGCCGATCAAAAAAGTCGAAGCCCTTGGTTATCTGGAAG
TAGCCACAAGGGTTAAACAGTCCGTCACCTCTATCCTGCGTTATGCCGTCCAACAGAAGCTAATCCGCTA
CAATCCGGCCTATGATTTGGAAGGTTCAGTTCAGAAAGCTGAGACAGAACACCGCCCTGCACTGGAGCTT
GAAGAGATCCCATTGCTACTTGAGCGTGTTGATGCTTACAAAGGGCGTAGCCTTACTACACTAGCAATCA
AACTCAATCTGCTGGTTTTTGTTCGTTCCAGCGAACTCCGTTTTGCCCGATGGTCAGAAATCGACTTCAC
AAGTAAGCTGTGGGTTATCCCCGAACAACGTGAACCCATTGCAGGAGTGAAGTATTCAGGTCGTGGAGCA
AAAATGAGACGGAAACATTTCATCCCCCTGTGCCGTCAGGCAATGGAACTACTGAACGAAATTAAGGTGC
TGACCTATGAAAATGGCAACGATGATGGTTTCATCTTTACGGGTAGTTATGACAGTTTCAAACCGATGAG
CGAAAACACCATTAACAAAGCGCTGCGCAATATGGGCTATGACACGAAGCAGGACATCTGTGGACATGGT
TTTCGCACACTGGCCTGTAGTGCCTTAATTGAATCAGGTTTGTGGTCAGAAGACGCTGTAGAGCTTCAAA
TGAGCCACAAGGAAAGCAACAGCGTCCGTGCAGCTTATACTCATAAAGCTAAACACCTTGACCAACGACG
CCTAATGCTCCAGTGGTGGGCTGACTACCTTGATGCGAACAGTAGCTGTATGGTCAGGCCGTTTGAGTTT
GCAAATAAGTGA

>CP001790.1:c1876258-1874981 Pectobacterium parmentieri WPP163
ATGTCACTTACTGACACCAAAGTAAAAAAAGCTAAACCTCTTGAGAAAGAATACAAGCTTACTGACGGTT

TTGGTATGCACCTGCTTGTCCACCCGAACGGCTCTAAATACTGGCGACTTTCCTACCGCTTTGCGCAGAA
GCAAAAACTGTTAGCGTTGGGCGTTTACCCTGCCATTTCTTTGACTGACGCAAGGGAGCGTCGTGATGAA
GCGCGTAAGCTGATTGCTAATGGGATTGATCCCGGCGCTAAGAAAAAGGCAAGTATCAGGGAACAACCAG
GAGCACAGGATGAATCCCGTTCTTTTGCTGTGATGGCTCGTGCATGGGCAGAGACCAAAACCAAGTGCTC
AGAAGACTACAAGGTTAAGGTCTGGAGACGCATTGAAAACTATCTTCTGCCTGATCTGGGAAAACGTGAT
GTATCAGAGCTTGATACCAGTGATCTGCTAACCCCACTCAGAAAAGTTGAAAAATTAGGCTATCTCGACA
TCGCCATGCGTCTTAAGCAATATACAACATCGATCATGCGATACGCCGTCCAGCAGAAGATCATCAGTTA
CAACCCCGCTTATGATTTGGCAGGCACTGTCGAAAAAGGTGAGACAGCACACCGCCCTTCTATCGAGATT
TATGAAATCCCCGATCTCTTACAAAAGATCGACGACTACCATGGTCGGGGTCTTTTAACAGAATTAGCGA
TCAGGCTCACATTATTGGTTTTTGTCAGGTCAAGTGAGTTACGTTTCGCCAGATGGAACGAAATCGACTT
CAAAAAATCTCTGTGGGTTATCCCTGAACAACGAAAGGAAGTTAAGGGGGTAAAATACTCTGGCCGTGGT
GCAAAAATGAAGAGAAAGCATTTTGTTCCACTCTCCAGGCAAGCTGTGGAGATCTTAAAAGAGGTTAAGC
AGATCACTTATGGTGAAAAAGCGGGTGACGGGTTTATTTCACCGGGTTTTACGACAGTGATTCTGCGAT
GAGTTCAGGCACTATCAATAAAGCCCTTCAGCGCATGGGATATGACACCAAAACCGATCTGTGTGGGCAT
GGCTTTCGCACGTTAGCCTGTAGTGCATTAACAGAATCTGGATTATGGTCGGAAGACACGGTCGAGCTTC
AAATGAGCCATAAAGAGAAAAACACGGTTAGATCTGCTTATACCCATAAAGTCAGCCATCTTGACCAGCG
CAAACTGATGCTGCAATGGTGGGCGGATTTTCTGGATGCGAACCGCAATGGAGTAGTTAGTCCGTTTGAG
TTTGCACAGAAAGGATAG

>CP013338.1:c1916947-1915673 Raoultella ornithinolytica strain Yangling I2
ATGTCACTGACTGATATTAAAGCAAAAAATGCAAAACCCCTTGAGAAGGAATACAAGCTTACTGATGGCT
TTGGTATGTTCCTTCGTGTTACCCCTAAAGGTTCGAAATACTGGCAAATGGCTTACCGCTTCGAAGGGAA
ACAAAAACTCTTCTCTATTGGTGTTTACCCTGCAGTTTCTCTTTCTGACGCAAGACAACGCCGTGACGAG
GCCAGAAGGCTTCTGGCTCAGGGTATTGACCCTAATGCAAAGAAACAGGCAGAGGTTAAAGAGCTTAAAG
CCAAACGTGATAATACACGCACCTTCAGAACAGTAGCCAAAGCGTGGTTCTCCACGAAAACAAAATGGTC
TGATGATTATGGTGATGCCGTATGGAAGCGCCTTGAAACTTATGCCTTCCCGGTAATCGGTGACAAAGAT
GTTGCCGAACTGGATACGGGTGATCTGCTGGTTCCGGTGAAAAAAGTTGAGGCTCTTGGTTATCTTGAAG
TTGCCATGCGCATTCAACAATACATTACGGCAATCCTGCGTCATGCCGTCCAGCAAAAGCTGATACGCCA
TAACCCAGCCTATGATATGGAAGGTGCAGTTCAGAAACCACAAACTGAACACCGCCCTGCACTTGAGCTG
GAAGAAATACCCCAGCTACTGAACAAAATTGCCGAATACAAAGGCCGCAGGTTAACCATACTGGCAATAC
AGCTCAATCTGATGATTTTCATTCGTTCCAGTGAGCTTCGTTTCGCTCGCTGGTCTGAAATTGATTTCAA
AAGTAAGTTATGGGTGATACCCGAACAGCGTGAAGCAATTGAAAACGTCATACATTCGACTCGTGGTGCC
AAAATGAAGCGTAAGCACTTCGTTCCCCTCTGTAAGCAAGCTATAAGGATACTAAAAGAGATCCGACAAC
TGACTTATGAAGAAGGCCATGATGATGGATTAATCTTCACTGGCTGTTATGACTCGTTTAAACCCATGAG
TGAAAACACCATCAACAAAGCCCTGCGTAATATGGGATATAACACGAAGCAGGACATCTGTGGACACGGT
TTCCGCACTCTGGCCTGTAGTGCCTTAATTGAGTCCGGGCTATGGTCAGAAGACGCTGTGGAGCTTCAAA
TGAGCCATAAGGAAAGTAACAGCGTCCGTGCTGCTTATACCCACAAGGCAAAACATCTTGAGCAACGTCG
CCTGATGCTCCAATGGTGGGCTGACTACCTTGATGCAAGCAGAAACGGTATGGTAAGGCCGTTTGAGTTT
GCTACAAATAAATAA

>CP022154.1:3112494-3113768 Escherichia coli strain ABWA45
ATGTCACTGACTGATATTAAAGCAAAAAATGCAAAACCCCTTGAGAAGGAATACAAGCTTACTGATGGCT
TTGGTATGTTCCTTCGTGTTACCCCTAAAGGTTCGAAATACTGGCAAATGGCTTACCGCTTCGAAGGGAA
ACAAAAACTCTTCTCTATTGGTGTTTACCCTGCAGTTTCTCTTTCTGACGCAAGACAACGCCGTGACGAG
GCCAGAAGGCTTCTGGCTCAGGGTATTGACCCTAATGCAAAGAAACAGGCAGAGGTTAAAGAGCTTAAAG
CCAAACGTGATAATACACGCTCCTTCAGAACAGTAGCCAAAGCGTGGTTCTCCACGAAAACAAAATGGTC
TGATGATTATGGTGATGCCGTATGGAAGCGCCTTGAAACTTATGCCTTCCCGGTAATCGGTGACAAAGAT
GTTGCCGAACTCGATACGGGTGATCTGCTGGTTCCGGTGAAAAAAGTTGAGGCTCTTGGTTATCTTGAAG
TTGCCATGCGCATTCAACAATACATTACGGCAATCCTGCGTCATGCCGTCCAGCAAAAGCTGATACGCCA
TAACCCAGCCTATGATATGGAAGGTGCAGTTCAGAAACCACAAACTGAACACCGCCCTGCACTTGAGCTG
GAAGAAATACCCCAGCTACTGAACAAAATTGCCGAATACAAAGGCCGCAGGTTAACCATACTGGCAATAC
AGCTCAATCTGATGATTTTCATTCGTTCCAGTGAGCTTCGTTTCGCTCGCTGGTCTGAAATTGATTTCAA
AAGTAAGTTATGGGTGATACCCGAACAGCGTGAAGCAATTGAAAACGTCAAACATTCGACTCGTGGTGCC
AAAATGAAGGGTAAGCACTTCGTTCCCCTCTGTAAGCAAGCTATAAGGATACTAAAAGAGATCCGACAAC
TGACTTATGAAGAAGGCCATGATGATGGATTAATCTTCACTGGCTGTTATGACTCGTTTAAACCCATGAG
TGAAAACACCATCAACAAAGCCCTGCGTAATATGGGATATAACACGAAGCAGGACATCTGTGGACACGGT
TTCCGCACTCTGGCCTGTAGTGCCTTAATTGAGTCCGGGCTATGGTCAGAAGACGCTGTGGAGCTTCAAA

TGAGCCATAAGGAAAGTAACAGCGTCCGTGCTGCTTATACCCACAAGGCAAAACATCTTGAGCAACGTCG
CCTGATGCTCCAATGGTGGGCTGACTACCTTGATGCAAGCAGAAACGGTATGGTAAGGCCGTTTGAGTTT
GCTACAAATAAATAA

>CP009787.1:c153204-151906 Yersinia rohdei strain YRA
ATGTCACTGACTGATATTAAAGTAAAAAATGCAAAACCCCTTGAGAAGGAATACAAGCTTACTGATGGCT
TTGGTATGTTCCTTCGTGTTACCCCTAAAGGTTCGAAATACTGGCAAATGGCTTACCGCTTCGAAGGGAA
ACAAAAACTCTTCTCTATTGGTGTTTACCCTGCTGTTTCTCTTTCTGACGCAAGACAACGCCGTGACGAA
GCCAGAAGGCTTCTGGCTCAGGGTATTGACCCTAATGCAAAGAAACAGGCAGAGGTTAAAGAGCTTAAAG
CCAAACGTGATAATACACGCTCCTTCAGAACGGTAGCCAAAGCGTGGTTCTCCACGAAAACAAAATGGTC
TGATGATTATGGTGATGCCGTATGGAAGCGCCTTGAAACTTATGTCTTCCCGGTAATCGGTGACAAAGAT
GTTGCCGAACTGGATACGGGTGATCTGCTGGTTCCGGTGAAAAAAGTTGAGGCTCTTGGTTATCTTGAAG
TTGCCATGCGCATTCAACAATACATTACGGCAATCCTGCGTCATGCCGTCCAGCAAAAGCTGATACGCCA
TAACCCAGCCTATGATATGGAAGGTGCAGTTCAGAAACCACAAACTGAACACCGCCCTGCACTTGAGCTG
GAAGAAATACCCCAGCTACTGAACAAAATTGCCGAATACAAAGGCCGCAGGTTAACCATACTGGCAATAC
AGCTCAATCTGATGATTTTCATTCGTTCCAGTGAGTTGCGTTTCGCTCGCTGGTCAGAAATTGATTTCAA
AAGTAAGTTATGGGTGATACCCGAACAGCGTGAAGCGATTGAAAACGTCAAACATTCAACTCGTGGGGCT
AAAATGAAGCGTAAGCACTTCGTTCCCCTTTGTAAGCAGGCCATGAAGATACTCAAAGAGATCCGACAAC
TGACTTATGAAGAAGGCCATGATGATGGATTAATCTTTACAGGCTGTTATGACTCGTTTAAGCCAATGAG
CGAAAACACAATCAACAAAGCCCTCCGTAATATGGGCTATGACACGAAGCAGGACATCTGTGGACACGGT
TTCCGCACACTGGCCTGTAGTGCCTTAATTGAATCCGGTTTATGGTCAGAAGACGCTGTAGAGCTTCAGA
TGAGTCACAAGGAAAGCAATAGCGTCCGTGCGGCTTACACCCACAAAGCCAAACACCTTGACCAGCGCCG
CCTGATGCTTCAGTGGTGGGCTGACTACCTTGATGCAAGCAGAAACGGTATGGTCAGACCGTTTGAATTT
TCCACAGATGCTCGTTTGGGTTTAGAAAACCAAGCCTAA

>CP015749.1:2241234-2242511 Pectobacterium wasabiae strain RNS08.42.1A
ATGTCACTTACTGATACTAAAGTAAAAAATGCCAAGCCGTCAGAAAAGGCGGTGAAGCTCACTGACGGGT
TCGGCCTCTACCTGCTGGTACATCCCAACGGTTCCAAATACTGGCAGTTAGGCTATCGCTTTGAAGGGAA
ACAGAAGGTGTTTTCCATCGGCGTCTATCCTGCCGTATCTCTGGCTGATGCCAGACAACGGCGAGATGAG
GCGAAAAAGCTGTTAGCCGCTGGTATTGACCCAAGCGCCAAAAAGCAGGCTGATAACAAAATCGTTCAAG
AGAAGCGTAACAATACCCGCGCTTTCAAAACCGTTGCCAAAAGCTGGTTTGCCACCAAAACCACATGGTC
GGAAGATTATCAGCGTTCGGTATGGACCCGGCTGGAAACTTACCTGTTCCCTGATATTGGTGAAAGAGAT
ATTGCTGAACTGGATACAGGCGATCTGCTGTTTCCCATCAAAAAGATAGAGAAGCTGGGTTATCTTGAAA
TTGCCATGAGGGTGAAGCAGTACGCAACCGCCATCATGCGTTATGCCGTCCAGCAAAAAATGATCCGTTT
CAATCCGGCCTACGATTTGGAAGGTGCTGTTCAGAAGCCGCAAACGGAACACCGTCCCGCTATCGAACTG
GAAGAGATACCTACCCTATTGGAACGCATTGAAGCCTATAAAGGTCGTAGCAGGCTTACCCAATTGGCGA
TAAAACTCAATCTGCTAATTTTTGTGCGTTCCAGTGAACTCCGTTTTGCCCGTTGGTCAGAGATCGACTT
CAAAAGCGCCTTGTGGGTTATTCCTGAACAACGTGAAGCCATTGAAGGGATAAAGCATTCAGGTCGAGGT
GCCAAAATGCGTAGAAAACATTATGTTCCTCTATGCGATCAAGCACTGGCAATTTTAGAAGAGTTAAAAG
ACCTCACCTATGACGTTAACGGTGATGACGGCTTTATCCTGACTGGCTGTTATGATGCGATGAAACCTAT
GAGCGAAAACACCATCAACAAGGCACTGCGCAAAATGGGCTATGACACCAAAACCGATTTGTGTGGTCAT
GGTTTCCGAACGCTGGCGTGTAGTGCCTTAATTGAATCGGGGATCTGGCCTGAAGATGTGGTTGAGCTTC
AAATGAGCCACATGGAAAAGAACAACGTTCGCGCCGCCTATACTCATAAGGCCAAACATCTTGAACAACG
CCGCTTGATGTTGCAATGGTGGGCCGATTTTCTGGATGCGAACAGCAACGGTATGGTTAGGCCGTTTGAG
TTTATTTCGATAAAATAA

>CP009769.1:c1733890-1732613 Pectobacterium carotovorum subsp.
brasiliense strain BC1
ATGTCACTTACTGATACTAAAGTAAAAAATGCCAAGCCATCAGAAAAGGTGGTTAAGCTCACTGACGGGT
TTGGCCTCTACCTGCTGGTACATCCCAACGGTTCAAAATACTGGCAGTTAGGCTACCGCTTTGAAGGGAA
ACAGAAGGTGTTTTCCATCGGTGTCTATCCTGCTGTTTCGCTGGCTGATGCAAGACAACGCCGGGATGAA
GCAAAAAAGCTGTTAGCGGCGGGAATCGACCCCAGCGCCAAAAAACAGGCTGACAACAAAACCATTCAAG
AGAAGCGTAACAACACCCGCGCTTTCAAAACAGTTGCCAAAAGCTGGTTTGCCACCAAAACCACATGGTC
AGAAGATTATCAGCGTTCTGTATGGACCCGACTGGAAACCTATCTGTTCCCTGATATTGGCAACAAAGAT
ATTGCTGAACTGGATACAGGCGATCTGCTGGTTCCCATCAAAAAGATAGAAAAGCTGGGTTATCTGGAAA
TTGCCATGCGGGTAAAACAGTACGCAACCGCCATCATGCGTTACGCCGTTCAGCAAAAGATGATCCGTTT
CAATCCTGCCTATGATTTGGAAGGGGCAGTTCAGAAGCCACAGACGGAACACCGCCCCGCTATCGAACTG
GAAGAGATTCCTACCCTACTGGAACGTATTGACGGCTATCAGGGCCGTAGCAGACTTACCCAATTGGCAA

TAAAGCTCAATCTGCTGATTTTTGTCCGTTCCAGTGAACTCCGCTTTGCCCGTTGGTCAGAGATCGATTT
CAAAAGCGCTTTGTGGGTCATCCCTGAACAGCGTGAAGCCATAAAAGGGATCAAGCATTCAGGCCGTGGT
GCCAAAATGCGCAGGAAACATTATGTTCCTCTATGCGATCAAGCGCTGGCAATTTTGGAAGAGCTTAAAG
ACCTCACCTATGACGTTAACGGTGACGACGGCTTTATCCTGACTGGCTGTTATGATGCGATGAAGCCGAT
GAGCGAAAACACCATCAACAAGGCACTGCGCAAAATGGGTTATGACACCAAAACCGACCTGTGTGGTCAT
GGTTTCCGAACGCTGGCGTGTAGTGCCTTAATTGAATCGGGTATCTGGCCTGAAGATGTGGTTGAACTTC
AGATGAGCCACATGGAAAAGAACAACGTTCGCGCTGCCTACACTCACAAGGCCAAACACCTTGAGCAACG
CCGCCTCATGTTGCAATGGTGGGCTGATTTTCTGGATGCTAACAGCAACGGTATGGTCAGGCCGTTTGAG
TTTGCGCAGAAGGGATAG

>CP015749.1:2322845-2324122 Pectobacterium parmentieri strain
RNS08.42.1A
ATGTCACTTACTGACACCAAAGTAAAAAAAGCTAAACCTCTTGAGAAAGAATACAAGCTTACTGATGGTT
TTGGTATGCACCTGCTTGTCCACCCGAACGGCTCTAAATACTGGCGGCTTTCCTACCGCTTTGCACAAAA
GCAAAAACTGTTAGCGCTGGGGGTTTACCCTACGATTTCTTTGACTGACGCAAGGGAGCGTCGTGATGAA
GCGCGTAAGCTGATTGCTTATGGGATTGATCCAGGAGCCAGAAAAAAATCAGGCCGTGAGCGACAAGGAA
CACACGATGAATCTCGCTCCTTTGCTATAATGGCTCGTGCATGGGCTGAGACCAAAACCAAGTGGTCAGA
AGACTACAAAGTTAAGGTCTGGAGACGCATTGAAAACTACCTTCTGCCAGATCTGGGAAATCGTGATGTA
TCAGAGCTTGATACCAGCGATTTACTCATCCCCCTCAGAAAAGTTGAGAAATTAGGCTATCTCGACATCG
CCATGCGCCTGAAGCAGTATACAACCTCTATCATGCGATACGTCGTCCAGCAGAAAATCATCAGTTACAA
CCCTGCTTATGATTTGCAAGGCACTATCGAAAAAGGTGAGACAGCGCACCGCCCTTCTATCGAAATCTAT
GAAATTCCCGATCTCTTACAAAAACTCGACAACTACCGTGGTCGGGGTCTTTTAACAGAATTAGCGATCA
AACTCACATTATTGGTTTTTGTCAGGTCAAGTGAGTTACGTTTTGCCCGATGGAGTGAAATCGACTTCAA
AAAATCTCTCTGGGTTATCCCTGAACAACGCAAGGAAGTTAAAGGGGTAAAACACTCAGGTCGTGGTGCC
AAAATGAAGAGAAAGCATTTTGTCCCACTGTGCAGGCAGGCAGTTGAGATCTTGAAAGAGGTTAAACAGA
TCACTTATGGTGAAAAAGCGGGTGACGGGTTTATTTTCACCGGGTTTTACGACAGTGATTCTGCGATGAG
TTCAGGCACTATCAATAAAGCCCTTCAGCGTATGGGATACGACACCAAAACGGATCTGTGTGGGCATGGC
TTTCGCACGTTAGCCTGTAGTGCATTGACGGAATCAGGATTATGGTCGGAAGACACGGTTGAGCTTCAAA
TGAGCCATAAAGAGAAAAACACCGTTAGATCTGCTTATACCCATAAAGTCAGTCATCTTGACCAGCGTAA
ACTGATGCTGCAATGGTGGGCTGATTTTCTGGATGCGAACCGCAATGGAGTAGTTAGCCCGTTTGAGTTT
GCCAACAGGAAGCAATAA

>CP001790.1:c1909931-1908654 Pectobacterium parmentieri WPP163
ATGTCACTTACTGATACTAAAGTAAAAAATGCCAAGCCGTCAGAAAAGGTGGTTAAGCTCACTGACGGGT
TCGGCCTCTACCTGTTGGTGCATCCCAACGGTTCCAAATACTGGCAGTTAGGCTACCGCTTCGAAGGGAA
ACAGAAGGTGTTTTCGATCGGCGTCTACCCTGCTGTTTCACTGGCTGATGCAAGACAACGCCGGGATGAC
GCCAAAAAGCTGTTGGCTGCTGGCATTGATCCGAGCGCCAAAAAACGGGCTGACAACAAGTCCGTTCAAG
AAAAGCGGAACAATACCCGCGCTTTCAAAACCGTTGCCAAAAGCTGGTTTGCCACCAAAACCACATGGTC
GGAAGATTATCAGCGTTCTGTATGGACCCGACTGGAAACCTATCTGTTCCCTGATATTGGCAACAAAGAT
ATTGCTGAACTGGATACAGGCGATCTGCTGGTTCCCATCAAAAAGATAGAAAAGCTGGGTTATCTGGAAA
TTGCCATGCGGGTAAAACAGTACGCAACCGCCATCATGCGTTACGCCGTTCAGCAAAAGATGATCCGTTT
CAATCCTGCCTATGATTTGGAAGGGGCAGTTCAGAAGCCACAGACGGAACACCGCCCCGCTATCGAACTG
GAAGAGATTCCTACCCTACTGGAACGTATTGACGGCTATCAGGGCCGTAGCAGACTTACCCAATTGGCAA
TAAAGCTCAATCTGCTGATTTTTGTCCGTTCCAGTGAACTCCGCTTTGCCCGTTGGTCAGAGATCGATTT
CAAAAGCGCTTTGTGGGTCATCCCTGAACAGCGTGAAGCCATAAAAGGGATCAAGCATTCAGGCCGTGGT
GCCAAAATGCACAGGAAACATTATGTTCCTCTATGCGATCAAGCGCTGGCAATTTTGGAAGAGCTTAAAG
ACCTCACCTATGACGTTAACGGTGACGACGGCTTTATCCTGACTGGCTGTTATGATGCGATGAAGCCGAT
GAGTGAAAACACCATCAACAAGGCACTGCGCAAAATGGGCTATGACACCAAAACCGATCTGTGCGGTCAT
GGTTTCCGAACGCTGGCGTGTAGTGCGTTAATCGAATCAGGTATCTGGCCTGAAGATGTGGTTGAACTTC
AGATGAGCCACATGGAAAAGAACAACGTTCGTGCCGCCTACACTCACAAGGCCAAACATCTTGAACAGCG
TCGTCTGATGTTGCAATGGTGGGCTGATTTTCTGGATGCTAACAGCAACGGTATGGTGAGGCCGTTTGAG
TTTTCCAATCGGTCATAG

>CP009125.1:c1865896-1864619 Pectobacterium atrosepticum strain 21A
ATGTCACTTACTGATACTAAAGTAAAAAATGCCAAGCCGTCAGAAAAGGCGGTGAAGCTCACTGACGGGT
TCGGCCTCTACCTGCTGGTGCATCCCAACGGTTCAAAATACTGGCAGTTAGGCTATCGCTTTGAAGGAAA
ACAGAAGGTGTTTTCCATCGGTGTCTACCCTGCTGTTTCACTGGCTGGTGCAAGACAACGCCGGGATGAA
GCAAAAAAGCTGTTAGCGGCGGGAATTGATCCGAGCGCTAAAAAGCAGGCAGACAACAAAATCGTTCAAG

AGAAGCGTAACAACACCCGCGCTTTCAAAACCGTTGCCAAAAGTTGGTTTGCCACCAAAACCACATGGTC
GGAAGATTATCAGCGTTCTGTCTGGACACGACTGGAAACTTATCTGTTCCTCGACATCGGTAACAAGGAC
ATTGCTGAACTGGATACAGGCGATCTGCTGGTTCCCATCAAAAAGATAGAGAAGCTGGGTTATCTGGAAA
TTGCTATGCGGGTGAAACAGTACACCACCGCCATCATGCGTTATGCCGTCCAGCAAAAGATGATCCGTTT
CAATCCAGCCTATGATTTGGAAGGTGCGGTTCAGAAGCCACAGACGGAACACCGCCCCGCTATTGAACTG
GAAGAGATACCTACCCTGCTGGAACGCATTGAGGGCTATCAGGGCCGTAGCAGACTGACCCAATTGGCGA
TAAAACTCAATCTGCTGATTTTTGTGCGTTCCAGTGAACTCCGCTTTGCCCGATGGTCAGAGATCGATTT
CAACAGTACTTTGTGGGTTATTCCTGAACAGCGTGAAACCATTAAAGGGATCAAGCATTCAGGCCGTGGT
GCCAAAATGCGCAGGAAGCATTATGTCCCCTTGTCCAATCAGGCACTGGCAATTTTGGCAGAACTAAAAG
ACCTCACCTATGACGTTAACGGTAATGACGGCTTTATCCTGACTGGCTGTTATGATGCGATGAAGCCAAT
GAGTGAAAACACCATCAACAAAGCACTACGCAAAATGGGCTATGACACCAAAACCGATTTGTGTGGTCAT
GGTTTCCGAACGCTGGCGTGTAGTGCCTTAATTGAATCGGGTATTTGGCCTGAAGACGTGGTTGAGCTTC
AAATGAGCCATATGGAAAAGAACAACGTTCGCGCTGCCTACACTCACAAGGCCAAACATCTTGAACAACG
TCGCCTGATGCTGCAATGGTGGGCTGATTTTCTGGACGCTAACCGGAATGGGATGGTCAGGCCGTTTGAG
TTTACACATCAAACATAA

>CP003415.1:c1821846-1820572 Pectobacterium sp. SCC3193
ATGTCACTTACTGACACCAAAGTAAAAAAAGCTAAACCTCTTGAGAAAGAATACAAGCTTACTGATGGTT
TTGGTATGCACCTGCTTATCCACCCGAACGGATCTAAATACTGGCGGCTTTCCTACCGCTTTGCACAGAA
GCAAAAACTGTTAGCGTTGGGCGTTTACCCTGCTATTTCTTTGACTGACGCAAGGGAGCGTCGTGATGAA
GCGCGTAAGCTGATTGCTAATGGGATTGATCCAGGAGCCAGAAAAAAATCAGGCCGGGAGCAACAAGGAA
CACACGATGAGTCTCGCTCCTTTGCTGTAATGGCTCGTGCATGGGCTGAGACCAAAACCAAGTGGTCAGA
AGACTACAAAGTTAAGGTCTGGAGACGCATTGAAAACTACCTCCTGCCAGATCTGGGAAATCGTGATGTA
TCAGAGCTTGATACCAGCGATTTACTCATCCCCCTAAGAAAAGTTGAGAAATTAGGCTATCTCGACATCG
CCATGCGCCTGAAGCAGTATACAACCTCTATCATGCGATACGCCGTCCAGCAGAAGATCATCAGTTATAA
CCCTGCTTATGATTTGCAAGGCACTATCGAAAAAGGTGAGACAGCACACCGCCCTTCTATCGAAATCTAT
GAAATTCCCGATCTCTTACAAAAACTCGACAACTACCGTGGTCGGGGGCTTTTAACAGAATTAGCGATCA
GGCTCACATTATTGGTTTTTGTCAGGTCAAGTGAGTTACGTTTTGCCCGATGGAGTGAAATCGACTTCAA
AAAATCTCTCTGGGTTATCCCTGAACAACGCAAAGAAGTTAAAGGGGTAAAACACTCTGGTCGTGGTGCC
AAAATGAAGAGAAAGCATTTTGTCCCACTGTGCAGGCAGGCGGTTGAGATCTTGAAAGAGGTTAAACAGA
TCACTTATGGTGAAAAAGCGGGTGACGGGTTTATTTTCACCGGGTTTTACGACAGTGATTCTGCGATGAG
TTCAGGCACTATCAATAAAGCCCTTCAGCGTATGGGATACGACACCAAAACCGATCTGTGTGGGCATGGC
TTTCGCACGTTAGCCTGTAGTGCATTGACAGAATCGGGATTATGGTCGGAAGACACGGTTGAGCTTCAAA
TGAGCCATAAAGAAAAAAACACCGTTAGATCTGCTTATACCCATAAAGTCAGTCATCTTGACCAGCGTAA
ACTGATGCTGCAATGGTGGGCTGATTTTCTGGATGCGAACCGCAATGGAGTAGTTAGCCCGTTTGAGTTT
GCACAGAAAGGATAG

>CP021894.1:c1734851-1733577 Pectobacterium carotovorum strain SCC1
ATGTCACTTACTGACACCAAAGTAAAAAAAGCTAAACCTCTTGAGAAAGAATACAAGCTTACTGATGGTT
TTGGTATGCACCTGCTTATCCACCCGAACGGATCTAAATACTGGCGGCTTTCCTACCGCTTTGCACAGAA
GCAAAAACTGTTAGCGTTGGGCGTTTACCCTGCTATTTCTTTGACTGACGCAAGGGAGCGTCGTGATGAA
GCGCGTAAGCTGATTGCTAATGGGATTGATCCAGGAGCCAGAAAAAAATCAGGCCGGGAGCAACAAGGAA
CACACGATGAGTCTCGCTCCTTTGCTGTAATGGCTCGTGCATGGGCTGAGACCAAAACCAAGTGGTCAGA
AGACTACAAAGTTAAGGTCTGGAGACGCATTGAAAACTACCTCCTGCCAGATCTGGGAAATCGTGATGTA
TCAGAGCTTGATACCAGCGATTTACTCATCCCCCTAAGAAAAGTTGAGAAATTAGGCTATCTCGACATCG
CCATGCGCCTGAAGCAGTATACAACCTCTATCATGCGATACGCCGTCCAGCAGAAGATCATCAGTTATAA
CCCTGCTTATGATTTGCAAGGCACTATCGAAAAAGGTGAGACAGCACACCGCCCTTCTATCGAAATCTAT
GAAATTCCCGATCTCTTACAAAAACTCGACAACTACCGTGGTCGGGGGCTTTTAACAGAATTAGCGATCA
GGCTCACATTATTGGTTTTTGTCAGGTCAAGTGAGTTACGTTTTGCCCGATGGAGTGAAATCGACTTCAA
AAAATCTCTCTGGGTTATCCCTGAACAACGCAAAGAAGTTAAAGGGGTAAAACACTCTGGTCGTGGTGCC
AAAATGAAGAGAAAGCATTTTGTCCCACTGTGCAGGCAGGCGGTTGAGATCTTGAAAGAGGTTAAACAGA
TCACTTATGGTGAAAAAGCGGGTGACGGGTTTATTTTCACCGGGTTTTACGACAGTGATTCTGCGATGAG
TTCAGGCACTATCAATAAAGCCCTTCAGCGTATGGGATACGACACCAAAACCGATCTGTGTGGGCATGGC
TTTCGCACGTTAGCCTGTAGTGCATTGACAGAATCGGGATTATGGTCGGAAGACACGGTTGAGCTTCAAA
TGAGCCATAAAGAAAAAAACACCGTTAGATCTGCTTATACCCATAAAGTCAGTCATCTTGACCAGCGTAA
ACTGATGCTGCAATGGTGGGCTGATTTTCTGGATGCGAACCGCAATGGAGTAGTTAGCCCGTTTGAGTTT
GCACAGAAAGGATAG

>CP020358.1:1490733-1492007 Klebsiella oxytoca strain AR_0147
ATGTCACTGACTGATATTAAAGCAAAAAATGCAAAACCCCTTGAGAAGGAATACAAGCTTACTGATGGCT
TTGGTATGTTCCTTCGTGTTACCCCTAAAGGTTCGAAATACTGGCAAATGGCTTACCGCTTCGAAGGGAA
ACAAAAACTCTTCTCTATTGGTGTTTACCCTGCAGTTTCTCTTTCTGACGCAAGACAACGCCGTGACGAG
GCCAGAAGGCTTCTGGCTCAGGGTATTGACCCTAATGCAAAGAAACAGGCAGAGGTTAAAGAGCTTAAAG
CCAAACGTGATAATACACGCTCCTTCAGAACAGTAGCCAAAGCGTGGTTCTCTACGAAAACAAAATGGTC
TGATGATTATGGTGATGCCGTATGGAAGCGCCTTGAAACTTATGTCTTCCCGGTAATCGGTGACAAAGAT
GTTGCCGAACTGGATACGGGTGATCTGCTGGTTCCGGTGAAAAAGGTTGAGGCACTTGGTTATCTTGAAG
TTGCCATGCGCATTCAACAATACATTACGGCAATCCTGCGTCATGCCGTCCAGCAAAAGCTGATACGCCA
TAACCCAGCCTATGATATGGAAGGTGCAGTTCAGAAACCACAAACTGAACACCGCCCTGCACTTGAGCTG
GAAGAAATACCCCAGCTACTGAACAAAATTGCCGAATACAAAGGCCGCAGGTTAACCATACTGGCAATAC
AGCTCAATCTGATGATTTTCATTCGTTCCAGTGAGCTTCGTTTCGCTCGCTGGTCTGAAATTGATTTCAA
AAGTAAGTTATGGGTGATACCCGAACAGCGTGAAGCAATTGAAAACGTCAAACATTCGACTCGTGGGGCT
AAAATGAAGCGTAAGCACTTCGTTCCCCTTTGTAAGCAGGCCATGAAGATACTCAAAGAGATCCGACAAC
TGACTTATGAAGAAGGTCAAGATGATGGGTTAATCTTTACTGGCTGTTATGACTCGTTTAAGCCCATGAG
TGAAAACACCATCAACAAAGCCCTCCGCAATATGGGCTATGACACGAAGCAGGACATCTGTGGACACGGT
TTCCGAACACTGGCCTGTAGTGCCTTAATTGAGTCCGGGCTATGGTCAGAAGACGCTGTAGAGCTTCAGA
TGAGCCATAAGGAAAGCAACAGTGTCCGGGCAGCTTATACCCACAAGGCTAAACACCTTGAACAGCGCCG
CCTGATGCTTCAGTGGTGGGCTGATTTCCTTGATGCTAACAGCAATCATATGGTCAGGCCGTTTGAGTTT
GCTTCAAATAAATAA

>BX950851.1:c1926959-1925682 Pectobacterium atrosepticum SCRI1043
ATGTCTCTAACTGATTCCAAAGTAAAAAATGCCAAATCTCTTGAGAAGGAATATAAGCTTACTGATGGCT
TTGGTATGCACCTGCTAGTGCATCCTAATGGATCTAAATACTGGCGCTTGTCTTATCGTTTCGAAAAGAA
GCAAAGATTACTCGCTTTAGGCGTCTATCCTGCTGTTTCTTTGGCTGATGCCAGACAACGCCGGGATGAA
GCGAAAAAGCTGTTAGCTGCTGGTATTGACCCCAGCGCTAAAAAGCAGGCTGACAACAAAACCATTCAAG
AGAAGCGTAACAATACCCGCGCTTTCAAAACCGTCGCCAAAAGCTGGTTTTCCACCAAAACCACATGGTC
GGAAGATTATCAGCGTTCTGTCTGGACACGACTGGAAACTTATCTGTTCCCTGATATTGGCAACAAAGAT
ATTGCTGAACTGGATACAGGCGATCTGCTGGCTCCCATCAAAAAGATAGAGAAGCTGGGTTATCTGGAAA
TCGCCATGCGGGTGAAACAGTACACGACCGCCATCATGCGTTATGCCGTCCAGCAAAAGATGATCCGTTT
CAATCCAGCCTATGATTTGGAAGGTGCGGTTCAGAAGCCTCAGACGGAACACCGCCCCGCTATCGAGCTT
GAAGAGATCCCTACCCTACTGGAACGTATTGAAGGCTATCAGGGGCGTAGCAGACTGACCCAATTGGCGA
TAAAACTCAATTTGCTGATTTTTGTGCGTTCCAGTGAACTCCGCTTTGCCCGTTGGTCAGAGATCGATTT
CAAAAGTGCTTTATGGGTTATCCCTGAACAACGTCAAGCCATTGAAGGGATAAAGCATTCAGGTCGGGGT
GCCAAAATGCGCAGGAAACATTATGTTCCTCTATGCGATCAAGCACTGGCAATTTTGGAAGAGCTTAAAG
ACCTCACCTATGACGTTAACGGTGATGACGGCTTTATCCTGACTGGCTGTTATGATGCGATGAAGCCGAT
GAGTGAAAACACCATCAACAAGGCGCTTCGTAAAATGGGCTATGACACCAAAACCGATCTGTGCGGCCAC
GGCTTTCGAACGCTGGCGTGTAGTGCATTAATTGAATCAGGTATCTGGCCGGAAGACGTGGTTGAACTTC
AGATGAGCCACATGGAAAAGAACAACGTTCGTGCTGCCTACACTCACAAGGCCAAACACCTTGAGCAACG
CCGCCTGATGTTGCAATGGTGGGCTGATTTTCTTGATGCTAACCGCAACGGGATGGTTAGGCCGTTTGAA
TTTGCGCAGAAGGGATAA

>AL513382.1:c4542913-4541654 Salmonella enterica subsp. enterica
serovar Typhi str. CT18
ATGTTGACTGACAGCAAGATCCGCGCCGCGAAACCCCTCGCAAAATCCTATAAACTCACTGATGCACAAG
GTCTGTACCTGACGGTATCCACCAGTGGTTCAAAGCTATGGTATTTCCGCTACCGTTTTGAAGGTAAGGA
AAATCGTCTGGCCTTCGGCCCCTATCCGCAGGTTACGCTGGCGGAAGCCCGCGAAAAGCGCGATGCGGCG
CGTAAGCTGCTGGTATCCGGCGTTTGCCCTTCTCTGCCCCGCAAGGCGGAAAAAGCCGCCGTTGACGGTA
CGCGCACCTTTCAGTACATCGCCACGGCGTGGCACTCCAGTTGTCTTAAGCTGTGGTCAGAAGACCACGC
GGACAAGATCCTGACCTGCCTGAAGCGCTACGTCTTCCCCGATATTGGCGCAATGGACATTGCAGAGGTT
GAAACCCGCCATCTGGCGCAGCTTGTTAAATCCATCGACGATAAGGGCGTGCATGACGTTGCCGGGCGGG
TGCGCCAGCATCTGACCAAAATCATGCGTCATGCCGTACAGCAGGGAACGATTAAATATAATCCGGCTTA
CGATCTGGATGGCGTCGTGACCCCTGTTGTGACCCAACATCACCCCGCCCTGCCCCTGAAACGCCTGCCG
GAGCTGCTGGACAAGATTAATGGCTACAAAGGGCGGGAACTGACCCGTCTGGCGCTGGAGTTGAATCTGC
ACGTTTTCCTGCGCTCCAGTGAGTTACGCCTCGCCCGCTGGGATGAGTTCAACCTGAAAGCTCGTATCTG
GACGGTGCCAGCAAAGCGGGAAGCGGTGAAGAACGTGCGTTTCTCAGAGCGTGGCGCAAAGATGAAGGAT
GAGCATCTGGTGCCGCTATCTGCGCAGGCTGTCGCCCTGCTGGAGCAGATAAAGGAAATTACCGGAGAGT
CGGTATTTGTTTTTGCGGGTGCTCACTCAATGAACAAGCCGATGAGTGAAAACACCATCAACAAGGCGCT

GCGCGTGATTGGTTACGACACCAAAACCGAAGTCTGCGGTCACGGATTCAGAACGATGGCCTGTAGCGCC
CTGAACGAATCCGCACTGTGGTCTAAGGACGCCATCGAGCGCCAGATGAGCCACAAGGAGCGCAACGGCG
TGCGGGCGGCGTATGTGCATAAGGCGGAGCATCTGGAAGCGCGTATGGAGATGATGCAGTGGTGGTCGGA
TTATCTCGACATGAGCCGCGAGGGGTACGTCGCGCCGTATATTTATGCGCGGCGGCATAAGGCTGCCTGA

>NC_001609.1:c3926-2607 Enterobacteria phage P4
ATGTGCCCCCAAATGAAGCTCAACGCCAGACAGGTCGAGACCGCAAAGCCAAAAGACAAAACCTACAAAA
TGGCCGATGGTGGTGGTTTGTATCTTGAGGTTTCGGCCAAAGGTTCCAAATACTGGCGCATGAAATACAG
ACGTCCCTCTGACAAAAAAGAGGATCGTCTTGCGTTTGGTGTTTGGCCTACTGTGACGCTTGCTCAGGCA
AGAGCAAAACGCGATGAAGCTAAAAAGCTTTTAGTACAGGGCATAGACCCAAAAGTCGTACAAAAAGAAG
CTCGGGCCGAGAATTCGGGGGCATATACTTTTGAAGCTATCGCCCGAGAATGGCATGCCAGTAACAAGCG
CTGGAGTGAAGACCATCGATCGCGCGTTCTTCGCTATCTTGAGCTTTATATATTCCCTCATATCGGTTCG
TCCGACATTCGGCAGCTTAAAACCAGCCACCTGTTAGCCCCGATTAAAAAAGTTGATGCCAGTGGCAAAC
ACGATGTCGCTCAGCGCCTGCAACAGCGCGTCACAGCCATTATGCGTTATGCCGTACAGAACGATTACAT
CGACTCAAATCCAGCCAGCGATATGGCTGGTGCGCTATCGACAACCAAAGCGCGACATTACCCTGCTTTA
CCTTCTAGCCGATTCCCTGAGTTTCTTGCACGTCTTGCTGCATATCGTGGCCGTGTAATGACACGGATTG
CGGTCAAGCTTTCCTTGCTAACTTTTGTGCGTTCCAGTGAATTACGTTTCGCACGTTGGGATGAATTCGA
CTTCGATAAATCTCTTTGGCGTATACCTGCAAAGCGAGAAGAAATTAAAGGTGTGCGTTATTCGTACCGA
GGCATGAAGATGAAAGAGGAACATATCGTTCCGCTTAGTCGACAGGCGATGATTTTATTAAACCAGTTAA
AGCAGATTAGTGGTGATAAAGAGCTGCTTTTTCCGGGGGATCATGACGCAACTAAGGTTATGAGTGAAAA
CACGGTAAACAGCGCATTGCGTGCGATGGGCTATGATACTAAAACCGAGGTGTGTGGGCATGGGTTTAGG
ACTATGGCGCGTGGTGCGTTGGGGGAGTCGGGGTTATGGAGTGATGACGCGATAGAGAGGCAACTGAGCC
ACTCAGAGCGTAATAATGTACGTGCGGCATATATCCATACCTCCGAGCATTTGGATGAGCGGCGTTTAAT
GATGCAGTGGTGGGCTGATTATTTGGATATGAACCGTAATAAATATATTAGCCTTATGATTATTCAAAAT
ACAAAGAAATACTTAAATAAAAATAGTTATTGGTTGATTTTTAAAATGAGTGTTAAGTAA
